# Genome sequences reveal global dispersal routes and suggest convergent genetic adaptations in seahorse evolution

**DOI:** 10.1038/s41467-021-21379-x

**Published:** 2021-02-17

**Authors:** Chunyan Li, Melisa Olave, Yali Hou, Geng Qin, Ralf F. Schneider, Zexia Gao, Xiaolong Tu, Xin Wang, Furong Qi, Alexander Nater, Andreas F. Kautt, Shiming Wan, Yanhong Zhang, Yali Liu, Huixian Zhang, Bo Zhang, Hao Zhang, Meng Qu, Shuaishuai Liu, Zeyu Chen, Jia Zhong, He Zhang, Lingfeng Meng, Kai Wang, Jianping Yin, Liangmin Huang, Byrappa Venkatesh, Axel Meyer, Xuemei Lu, Qiang Lin

**Affiliations:** 1 CAS Key Laboratory of Tropical Marine Bio-Resources and Ecology, South China Sea Institute of Oceanology, Innovation Academy of South China Sea Ecology and Environmental Engineering, Chinese Academy of Sciences, Guangzhou, China; 2 Southern Marine Science and Engineering Guangdong Laboratory (Guangzhou), Guangzhou, China; 3 Laboratory for Marine Fisheries Science and Food Production Processes, Pilot National Laboratory for Marine Science and Technology (Qingdao), Qingdao, China; 4 Department of Biology, University of Konstanz, Konstanz, Germany; 5 Beijing Institute of Genomics, Chinese Academy of Sciences; China National Center for Bioinformation, Beijing, China; 6 University of Chinese Academy of Sciences, Beijing, China; 7 Marine Ecology, Helmholtz Centre for Ocean Research Kiel, Kiel, Germany; 8 College of Fisheries, Key Laboratory of Freshwater Animal Breeding, Ministry of Agriculture and Rural Affairs, Huazhong Agricultural University, Wuhan, China; 9 Allwegene Technologies Inc., Beijing, China; 10 State Key Laboratory of Genetic Resources and Evolution, Kunming Institute of Zoology, Center for Excellence in Animal Evolution and Genetics, Chinese Academy of Sciences, Kunming, China; 11 BGI-Qingdao, BGI-Shenzhen, Qingdao, China; 12 School of Agriculture, Ludong University, Yantai, China; 13 Institute of Molecular and Cell Biology, A*STAR, Biopolis, Singapore, Singapore; 14 Present Address: Argentine Dryland Research Institute, National Council for Scientific and Technical Research (IADIZA-CONICET), Mendoza, Argentina; 15 Present Address: Department of Organismic and Evolutionary Biology, Harvard University, Cambridge, MA USA

**Keywords:** Phylogenetics, Population genetics, Genomics

## Abstract

Seahorses have a circum-global distribution in tropical to temperate coastal waters. Yet, seahorses show many adaptations for a sedentary, cryptic lifestyle: they require specific habitats, such as seagrass, kelp or coral reefs, lack pelvic and caudal fins, and give birth to directly developed offspring without pronounced pelagic larval stage, rendering long-range dispersal by conventional means inefficient. Here we investigate seahorses’ worldwide dispersal and biogeographic patterns based on a de novo genome assembly of *Hippocampus erectus* as well as 358 re-sequenced genomes from 21 species. Seahorses evolved in the late Oligocene and subsequent circum-global colonization routes are identified and linked to changing dynamics in ocean currents and paleo-temporal seaway openings. Furthermore, the genetic basis of the recurring “bony spines” adaptive phenotype is linked to independent substitutions in a key developmental gene. Analyses thus suggest that rafting via ocean currents compensates for poor dispersal and rapid adaptation facilitates colonizing new habitats.

## Abstract (web summary)

A new seahorse genome and the re-sequenced genomes of 21 other species shed new light on the evolutionary origin and global dispersal routes of seahorses, and show that bony spines—a key adaptation against predation—probably evolved multiple times via independent substitutions in the *bmp3* gene.

## Introduction

Explaining mechanisms of marine biodiversification is challenging, owing to persistent paucity of information on patterns of speciation and phylogeography in marine ecosystems^1–3^. Major geological vicariance events, such as the closure of the Panama seaway^4^ or the Tethys seaway^5,6^, have been suggested to impact patterns of marine biodiversification, particularly for organisms whose dispersal strategies rely on ocean currents transporting pelagic larvae or rafting individuals across large distances^7^. In such lineages, ecomorphological divergence and local adaptation after a colonization event can be slow even in the presence of strong divergent selective pressures^8^. Thus, comprehensive studies addressing spatio-temporal diversification patterns that include dynamics of geophysical processes, as well as knowledge of the genetic bases and developmental mechanisms of key adaptive traits, are required to understand the mechanisms that drive the evolution of marine biodiversity.

The radiation of seahorses (Family *Syngnathidae*) is a particularly iconic and suitable model system to investigate the effects that tectonic activity and ocean current dynamics can have on the dispersal and diversification of marine taxa due to the seahorses’ dispersal by rafting^7,9^, as well as to study the rapid evolution of adaptive phenotypes in new environments. Seahorse genomes evolve under some of the highest mutation rates among teleosts^10^ and have the greatest diversification rates within their family (Supplementary Fig. 1, Figshare: Dataset 1). All seahorses are sedentary but exhibit specialized morphological and life-history traits^11–13^, such as a prehensile tail (and the lack of a caudal fin), an elongated snout, lack of pelvic fins, an armor of bony plates instead of scales, and a unique mode of male pregnancy whereby males give birth to developed juveniles^14,15^. Species of seahorses differ widely in body size, color patterns and other adaptive traits to their respective environments^11^, such as the presence or absence of bony spines, which are likely an adaption against predators^16^.

Previous research revealed that the evolutionary origin of seahorses likely lies in the Late Oligocene’s Indo-Pacific^17–19^ from where different lineages dispersed around the globe despite the seahorses’ poor endurance swimming abilities and their reliance on rafting as primary long-distance dispersal strategy^9,20^. Nonetheless, a comprehensive understanding of the seahorses’ colonization routes is still missing as phylogenetic reconstructions were typically either derived only from relatively few species and/or few genetic markers^18,21–23^.

Here, we study the diversification patterns of these unique fishes based on the analysis of multiple sequenced seahorse genomes. By conducting comprehensive phylogenetic analyses, we infer their demographic history and clarify the role of seaway closures during their diversification as part of tracing the colonization routes from the origin of their common ancestor to their current distribution. Additionally, we address the adaptive phenotypic evolution of seahorses by studying the development of one of the most eye-catching traits within the genus: the presence or absence of bony spines.

## Results and discussion

### Global diversity of seahorses

**Fig. 1:**
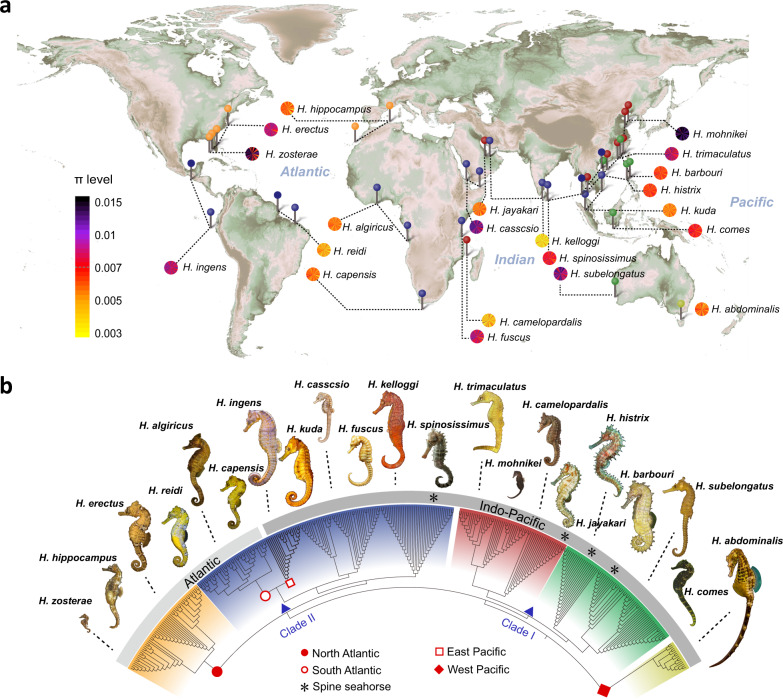
Genetic diversity and phylogenetic relationships of 358 seahorse specimens. **a** Geographic sampling locations for sampled seahorses with patterns of nucleotide diversity (*π*) of the 21 seahorse species across 22 chromosomes. Maps from Wessel et al. (2013) under GNU GPL license^91^. **b** Neighbor-joining tree constructed with genome-wide SNPs of 358 seahorses. Location pin symbols in (**a**) and branch background in (**b**) correspond to each other. Seahorses illustrations by Geng Qin. Source data are provided as a Source Data file.

Using PacBio long-read sequencing (~115-fold coverage), Illumina short-read sequencing (~243-fold coverage), and Hi-C technology (~184-fold coverage) we de novo assembled the genome of a male *Hippocampus erectus*. With a contig N50 of 15.5 Mb, our chromosome-level assembly (total size 420.66 Mb; comprising 22 superscaffolds corresponding to the expected chromosome number) (Supplementary Figs. 2–4, Supplementary Tables 1–4, and Supplementary Data 1) improved in sequence contiguity over previously available assemblies generated from Illumina short reads alone (contig N50: 14.57 kb)^10,24^. We re-sequenced the genomes (~16-fold coverage) of 358 seahorse specimens comprising 21 species reflecting *Hippocampus*’ global distribution, with representatives of major seahorse lineages (Fig. 1a, Supplementary Fig. 5a, Supplementary Data 2).

Our analysis identified each seahorse species as a monophyletic group in a neighbor-joining tree inferred from 41 million genome-wide single nucleotide polymorphisms (SNPs) (Fig. 1b, Supplementary Tables 5–8), and they formed distinct clusters in a principal component analysis (Supplementary Fig. 5b). Genetic diversity (*θπ* and *θω*) varied substantially among species and chromosomes, as it was, for example, generally higher for seahorses in the North Atlantic Ocean biome than in the South Atlantic Ocean biome (Fig. 1a, Supplementary Figs. 6, 7, Figshare: Dataset 2).

**Fig. 2:**
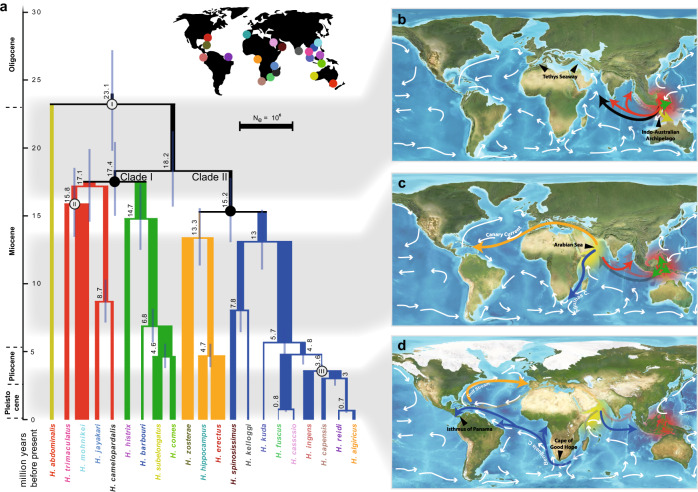
Colonization and demographic history of seahorses. **a** Phylogenetic tree and divergence time estimates for 21 seahorse species. The branch line thickness corresponds to the population size estimates (*N*_*e*_) and colors indicate different lineages. Symbols I–III indicate calibration points. **b**–**d** Predicted colonization routes (colored arrows) of seahorses based on divergence time, distribution, vicariance events, and ocean currents (white arrows). Maps modified from Ron Blakey © 2016 Colorado Plateau Geosystems Inc (License # 60519). **b** The Indo-Australian Archipelago was the center of origin (red marking) of the genus *Hippocampus* before seahorses diversified and dispersed globally 18–23 Ma. **c** Seahorses initially colonized the Atlantic Ocean through the opening Tethyan seaway, which, after its closure (Terminal Event during 7–13 Ma), separated this Tethyan lineage from its Indian Ocean sister lineage. The latter, subsequently rapidly diversified (yellow marking) in the Arabian Sea, establishing a second center of seahorse diversification. **d** A second seahorse colonization event of the Atlantic Ocean occurred from the Indian Ocean about 5 Ma by passing the South African tip, and finally arriving in the East Pacific Ocean through the still open Panama seaway approximately 3.6 Ma. Source data are available at Figshare (Datasets 4–6).

The time-calibrated tree estimated that the common ancestor of all extant seahorses lived ~20–25 Ma (million years ago) (Fig. 2a, Supplementary Figs. 8, 9, Figshare: Datasets 3–6), which coincides with the beginning of a period of explosive diversification in most modern marine fish and coral lineages^25,26^. The Indo-Australian Archipelago was identified as the center of origin of the genus *Hippocampus*, in line with previous studies^18,19^ (Fig. 2b, Supplementary Fig. 9). Subsequently, seahorses diversified and spread globally, with their colonization routes and dynamics strongly linked to prevalent oceanic currents and tectonic events (see Supplementary Text)^27^. Our species tree based on 2,000 loci suggests that *H. abdominalis* is the sister-lineage to a clade containing all other seahorses, and the latter are subdivided into two major phylogenetic clades: clade I comprises eight species exclusively inhabiting the Indo-Pacific Ocean, while clade II includes six species inhabiting the Atlantic Ocean, one from the East Pacific Ocean, and five from the Indo-Pacific Ocean (Fig. 2a, Supplementary Fig. 9). A more detailed description of clade II exemplifies the seahorses’ dependence on ocean currents as a means of far-distance dispersal and showcases how temporal seaways can boost or limit diversification and dispersal.

### Rapid diversification and colonization routes of clade II

After separating from clade I by dispersing into the West Indian Ocean around 18.2 Ma, the ancestors of the South Atlantic and North Atlantic lineages diverged from each other approximately 15.2 Ma (Fig. 2a). The North Atlantic lineage followed north-westward oceanic currents and passed through the Tethys Sea a few million years before the initial closure of the East Tethys Seaway due to tectonic shifts about 14 Ma^6,28^. Consistent with this colonization route for the North Atlantic lineage a strong genetic bottleneck in their ancestral population was detected (supporting the notion that founder dispersal is particularly common in seahorses^21^), however, a rapid population expansions was detected after crossing the Atlantic Ocean in the mid Miocene (Fig. 2a, b, Supplementary Fig. 10). As previously proposed^22^, ancestors of *H. hippocampus* diverged from the North American lineages likely by back-crossing the Atlantic via the Gulf Stream (a dispersal route still effective today^29^), and colonized the East Atlantic in the Pliocene.

For many marine animal taxa inhabiting the shallow areas of the Arabian Sea, the closure of the East Tethys Seaway led to an increased biodiversity^6^, as it did for seahorses, leading to a second center of biodiversity in this group. For instance, about 13 Ma the ancestors of *H. kelloggi* and *H. spinosissimus* emerged as a new lineage by dispersing back into the Indo-Australian Archipelago. This event may had been facilitated by a reinforced Equatorial Counter Current in the Indian Ocean after the closure of the Tethys Seaway^30^, and thus further contributed to the high diversity in the original center of seahorse biodiversity (Fig. 2c, d).

The South Atlantic seahorse lineage split and dispersed from the Arabian Sea southernly, along the East of the African continent. The closure of the Tethys Seaway may have enhanced the East African coast current and the Agulhas Current, which potentially assisted in this southward long-distance migration^30^. This lineage passed the Cape of Good Hope, a potentially severe dispersal bottleneck reflected in the extremely low effective population size of this lineage ~4.8–3.6 Ma, and colonized the Southern and Western African coastlines (*H. capensis* and *H. algiricus*, respectively).

**Fig. 3:**
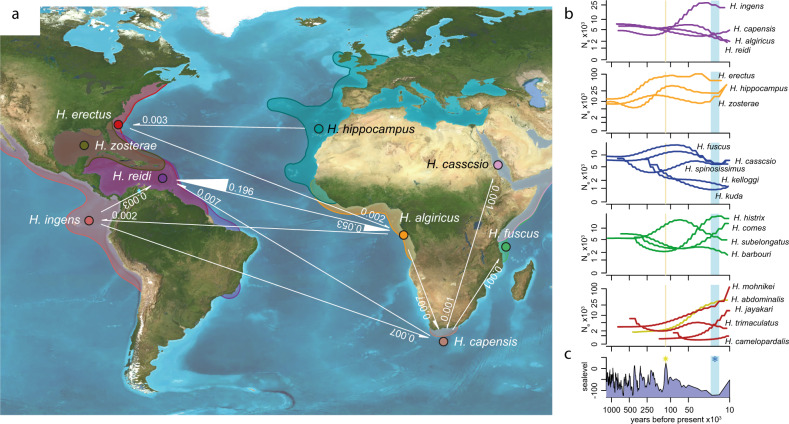
Gene flow and fluctuations in the effective population size. **a** Gene flow detected between species inhabiting the South Atlantic Ocean. Gene flow is shown nearby the white lines as migration rate deduced by G-PhoCS. Thickness and direction of the arrows correspond to rates and direction of gene flow, respectively. Maps modified from Ron Blakey © 2016 Colorado Plateau Geosystems Inc (License # 60519). Source Data are provided in Supplementary Table 10. **b** Fluctuations in effective population size by PSMC. The *x* axis represents time in years before present while the *y* axis represents the effective population size. The charts are organized mainly according to the geographic distribution for each of the species with different distribution areas. Source data are provided as a Source Data file. **c** Sea level change during the past 1 million years in meters^33^. The yellow line indicates the last global interglacial peak while the cyan shade indicates the last glacial maximum period.

Following this second invasion of the Atlantic in the early Pliocene, ancestors of the South American lineages crossed the Atlantic and colonized the South American coastlines, with *H. ingens* emerging from an early lineage that colonized the north of South America. In line with previous studies^4,21^, we also found that this lineage crossed the Panama Seaway before its final closure^4^, where it thrived as indicated by a large average effective population size (Fig. 2a, d). Subsequently, a second lineage successfully crossed the South Atlantic approximately 700k years ago and colonized the northern coast of South America and the Caribbean, from which *H. reidi* evolved. Average effective population sizes of this lineage remained relatively small, possibly as it was not able to spread into the East Pacific due to the prior closure of the Panama Seaway and the competitive disadvantage as its habitat likely overlapped with those of other seahorse species, such as *H. erectus* (Fig. 2d). Repeated crossings of the South Atlantic via rafting along the Benguela & South Equatorial Current have been proposed before^21,22^. Indeed, ongoing gene flow from the West-African *H. algiricus* into the South American *H. reidi* population with much less pronounced gene flow in the opposite direction supports the notion that rafting along these ocean currents facilitated this colonization route (Fig. 3a).

The global diversification of seahorses thus involved long-distance dispersal and has been facilitated by paleo-seaway dynamics and changing ocean currents. Specifically, our analyses finally confirm that Indo-Pacific seahorses colonized the eastern coastline of America via two distinct routes and in two waves, a topic previously under debate^19,22^: firstly, by colonizing the still open Tethys seaway and subsequent crossing of the Atlantic Ocean, and later by passing the South African Cape of Good Hope. Interestingly, the second wave occurred only in the early Pliocene, potentially facilitated by a change in the South Atlantic and Caribbean ocean current dynamics driven by the ongoing closure of the Panama Seaway^27,31^. These findings contradict a recent study that suggested only one colonization via the South Africa route^22^ and thus emphasizes the importance of a wide species representation in biogeography studies.

As outlined above, tectonic shifts and subsequent changes in ocean current dynamics likely facilitated some of the major dispersal and diversification events in seahorses, however, more short-term changes in seawater levels can also drastically affect the evolution of marine organisms inhabiting shallow water, for example by changing the amount of suitable habitat in a given area or change its structure^32^. Fluctuations in effective population sizes (*N*_*e*_*s*) were estimated back up to 1 million years ago (Fig. 3b, Figshare: Dataset 7). When such fluctuations since the last glacial peak (~120 k years ago to ~10k years ago) were compared to fluctuations in seawater levels, which are primarily driven by variations in global temperature (via glaciations)^33^, the patterns suggest a complex effect of seawater levels on *N*_*e*_ (Fig. 3c). Several seahorses’ effective population sizes appeared to be positively associated with warm climate and thus high seawater levels, as suggested by local maxima in effective population sizes following a warm period ~115 k years ago with a delay of several thousand years. These species include *H. hippocampus* (the sole European species considered), *H. casscsio*, *H. fuscus* (both lineages have restricted distribution ranges in and south of the Red Sea and East African coast), and *H. subelongatus* (only found at the West Australian coast). Effective population sizes of multiple other species show a more negative association with seawater levels with a local maximum in *N*_*e*_ coinciding with a local minimum in sea level. These species include *H. ingens* (the only species considered distributed along the Pacific side of the American continent), *H. spinosissimus*, and *H. trimaculatus*, two species broadly distributed across the Sundaic region. However, several species show no peak in *N*_*e*_ sizes likely associated with high or low seawater levels, and other factors might have a stronger influence on population sizes. For instance, species inhabiting the North Atlantic biome (*H. erectus*, *H. hippocampus* & *H. zosterae*) show generally larger *N*_*e*_ than most other lineages (e.g., those inhabiting the South Atlantic) suggesting that the biome type can affect species *N*_*e*_*s*. Furthermore, some species might be more resilient against seawater level fluctuations or glaciation induced habitat loss than others as a result of increased dispersal abilities (e.g., via rafting^7^), or because regional refugia from glaciations were available^34^.

### Convergent evolution of adaptive phenotypes

**Fig. 4:**
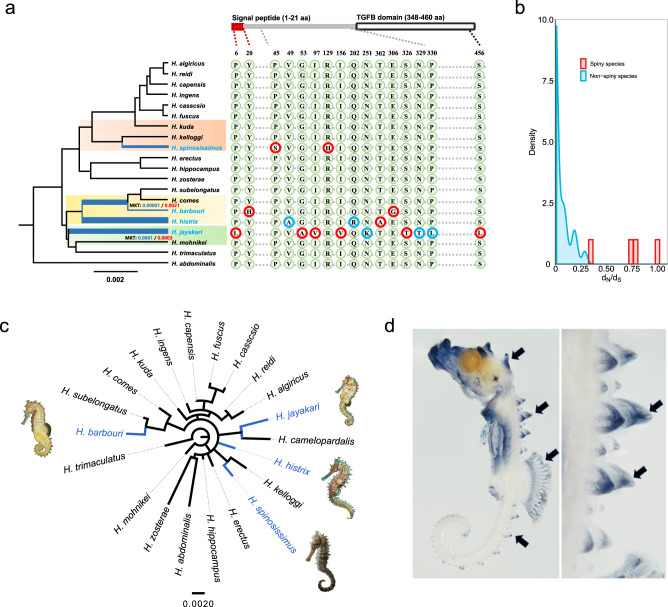
The evolution of spines. **a** Left, Species tree displaying the independent evolution of spines in seahorses. The branch length indicates number of substitutions per site. Four spiny seahorse species are highlighted in blue. Thicker branches correspond to higher rates of nonsynonymous-to-synonymous substitutions (d*N/*d*S*) for *bmp3* gene. Canonical and generalized McDonald and Kreitman test (MKT) for *bmp3* gene was performed for three pairwise sister species with divergent spiny and non-spiny features highlighted by background colors, whose significance levels were indicated by *p* value with blue and red font, respectively. Right, comparison of amino acid substitutions in bmp3 protein, polymorphic and fixed substitutions in spiny seahorses are indicated with red and blue circles, respectively. **b** Distribution of d*N*/d*S* values in *bmp3* in spiny seahorses compared to non-spiny species. **c** Independent evolution in the phylogenetic tree reconstructed for the protein encoded by *bmp3*. Seahorses illustrations by Geng Qin. **d** Whole-mount in situ hybridization of *bmp3* in *Hippocampus erectus*. In situ photos of seahorses by Ralf F. Schneider. Source data are provided as a Source Data file.

During their worldwide diversification, seahorses had to adapt to diverse combinations of abiotic and biotic factors leading to unique adaptive phenotypes^24^. Adult seahorses have only relatively few predators due to their excellent camouflage and unappetizing bony plates and spines^11^. Spines, which were derived from L-type plates covering the surface of seahorses just under the skin, are morphologically similar to the diamond-shaped dermal spines covering the skin surface in pufferfishes, which are the extreme-scale derivatives^35^. Vertebrates possess a huge diversity of skin-derived structures, including teleost fish scales, reptilian scales, avian feathers, and mammalian hair^36^. Although the skin structures are not structurally homologous, they seem to be controlled by highly conserved genetic mechanisms between the different vertebrate clades^37–39^. Previous studies have shown that Hh, Fgf, Bmp, Wnt/β-catenin, and Eda pathways were involved in teleost scale development^40–45^. It is likely that teleost skin structures (even when strongly modified), share common elements of these core signaling pathways known to underpin skin structure development throughout diverse vertebrate groups. Seahorses have also evolved variations in the degree of body coverage by spines, which may enable them to adapt to diverse ecological niches. Interestingly, species exhibiting bony spines were found to not be closely related by our species tree: *H. spinosissimus*, *H. jayakari*, *H. histrix*, and *H. barbouri*. This confirms previous findings^18^ and suggests that some lineages were exposed to similar environmental pressures, such as specific predator types, have evolved similar phenotypes independently (Fig. 4 and Supplementary Fig. 11). Spiny seahorses inhabiting the north and west Indian Ocean split from their sister lineage 8.7 and 7.8 Ma, respectively, while spiny seahorses inhabiting the Pacific Ocean diverged from their sister lineage 14.7 and 6.8 Ma (Supplementary Fig. 11).

To investigate the molecular basis of this repeatedly evolved adaptive phenotype, we performed a positive selection analysis to investigate whether accelerated nonsynonymous/synonymous mutation rate ratios (d*N*/d*S*) can be detected on the branches of spiny seahorses compared to non-spiny lineages. Using the codeml program in PAML we identified 37 genes putatively under positive selection with signals of accelerated d*N*/d*S* in spiny seahorses (*p* < 0.001, Supplementary Data 3, Figshare: Dataset 8). Protein trees obtained from the amino acid sequences of all 37 genes showed that the four spiny seahorses are not closely related to each other (Fig. 4, Figshare: Dataset 9), indicating that the spiny phenotype likely evolved independently. Specifically, the four spiny seahorse lineages exhibit independent amino acid changes in the bone morphogenetic protein 3 (*bmp3*) gene (Fig. 4a), and canonical and generalized McDonald and Kreitman tests (MKT) showed that *bmp3* evolved under positive selection (neutrality index < 1, Chi-square test *p* < 0.05) (Fig. 4a, b, Supplementary Data 4). Spines emerge in many syngnathid species’ embryos (including *H. erectus*) and are lost in some species secondarily during maturation. Although the spiny phenotype likely has a polygenic basis, whole-mount in situ hybridizations demonstrate *bmp3* expression in seahorse spines’ early developmental stages in *H. erectus*, a species whose adult stages do not have well-developed spines (Fig. 4d, Supplementary Fig. 12a). Being a transcription factor, bmp3 was shown to negatively regulate osteoblast differentiation (and thus bone mass) in mammals^46,47^, suggesting that divergent sites in this gene between spiny and non-spiny seahorses may affect its regulatory interaction with downstream genes and thus contribute to spine outgrowth in those species with derived peptide sequences. Moreover, a knockout experiment using CRISPR/Cas9 in zebrafish showed that mutants have a series of significant scale defects, such as decrements in scale numbers, rearrangements, and irregular shapes, confirming that *bmp3* plays a role in the formation of dermal bones in teleosts, and thus likely also spines (Supplementary Fig. 12b, c).

The independent evolution of complex adaptive phenotypes, such as the spine phenotype, suggests that seahorses have a generally high evolvability, in concordance with the high rates of nucleotide evolution already reported^10^ and the high diversification rates of *Hippocampus* we reported here (Supplementary Fig. 1, Figshare: Dataset 1). Thus, the ability to rapidly adapt to new environments and respond to changed selection regimes may, in addition to their unorthodox means of dispersal by rafting along oceanic currents, account for some of the evolutionary success seahorses had while diversifying globally.

In conclusion, we report that seahorses dispersed over surprisingly long distances, and diversification was assisted by changing ocean currents and tectonic events. These include two independent invasions of the Atlantic Ocean from the West Indian Ocean, one of them facilitated by the last opening of the East Tethys Seaway and the other by passing the Cape of Good Hope and, finally, the colonization of the East Pacific Ocean through the Panama seaway. Convergent evolution of adaptive traits, such as in the case of repeatedly evolved protective dermal spines suggests that developmental-genetic pathways were recruited several times independently and presumably in response to predation pressure.

## Methods

### Diversification rate estimation in the Syngnathidae

DNA sequences of 138 species of the Syngnathidae family and one outgroup were obtained from previous studies^48,49^. After sequence alignment using Clustal Omega (v1.2.4)^50^, a concatenated phylogenetic tree was obtained with RAxML (v8) using a best-scoring maximum likelihood tree search method (option -a) using a GTRGAMMA model and including 1,000 bootstrap replicates^51^. Relative divergence was estimated with the wLogDate python program^52^. Diversification rates (i.e., speciation minus extinction) were estimated using BAMM 2.5^48^. We accounted for non-random incomplete taxon sampling by including the proportion of missing taxa per genus (sample probabilities in Supplementary Data 5) as well as the overall sampled genera (=0.84). Priors were generated using setBAMMpriors in BAMMtools^48^. Analyses were run for 5 × 10^6^ generations, sampling every 1000 generations and with a 25% burn-in. DNA sequences and the estimated phylogenetic tree are available at Figshare (Dataset 1).

### Long-read sequencing and assembly of the *Hippocampus erectus* genome

A mature, male *H. erectus* bred in the aquatic farm in Fujian province, China, was used for the de novo genome assembly. Genomic DNA was extracted from tail muscles using a standard phenol/chloroform extraction protocol. Single-Molecule, Real-Time (SMRT) sequencing was performed using a total of 5 μg of genomic DNA to generate a 20 kb library according to the manufacturer’s instructions (Pacific Biosciences, USA). Subreads were obtained after size selection on a BluePippin system (Sage Science, USA). SMRT genome sequencing was performed on a PacBio Sequel platform (Pacific Biosciences, USA) to an approximate coverage of 113-fold.

Reads with the quality lower than 0.75 and length shorter than 500 bp were excluded and 6.01 M subreads comprising a total of 47.88 Gb were retained for the assembly (longest subread = 71.17 kb, average length = 7.97 kb). The draft genome was assembled using WTDBG (https://github.com/ruanjue/wtdbg). Sequence contigs were then error-corrected using Pilon^53^. Evaluation of the integrity of assembled sequences, genome size estimation, transposable element predictions and genome annotation are described in the Supplementary Information (Supplementary Methods, Supplementary Tables 2–4, Supplementary Data 1).

### High-throughput chromosome conformation capture (Hi-C) based genome scaffolding

An adult farmed male *H. erectus* was used for the Hi-C analysis. The library was prepared following a standard in situ Hi-C protocol for blood samples^54^, using DpnII (NEB, Ipswich, USA) as the restriction enzyme. A standard circularization step was carried out, followed by DNA nanoballs (DNB) preparation according to the standard protocol of the BGISEQ-500 sequencing platform^55^. The library was then sequenced with a PE100 strategy using the BGISEQ-500 platform. Quality control and library evaluation is described in the Supplementary Methods.

For Hi-C alignment and chromosome orientation, we first constructed an interaction matrix based on the valid reads. Then, the ICE software was used to correct for any preference of the enzyme-cut loci due to an uneven distribution in GC content^56^. The retrieved valid pairs (319,356,098) were then used to orientate and anchor the PacBio contigs into superscaffolds (chromosomes) applying the 3D-DNA pipeline with the key parameter of ‘-m haploid -s 4 -c 22’^57^. The contact maps were subsequently generated with the Juicer pipeline^58^, and the boundaries for each chromosome were manually rectified by visualizing the *inter.hic* file in Juicebox^59^, combining linkage information from the *agp* file.

### Re-sequencing sample preparation, mapping, and variant calling

We sampled a total of 358 seahorse specimens from 21 species representing the major lineages of the genus *Hippocampus* (Fig. 1a, Supplementary Data 2), including 13 to 22 individuals per species, except for *H. cassisio*, *H. capensis*, and *H. camelopardalis*, represented by 8, 7, and 2 individuals, respectively. The classification of each specimen was based on morphological and genetic evidence^16^. Genomic DNA was extracted from tail muscles using a standard phenol/chloroform extraction method and used to construct an approximately 350 bp-insert-size sequencing library. Paired-end libraries were sequenced on an Illumina HiSeq 4000 platform. One random sample for each species was sequenced at ~20-fold coverage, and the rest were sequenced at ~10-fold coverage.

After the removal of adapters and low-quality reads (Supplementary Methods), clean reads for each individual were mapped to both the PacBio genome sequence of *H. erectus* and the Illumina genome sequence of *H. comes* using BWA-MEM with default parameters (v0.7.17)^60^. We calculated mapping rates, depth, and genome coverage using SAMtools (v1.6) after sorting and removal of duplicates^61^. The assembled *Hippocampus erectus* PacBio genome was then used as the reference genome.

By assigning 21 species, we then performed variant calling for all 358 individuals using FreeBayes v9.9.2^62^. Mapping and base quality filters were used as default in FreeBayes (–standard-filters flag). Details are shown in Supplementary Methods. The filtered dataset was then annotated according to the *H. erectus* genome using the package ANNOVAR^63^.

### Analysis of genetic diversity and divergence

Inter-species genomic divergence was calculated for each pair of the 21 seahorse species, using the specimen with the highest sequencing fold coverage per species. We also calculated pairwise genetic distances among all 358 specimens using PLINK (v1.9) with the main parameter ‘–distance 1-ibs flat-missing’^64^. A neighbor-joining (NJ) tree was then constructed using MEGA7^65^. Principal component analyses (PCA) were performed using smartPCA program within EIGENSOFT (v6.1.4)^66^.

We furthermore analyzed intra-specific nucleotide diversity using ANGSD (v 0.924)^67^ using sliding-window approach as stated in Supplementary Methods. Both Watterson (*θw*)^68^ and pairwise (*θπ*)^69^ estimators of theta were used for nucleotide diversity analysis (Figshare: Dataset 2). R packages ‘vioplot’^70^ and ‘circlize’^71^ were employed to explore nucleotide diversity among the different species and chromosomes.

### Global colonization patterns

For our phylogenetic analyses, first gene families for *Syngnathus scovelli*, *H. erectus*, and *H. comes* were identified using Treefam^72^. After filtering low-quality genes with a premature termination codon or in which the base number of the coding region was not a multiple of three, gene family analyses were carried out and identified 5,475 single-copy orthologs^10^. Pair-wise alignments for *H. erectus* and *S. scovelli* were conducted using prank v.140603^73^ and CDS sequences for 2,000 orthologs (randomly selected from the above mentioned) were then extracted for each specimen based on the SNP dataset (Figshare: Dataset 3).

A coalescent-based phylogenetic tree was constructed using ASTRAL-III v5.6.1^74,75^, with a total of 2,000 independent gene trees and including one to five specimens for each of the species (103 specimens in total). Loci selected have an average length of 1,548 (± 1,325), average segregating sites of 18% (± 4%) and average missing data of 1%. Gene trees were generated using RAxML (v8) using the rapid bootstrap analysis and searched for the best-scoring maximum likelihood tree (option a) under a GTR + G substitution model and including 100 bootstrap replicates^51^. The DNA matrices, gene trees, and ASTRAL inference are available at Figshare (Dataset 4).

To obtain divergence time estimates of the nodes in the *Hippocampus* species tree, 100 loci were randomly subsampled for the same one to five individuals per species (from the above list; 103 individuals in total), using the package starBEAST2 implemented in BEAST v2.4^76^. Loci selected for this analysis had an average of 1,579 bp (± 1,060), average segregating sites of 18% (± 4%) and 1% missing data. For calibration points, we used data from the paleontological work of *Hippocampus*^77^ and other related groups of pygmy pipehorses and pipehorses^77–79^. Thus, using a lognormal distribution as hyperprior, we first calibrated the origin of *Hippocampus* genus to the youngest possible age of 11.6 Ma for which *Hippocampus* fossils were recorded as well as the existence of pipehorses and pygmy pipehorses has been shown^77–79^ (Supplementary Table 9). Thus, this prior assumes that *Hippocampus* genus originated before the occurrence of the oldest known fossil of *Hippocampus* (*H. samarticus*)^77^, and we also relax a wide 95% HPD interval to accommodate uncertainty (95% HPD: 14.4-31.8). Second, we incorporated the information of the *H. sarmaticus* fossil from the Miocene as an ancestor of *H. trimaculatus* using a lognormal distribution with a mean 11.8 Ma to lead the median close to 11.6 Ma and the standard deviation was set to model uncertainty, covering the complete Middle Miocene upper bound to the Late Miocene (95% HPD: 8.32-16.1 Ma)^77^. Finally, following Teske and Beheregaray^17^, we set the divergence between *H. reidi* and *H. ingens* to a minimum of 2.8 Ma, in correspondence to the last connection between the Caribbean and the Pacific Ocean^80^. Although it has been argued that Colombian sediments supported the existence of Miocene temporal closures of the Panama seaway^80,81^, for this study we used a conservative prior by setting the minimal possible divergence time between these lineages to 2.8 Ma and also allowed the hyperprior to cover older dates, with 95% HPD: 3.07-4.64 Ma, given that O’Dea et al. suggested that a connection between the Atlantic and Pacific Oceans allowing gene flow likely existed until 3.2 Ma (gradually reduced in time)^80^. All remaining settings were used as default, including unlinked strict clocks and unlinked JC69 substitution models among loci. We fixed the ASTRAL tree topology and ran two independent analyses during the 160 ×10^8^ steps of the MCMC chain and sampled at every 80,000 generations. Convergence was diagnosed using Tracer v1.7^82^. The two independent runs were combined using LogCombiner (included in BEAST v2.4 package) with a 10% burn-in. The maximum credibility tree was obtained using TreeAnnotator (also included in BEAST v2.4 package). The DNA matrices and BEAST xml input file and outputs are available at Figshare (Dataset 5).

The topology and branch lengths (divergence times) of the species tree were used to reconstruct the geographic diversification under two different models of diversification in space: diffusion^83,84^ and heterogeneous landscape^85^. Both models were run in BEAST v2.4, using a lognormal clock and tip coordinates matching the sampling points and current distribution of the species. For the heterogeneous model, we included a deformation in the continental areas by increasing the friction in an external kml file, to decrease the probability of migration through continents and nearby seaways. We ran different values of friction and deformation, including deformation = 10, 20, 50, and 100 and valued each polygon at 2 (higher deformation, higher friction). Due to the high similarity in the results, we only presented the results with deformation = 20; value = 2 (Supplementary Fig. 9a). Convergence was diagnosed using Tracer, and Tree Annotator was used to export the final tree with a 10% burn-in. Finally, we used SPREAD (v1.0.6)^83^ to generate a kml file and Google Earth Pro to plot and animate the diversification of the *Hippocampus* genus in space and time. The BEAST xml input file and outputs are available at Figshare (Dataset 6).

### Demographic inference with G-PhoCS

A total of 102 representative specimens (2-5 specimens for each species) were used to infer the demographic history of seahorses. Neutral loci were used to run the demographic analysis^86^. The filtering strategy is summarized in Supplementary Methods.

52.2% of the genome remained after filtering, from which we selected 6102 ‘neutral loci’ by identifying contiguous intervals of 1 kb that passed the filters. We used the default settings chosen by Gronau *et al*.^86^: a Gamma distribution (α = 1.0, β = 10,000) for the mutation-scaled population sizes (θ) and divergence times (τ), and a Gamma (α = 0.002, β = 0.00001) prior for the mutation-scaled migration rates (*m*). The Markov Chains exploring the space of parameter values were run for 100,000 burn-in iterations with an additional 200,000 iterations. The mean sampled value and the 95% Bayesian credible interval of each parameter were calculated by Tracer v1.7.1^82^. We assumed an average mutation rate (*μ*) of 4.33 × 10^−10^ per nucleotide per generation^10^ and an average generation time of one year for the *Hippocampus* species. The population size estimates (*Ne*) were obtained from the mutation-scaled samples (*θ*) based on the formula *Ne* = *θ* / 4*μ*. Gene flow was measured by the total migration rate, which is the per-generation rate times the number of generations in which migration was allowed (Fig. 3a, Supplementary Table 10).

### Inference of demographic history from PSMC analysis

Pairwise sequentially Markovian coalescence analyses (PSMC)^87^ were used for one individual (with the highest genome coverage) per species for interspecific comparisons. Genotype information of the selected individual was retrieved from the alignment BAM files using SAMTOOLS^61^. Variants with sequencing depth less than a third of the average depth or greater than 2.5 times were removed. The program fq2psmcfa was used to convert the diploid consensus sequence to a FASTA-like format where the characters indicated heterozygous positions in consecutive bins of 100 bp. The program psmc was then used to infer the population size history^87^, where the parameters were set as -N 30 -t 15 -r 5 -p 4 + 25*2 + 4 + 6. We assumed a generation time of 1 year and a mutation rate (*μ*) of 4.33×10^−10^ per nucleotide per generation^10^.

### The genetic basis for the spine trait

Four seahorse species used in this study, including *H. spinosissimus*, *H. jayakari*, *H. histrix*, and *H. barbouri*, typically show well developed spines^16^ (Fig. 3a, Supplementary Fig. 11). To detect positively selected genes (PSGs) potentially related to bony spines, we reconstructed gene sequences for 20 seahorse species (excluding *H. camelopardalis* with extremely low sequencing depth) using both SNPs and invariant sites (*H. erectus* genome as reference). The aligned codon sequences for each gene were further analyzed using codeml program in PAML^88^ to calculate the d*N*/d*S* and we detected positive selection on particular branches considering the phylogenetic relationships among these 20 species (obtained using ASTRAL; described in Phylogenetic analysis Section). The ‘one-ratio’ and ‘two-ratio’ codon substitution models were considered. ‘One-ratio’ model assumes the same d*N*/d*S* across all the branches in the phylogeny of species, which was termed as the ‘null hypothesis’. The ‘Two-ratio’ model presumes diverged d*N*/d*S* for the branches of spiny and non-spiny lineages, as ‘alternative hypothesis’. Likelihood ratio tests were conducted to compare the above-mentioned models by calculating the corresponding likelihoods, χ^2^ critical values, and *p* values for each gene. We adopted a relatively strict threshold of 0.001 for the original *p* values to initially obtain a set of 37 putative genes under positive selection with significantly accelerated d*N*/d*S* on the branches of spiny seahorse lineages (Supplementary Data 3).

To further characterize the functional genes potentially relevant to spine development for the 37 candidate genes, we performed canonical and generalized MKT to detect the signature of natural selection based on population genomic sequences. For the canonical MKT, the number of nonsynonymous (d*N*) and synonymous (d*S*) variants between three pairwise sister species with divergent spiny and non-spiny features, containing *H. spinosissimus* and *H. kelloggi*, *H. jayakari* and *H. mohnikei*, and *H. barbouri* and *H. comes*, and those nonsynonymous (Pn) and synonymous (Ps) variants within species were estimated, where *H. kuda* and *H. kuda* & *H. histrix* were considered as outgroups, respectively. According to these tests, the neutrality index (NI = (Pn/Ps)/(d*N*/d*S*)) were calculated, and a Chi-square test was implemented. NI < 1 indicated high divergence between species due to positive selection. We performed generalized MKTs, where, d*N* and d*S*, were estimated as the derived nonsynonymous and synonymous variations for one of the sister species with divergent spine status contrasted with the ancestral and sister species, which were then compared to the Pn and Ps, putatively neutral, in this lineage.

We also implemented the ‘Free-ratio model’ to estimate the variable d*N*/d*S* ratio on each phylogenetic branch based on the aligned codon sequences for each of the 37 genes through the maximum likelihood method using CODEML in PAML^88^. Distribution of d*N*/d*S* values of the 37 putative PSGs in 20 seahorse species are available at Figshare (Dataset 8). By integrating the results from abovementioned analyses, the genes simultaneously showing significance in PAML and MKT, and consistently presenting accelerated d*N*/d*S* from ‘Free-ratio model’ on the branches of spiny seahorse lineages in comparison with those of non-spiny lineages, especially with those of sister non-spiny lineages, were considered as confident candidates for further experimental confirmation.

Additionally, we reconstructed the CDS sequences of 37 PSGs for 21 seahorse species (one specimen with the highest sequence fold coverage for each species) with the filtered SNP dataset and translated them into protein sequences using in-house scripts. We estimated the protein trees using RAxML (v8)^51^ using the rapid bootstrap analysis and search of best-scoring maximum likelihood tree (option a) under a PROTGAMMAGTR substitution model and including 100 bootstrap replicates. The protein trees of these 37 PSGs from 21 seahorse species are deposited at Figshare (Dataset 9).

Multiple sequence alignment analysis was then performed for *bmp3* based on the generated protein sequences. Only private amino acid substitutions that were polymorphic or fixed in spiny seahorses were retrieved. Private, polymorphic substitutions refer to amino acid substitutions that were segregating exclusively in one or more of the four spiny seahorses, while private, fixed substations refer to amino acid substitutions that were fixed exclusively in one or more of the four spiny seahorses.

Whole-mount in situ hybridization of *bmp3* was performed with embryos of the lined seahorse *H. erectus* at different developmental stages, including approximately four, three, two, and one day prior to birth (for the latter, three independent replicates were performed with coinciding expression patterns). Embryos were dissected in RNase-free 1X phosphate-buffered saline and fixed in 4% paraformaldehyde (PFA) at 4 °C overnight. For *bmp3*, a specific antisense RNA probe was synthesized^87^: digoxigenin-labeled UTPs (Roche, item-nr. 11277073910) and SP6 RNA Polymerase (Roche, item-nr. RPOLSP6-RO) were used to synthesize antisense RNA probes from plasmids in which a *bmp3* PCR fragment was cloned behind a SP6 RNA Polymerase promoter (Supplementary Table 11). Hybridization procedures mostly followed previously described protocols^89^: firstly, embryos were bleached and cleared in 1.5% H_2_O_2_ in 1% KOH until pigmentation was removed (was only done for the sample presented in Fig. 4), then permeabilized using 10 µg/ml proteinase K in Tris-buffered saline with 0.1% Tween-20 (TBS-T) for 15-20 min, then endogenous alkaline phosphatase (AP) activity was deactivated using a solution of 0.2 M triethanolamine (pH 7.5) with 2.5% acetic anhydride added directly before treatment (for 20 min), and a refixation using 4% PFA for 20 min was performed. In between steps, washes were performed with TBS-T. Subsequently, samples were equilibrated with the hybridization mix at 68 °C for 4 h, followed by overnight hybridization using hybridization mix with 100 ng probe/ml at 68 °C. Samples were then repeatedly washed using a mix from 5x saline sodium citrate (SSC), 50% formamide and 2%Tween-20 at 68 °C, followed by washes in 2x SSC with 0.2% Tween-20 at room temperature. After washes with TBS-T, samples were blocked using blocking buffer for 1.5 h, and then treated with the anti-DIG-AP antibody (1:4000 concentration; Roche, item-nr. 11093274910) in blocking buffer for 5 h at room temperature. After repeated washing of samples for 2 days with maleic acid buffer, they were kept in AP buffer (with Levamisol) for 20 min, after which they were moved to BM-Purple until desired color intensity was reached (Roche, item-nr. 11442074001), and finally photographed.

To investigate the phenotypic consequences of *bmp3* loss in a teleost, we used a CRISPR/Cas9 strategy to generate a *bmp3* mutant zebrafish line according to Miguel et al.^90^. The *bmp3* guide RNA (gRNA) was designed online (https://www.crisprscan.org/?page=gene) targeting the first exon of zebrafish *bmp3*. The gRNAs was constructed by overlapping PCR. This method requires a target-specific DNA oligo (top-strand oligo) and a generic DNA oligo for the guide RNA (Supplementary Table 11). The target-specific oligo contains a T7 promoter, the target sequence and finally a 20-nt sequence complementary to the guide RNA (Supplementary Table 11). The two oligos are annealed and extended with DNA polymerase, and the resulting product serves as a template for in vitro transcription using the mMESSAGE mMACHINE™ T7 Transcription Kit (Thermo Fischer Scientific AM1344) and the transcripted production was purified using the RNA Clean & Concentrator™-5 (Zymo Research R1014). The pT3TS-nCas9n vector was synthesized using the XbaI restriction enzyme (NEB R0145S) and performed in vitro transcription and purification using mMESSAGE mMACHINE™ T3 Transcription Kit (Thermo Fischer Scientific AM1348).

The transgenic zebrafish parent labeled with green fluorescent protein for osteoblast-specific transcription factor (Osterix GFP) used in this experiment were cultured at 26–28 °C under a controlled light cycle (14 h light, 10 h dark) to induce spawning. Purified sgRNAs (80 ng/μl) were co-injected with Cas9 mRNA (400 ng/μl) into zebrafish embryos at the one-cell stage. These founders (F0) fish were raised to maturity and the genotyping primers (Supplementary Table 11) were used to screen out F0 with site mutations by the fin clipping, DNA extraction, PCR spanning the target site and sequencing. The adult F0 with mutation were outcrossed with wild-type fish to obtain F1 fish, which were subsequently genotyped. The F1 fishes with the same mutant genotype transmitting a frameshift mutation were inbred to obtain homozygous F2 fish, which were used for further phenotypic observation. Osterix GFP-labeled mutant and wild specimens were observed and photographed under a Leica M205 FA Fluorescent Stereo Microscope (Wetzlar, Germany). All experiments were performed in accordance with approved Institutional Animal Care and Use Committee protocols of the scientific ethic committee of the Huazhong Agricultural University (HZAUFI-2018-018).

As results, we didn’t observe allele mutation for dre-bmp3-gRNA1, so no stable line was generated for this CRISPR. But for dre-bmp3-gRNA2, two *bmp3* nonsense alleles with 14 bp insertion (*bmp3*^+14^) and 2 bp deletion (*bmp3*^−2^) in the first exon were generated (Supplementary Fig. 12b), which both caused frame-shift mutations at the 69th AA, and premature transcription termination event at the 161th and 94th AA, respectively. In the F2 mutant *bmp3* fish, we observed a series of scale defects, such as decrements in scale numbers, rearrangements, and irregular shapes. The F2 *bmp3*^+14^ mutant fishes gave 4/29 fish with scale defects whereas 3/31 had scale defects for F2 *bmp3*^−2^ mutant fish.

### Reporting summary

Further information on research design is available in the Nature Research Reporting Summary linked to this article.

## Supplementary information

Supplementary Information

Peer Review File

Reporting Summary

Descriptions of Additional Supplementary Files

Supplementary Data 1

Supplementary Data 2

Supplementary Data 3

Supplementary Data 4

Supplementary Data 5

## Data Availability

All sequencing data generated in this project are available at NCBI under BioProjects PRJNA613175 (PacBio, https://www.ncbi.nlm.nih.gov/bioproject/PRJNA613175), PRJNA613176 (Hi-C, https://www.ncbi.nlm.nih.gov/bioproject/?term=PRJNA613176) and PRJNA612146 (Re-sequencing, https://www.ncbi.nlm.nih.gov/bioproject/?term=PRJNA612146). In addition, processed datasets including custom codes (Datasets 1–9) are available at Figshare (https://figshare.com/articles/dataset/Genomics_reveals_seahorses_global_dispersal_routes_and_elucidates_the_genetics_underlying_a_convergent_adaptive_trait/13568186). Source data are provided with this paper.

## Source data

Source Data

## Code availability

Custom scripts employed for the analysis of the sequencing data are available at Figshare (https://figshare.com/articles/dataset/Genomics_reveals_seahorses_global_dispersal_routes_and_elucidates_the_genetics_underlying_a_convergent_adaptive_trait/13568186).
