# Supplementary material for: Genome sequences reveal global dispersal routes and suggest convergent genetic adaptations in seahorse evolution
Source: Nat Commun. 2021 Feb 17;12:1094. doi: 10.1038/s41467-021-21379-x (PMC7889852; doi:10.1038/s41467-021-21379-x)
Supplement: Supplementary file 1 — Supplementary Information [file 41467_2021_21379_MOESM1_ESM.docx]

Supplementary Information

The PDF file includes:

Supplementary Methods

1. Long-read sequencing and assembly of the *Hippocampus erectus* genome

1.1. Integrity of the assembled sequences

1.2. Genome size estimation

1.3. Transposable element prediction

1.4. Gene prediction and annotation

2. High-throughput chromosome conformation capture (Hi-C) based genome scaffolding

2.1. Karyotype analysis

2.2. Quality control and library evaluation

3. Reads mapping and variant calling

4. Analysis of genetic diversity

5. Filtering criteria for neutral loci used for G-PhoCS analysis

Supplementary Text

1. Seahorse colonization and speciation routes are linked to prevalent oceanic surface currents and were affected by the tectonic events altering the currents

1.1. Late Oligocene to early Miocene: the origin of seahorses and early diversification

1.2. Mid-Miocene: major seahorse lineages arise

1.3. Late Miocene to early Pliocene: the second period of seahorse diversification

Supplementary Figures (1-12)

Supplementary Tables (1-11)

Supplementary References (1-41)

# Supplementary Methods

1. Long-read sequencing and assembly of the *Hippocampus erectus* genome

1.1. Integrity of the assembled sequences

CEGMA (v2.5)^1^ and BUSCO v2^2^ were employed to evaluate the integrity of the assembled sequence. The CEGMA analysis found 453 out of 458 (98.91%) core genes in the *H. erectus* genome with > 70% identity, and the BUSCO analysis identified 274 out of 303 (90.43%) highly conserved genes

1.2. Genome size estimation

Sequencing datasets of 220 bp and 500 bp short-insert libraries for the lined seahorse *H. erectus* were downloaded from NCBI (PRJNA347499). To evaluate the genome size^3^, a *K-mer* spectrum analysis was performed as described below:

Genome size = *K_num_ / K_depth_;*

where *K_num_* is the number of *K*-mers, and *K_depth_* is the expected depth of *K*-mers. The two short-insert sequencing datasets were combined to generate a 27.95 Gb dataset with an average read depth of 67.41x (fold coverage). After filtering, we retained a total of 21,128,121,729 effective *K*-mers with an average depth of 57x based on 19-mers. The estimated genome size for *H. erectus* was 414.57 Mb. Based on the 19-mer results, the proportion of repetitive elements and level of heterozygosity were estimated to be 18.63% and 0.56% of the genome sequence, respectively. In addition, we used a range of different *K*-mers to evaluate the genome size of *H. erectu*s, all of which resulted in similar estimates to the 19-mer analysis (Supplementary Table 1).

1.3. Transposable element prediction

Based on the homology-based approach and a *de novo* approach, we constructed a transposable element (TE) database for the *H. erectus* genome using LTR_FINDER^4^, PILER-DF^5^, and RepeatScout^6^. PASTEClassifier^7^ was employed for database classification and the resulting data was then merged with the Repbase^8^ database to yield the final TE database. After RepeatMasker^9^ software prediction, we obtained 116 Mb of TEs (without overlap), covering 27.58% of the genome (Supplementary Data 1). Of the 52 identified TE types, four catalogs account for 89.44% of the total predicted TEs. The richest TE catalogs of DNA transposons were TIR/Tc1-Mariner (45.01 Mb, 10.7%) and TIR/hAT (18.37 Mb, 4.37%); while most rich retrotransposons were LARD (19.65 Mb, 4.67%) and LINE/Jockey (16.11 Mb, 3.83%). These results were consistent with a previously reported study that hAT and Tcl/Mariner type of DNA transposons are the main forms for the expansion of bony fish TEs^10^.

1.4. Gene prediction and annotation

Based on the repeat-masked genome, we combined three different strategies for the prediction of gene models in *H. erectus*:

1. *De novo* prediction was performed using Augustus^11^, GlimmerHMM^12^, and SNAP^13^.
2. Based on the five reported genome datasets of *Gasterosteus aculeatus*, *Xiphophorus maculatus*, *Danio rerio*, *H. comes*, and *H. erectus* (genome based on Illumina short-reads), homology-based gene model prediction was conducted with GeMoMa^14^.
3. Based on the data from transcriptome analysis of *H. erectus*^15^, Decoder ([http://transdecoder.github.io](http://transdecoder.github.io/)), GeneMarkS-T^16^, and PASA^17^ were used to refine gene models.

We then combined the results from these three methods with EvidenceModeler^18^, to obtain a total of 20,137 genes (Supplementary Table 3). The average gene length was 12.42 Kb and the average exon length was 260 bp. We further annotated the gene models using TrEMBL^19^, NR^20^, GO^21^, KOG^22^, and KEGG^23^ databases. In total, 96.02% of the predicted genes were annotated using the different databases listed above (Supplementary Table 4).

2. High-throughput chromosome conformation capture (Hi-C) based genome scaffolding

2.1 Karyotype analysis

For karyotype analysis, a total of 20 specimens consisting of 10 males and 10 females of *H. erectus* were used. For conventional karyotyping, chromosome preparations were made following an air-drying method^24^, and stained with a 5% Giemsa solution (pH 6.8). Slides were then de-stained according to the controlled silver nitrate one-step method for the characterization of the nucleolus organizer regions^25^. Based on this analysis, the modal diploid number of 44 chromosomes was established for each of the *H. erectus* specimens (Supplementary Fig. 3).

2.2 Quality control and library evaluation

Library quality control mainly included unique read-pair mapping, valid enzyme-cut fragment detection, and duplicate reads removal according to the HiC-Pro pipeline^26^. A total of 142.99 Gb (714,934,685 pairs of reads) of raw data were generated, and we obtained a total of 380,918,232 pairs of valid reads with an effective rate of 53.28%. The valid reads were retrieved using the *H. erectus* PacBio assembly as the reference genome.

3 Reads mapping and variant calling

Low-quality reads (more than 10% of the read bases are unidentified nucleotides or Phred quality score of more than 50% of the read bases are less than 20 or more than 10 nucleotides of the read bases are overlapped with the adapter sequences) were filtered. In total, we obtained 2.57 Tb clean data (average = 47.3 M clean reads per specimen) (Supplementary Table 6).

For variant calling and genotyping, the *H. erectus* PacBio genome was subdivided into 5-Mb segments by in-house perl scripts and analyzed in parallel. Only biallelic variants with a minimum quality score (QUAL) of 30 were used for further analyses. We filtered against strand bias using the command ‘SAF>0 & SAR>0’ within vcffilter and then analyzed the alternative allele depth for each specimen. Variants with less than two reads supporting the alternative allele were removed (‘AO>1’ command) and only variants with a total depth of more than one fifth but less than five times the peak depth of the species were retained for analyses. We summarized the total number of segregating sites independent of the reference for each species using the criteria ‘minor allele count >=1’. In total, 41.79 M variants were called (Supplementary Table 7), of which 31.46 M (75.28%) were single-nucleotide polymorphism (SNPs) within species, which means these SNPs are polymorphic in at least one species (Supplementary Table 8).

4 Analysis of genetic diversity

Intra-specific nucleotide diversity was calculated using ANGSD (v 0.924)^27^ with a sliding-window approach (50-kb windows sliding in 10-kb steps). Primary parameters were: angsd -doSaf 1 -minMapQ 30 -minQ 20 -minInd 10 -minIndDepth 5 -GL 1; realSFS; angsd -doThetas 1 -doSaf 1 -pest -GL 1; thetaStat. For *H. casscsio*, *H. capensis*, and *H. camelopardalis*, the -minIndDepth parameter was set to 5, 4, and 2, respectively, due to their lower sample size.

The LD between any pair of variants within a distance of 20 kb was measured by *r^2^*, the square of the correlation coefficient of allele frequency between the two variations, calculated using Haploview^28^. The variants with minor allele frequencies below 0.05 and that significantly deviated from Hardy-Weinberg equilibrium (*p* < 0.0001) were excluded in the calculation for each species. Apart from *H. capensis* (*n* = 7) and *H. casscsio* (*n* = 8), ten randomly selected individuals for each of the seahorse species were used for LD analysis (Supplementary Fig. 7).

5 Filtering criteria for neutral loci used for G-PhoCS analysis

Neutral loci were used to run the demographic analysis. The strategy used to filter the neutral loci is summarized as follows:

1) Clustered SNVs. The variant sites that are within 15 bp of the other variants were masked.

2) Simple repeats. Regions annotated as simple repeats by Tandem Repeats Finder (TRF) were masked.

3) Transposable elements. Regions annotated as transposable elements by RepeatMasker were masked.

4) Excess depth of coverage. The sites whose depth of coverage was more than twice the mean depth of coverage for each of the species were excluded.

5) CpGs. Positions containing hypermutable CpG dinucleotides were excluded.

6) Exons of protein-coding genes. Genomic regions overlapping exons of protein-coding genes (including UTRs) were eliminated.

7) Non-coding RNAs. Genomic regions overlapping exons of non-coding RNA genes were eliminated.

# Supplementary Text

1. Seahorse colonization and speciation routes are linked to prevalent oceanic surface currents and were affected by the tectonic events altering the currents

1.1. Late Oligocene to early Miocene: the origin of seahorses and early diversification

In line with previous studies^29,30^, our analyses suggested that the common ancestor of sampled seahorses evolved ~20-25 Ma in the West Pacific ocean (today's South China Sea) (Fig. 2a and Supplementary Fig. 9). Specifically, we found that their evolutionary origin may have been situated to the west of the Sundaic region (the region around today’s Malaysia and Indonesia) (Fig. 2b). At this time, the Sunda shelf formed a closed landmass that split and diverted water delivered by the Pacific’s North Equatorial Current into a northeastern directed current (along today’s Chinese Coast), and in a southwestern direction. Here, Wallacean landmasses had only started to emerge and the surface currents still transported substantial amounts of water along the North-West Coast of the Australian continent into the Indian Ocean^31,32^. Oceanic surface currents thus likely facilitated seahorse dispersal in these directions by rafting, which likely established this area as a center for early seahorse diversification^33^. About 23.1 Ma, seahorses spread south-eastwards towards Australia, forming a lineage represented today only by *H. abdominalis* in our dataset. Its sister lineage diverged again approximately ~18.2 Ma and one descending lineage moved westwards, passing the southern tip of the Sundaic landmass and eventually colonized the shallow waters of East-African shores (Fig. 2b and Supplementary Fig. 9). This very far colonization leap into the west may have again be enabled by the seahorses’ rafting abilities: southwestwards surface currents passing through the Indonesian Seaway fed into the South Equatorial Current of the Indian Ocean, which transports surface water directly towards East Africa^33^. This lineage’s sister lineage in the South China Sea underwent divergence approx. 17.4 Ma into two clades (one containing *H. comes*, *H. subelongatus*, *H. barbouri*, and *H. histrix*, and a second containing *H. camelopardalis, H. jayakari, H. mohnkei* and *H. trimaculatus*). The second clade produced another lineage (which included *H. camelopardalis* and *H. jayakari*) colonizing East African waters approximately 17.1 Ma, i.e. prior to the closure of the Indonesian Seaway in the Late Miocene/Early Pliocene period^34^ (Fig. 2b and Supplementary Fig. 9).

1.2. Mid-Miocene: major seahorse lineages arise

In contrast to previous studies, our analyses suggest that descendants of the first seahorse lineage colonizing East African waters followed northward surface currents along the East African coast^33^ and colonized the Tethys Sea (Fig. 2c and Supplementary Fig. 9). During the early and mid-Miocene, the Tethys was a shallow sea connected to the Atlantic Ocean in the west (via the Gibraltar Seaway) and to the Indian Ocean in the south-east, as it spread over large parts of southern Europe as well as south-western Asia^34^. Based on our estimates of the effective population size, a relatively small ancestral population of Tethyan seahorses diverged approximately 15.2 Ma from the Indian Ocean source population, which predates the initial closure of the East Tethys seaway due to tectonic shifts by approximately 15 Ma, the so-called Tethyan Terminal Event (TTE)^35^. The Tethyan seahorse lineage spread through the Tethys into the East Atlantic approximately 13.3 Ma. Individuals crossed the North Atlantic and likely colonized the coastlines of the Caribbean islands and the North American mainland, most likely by rafting. The surprisingly small population size of this lineage suggests a population bottleneck prior to further diversification, indicating that only a small population colonized the Tethys or that only a small founder population dispersed to North America (Fig. 2c and Supplementary Fig. 10). This colonization pattern also suggests that seahorse fossils found in Slovenia, dating approximately 12-13 Ma back in time, did not belong to the same lineage^36^. Firstly, the morphological evidence suggests that these are more similar to either dwarf-seahorses (which are more basal seahorses not considered in this study) or related to *H. trimaculatus*. Secondly, these seahorses would have lived in the Paratethys. While it is possible that the Paratethys maintained marine conditions after the TTE until approximately 12-13 Ma, when a temporary connection to the Indian Ocean was re-established, it seems more plausible that the Slovenian seahorse lineage colonized the Paratethys independently via this seaway. Our results are thus conflicting with the previous findings suggesting colonization of the Mediterranean Sea by this lineage via the Gibraltar Seaway after passing the Cape of Good Hope and colonization of West-African waters^37^. Additionally, our phylogenetic analysis contradicts the hypothesis that a clade containing *H. erectus, H. hippocampus,* *H. zosterae*, *H. ingens, H. reidi,* and allies, first colonized South America, then spread to North America and finally colonized Europe^37^, which is also rendered unlikely by the strong south-wards surface currents along the north shores of South America until the Late Miocene/Early Pliocene^33^. The East Indian/West Pacific Ocean sister lineage of these first African colonizers also underwent repeated divergence, with one descending lineage (containing *H. jayakari* and *H. camelopardalis*) also colonizing the East African shores.

1.3. Late Miocene to early Pliocene: the second period of seahorse diversification

After a period of four million years (~13-9 Ma), in which no further divergence events were detected, East African lineages continued to diversify and one lineage passed the South African tip (~ 4.8 Ma), colonizing West African shores. Shortly after, South American shores were colonized by this lineage, likely via rafting as the Late Miocene/Early Pliocene Benguela current transported surface water from West Africa (including rafting seahorses) almost directly northwest-wards towards South America. While during the Miocene, southeastwards current along the northeastern coast of South America likely impeded northwards dispersal via rafting, the direction of this current reversed in the late Miocene and Pliocene due to the closure of the Panama seaway^38^. Seahorses rafting from West Africa or dispersing seahorses from more southern areas along the South American coast had a direct surface current that could transport them into Caribbean waters. Our analysis suggests that the ancestors of *H. ingens* diverged from other West-African seahorses 3.6 Ma, potentially using the Benguela current as a means of transportation via rafting. The ancestors of *H. reidi* crossed the Atlantic then approximately 700,000 years ago after the Panama seaway closed. In fact, we find evidence of pronounced gene flow from the West-African *H. algiricus* into *H. reidi*, consistent with the oceanic currents, and suggests that individuals of this species have kept crossing the Atlantic Ocean westward and continued to contribute to the gene pool of *H. reidi* (Fig. 3a). Our results confirmed the previous speculation of both “a West-Pacific origin”^30,39^ and “two invasions of the Atlantic Ocean”^29^ with evidence for gene flow and the migration route based on phylogeny, geographic coordinates, and divergence time analysis.

For the North Atlantic seahorses, our analysis could not resolve whether *H. erectus* split from *H. zosterae* before (i.e. due to) the colonization of East American shores, or after, and accordingly it is not entirely clear whether *H. hippocampus* diverged from *H. erectus* because *H. erectus*’ ancestors moved from Europe to North America, or because *H. hippocampus*’s ancestors moved from North America to Europe. Considering that the Gulf Stream appears as a more suitable driver for dispersal by rafting than the Atlantic Ocean’s north equatorial current^40^, the latter scenario appears more likely. At the same time seahorses’ lineages inhabiting the shores of the Sundaic region diversified, probably driven by decreasing sea-levels and subsequent island formation, leading to the lineages that are reflected by *H. comes*, *H. barbouri*, and *H. subelongatus* in our study. Interestingly, some gene flow from *H. comes* to *H. subelongatus* was detected, but not vice versa (Supplementary Table 10).

Supplementary Figures


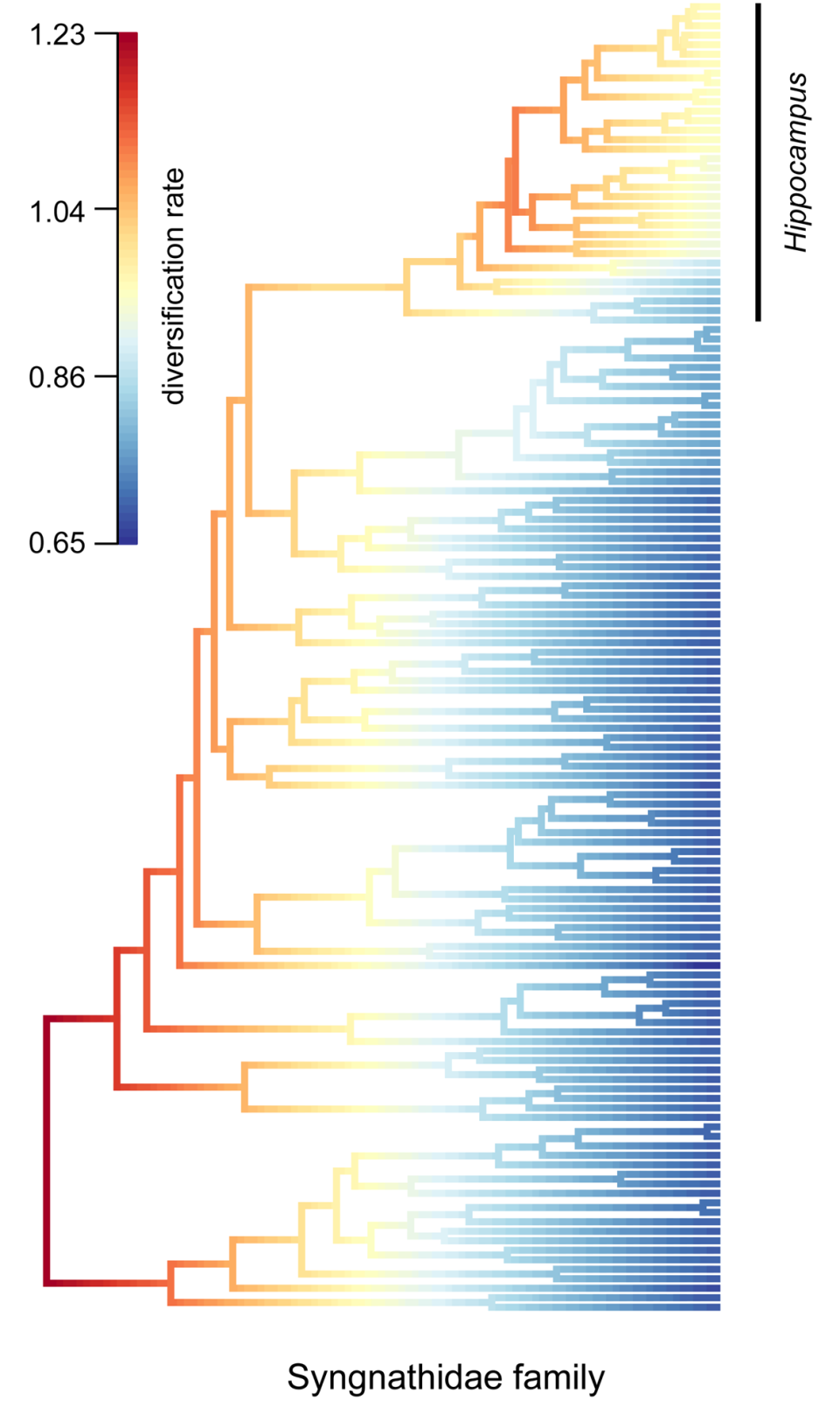


Supplementary Fig. 1 Color-coded phylogenetic tree indicating the diversification rate estimated for the *Syngnathidae* family using BAMM. Seahorses (genus *Hippocampus*) display the greatest diversification rates compared to the rest of the family (yellow-red branches). Source data are provided at Figshare (Dataset 1).


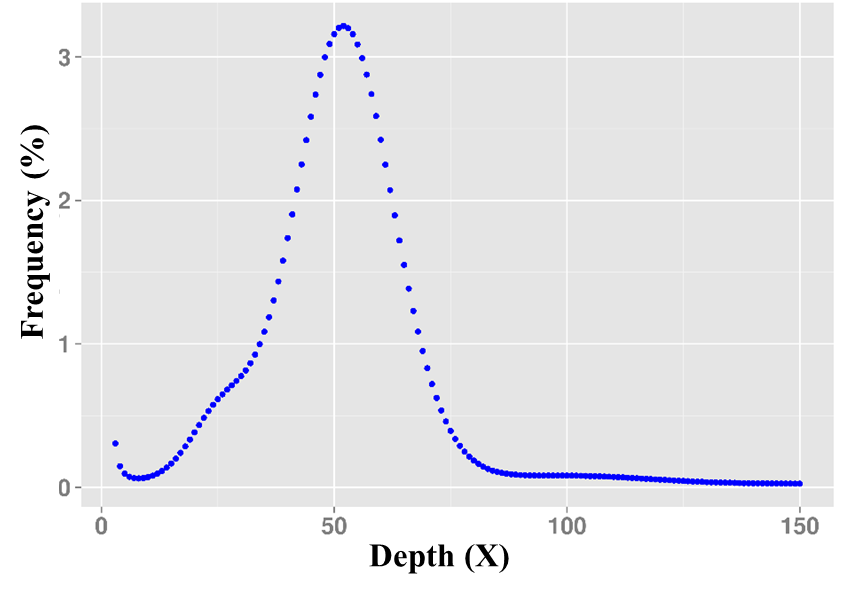


Supplementary Fig. 2 Estimation of the *Hippocampus erectus* genome size based on *19-mer* statistics. The *x* axis represents the depth while the *y* axis represents the proportion of 19-mer with different coverage.


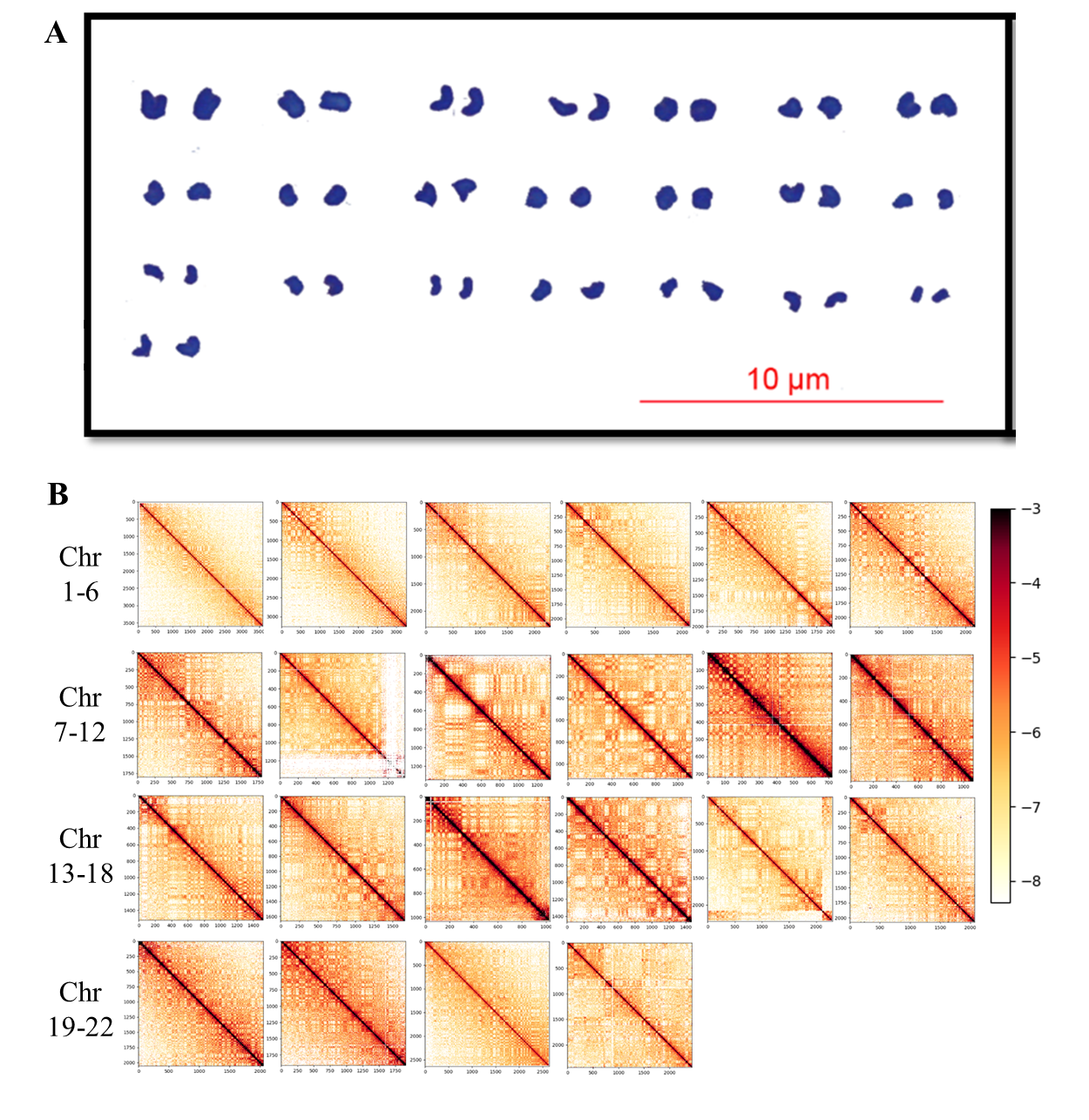


Supplementary Fig. 3 Karyotype analysis of *Hippocampus erectus*. 22 pairs of chromosomes at the mitotic stage were identified with Giemsa staining. The results are a summary of the data obtained from 20 lined seahorses.


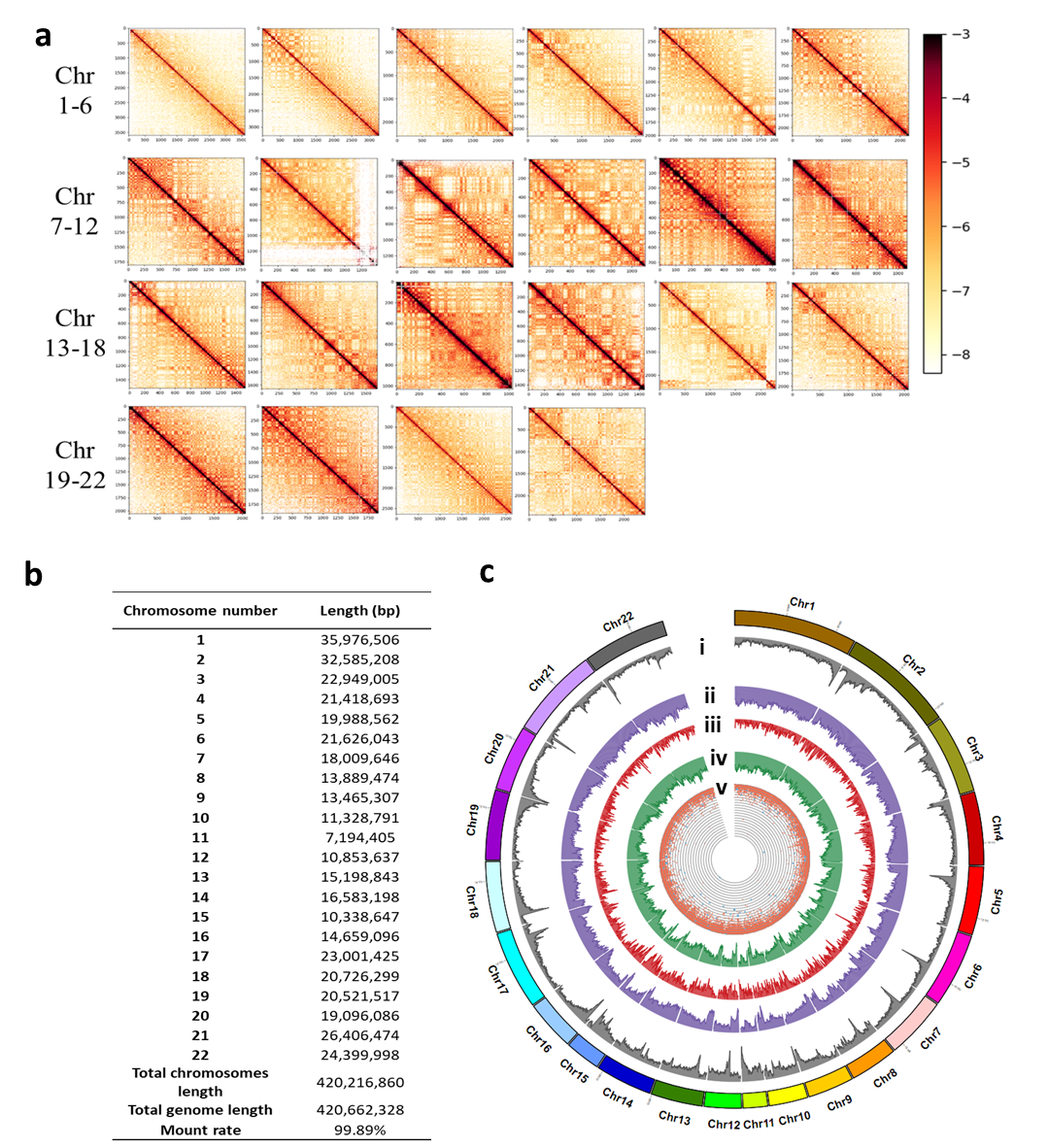


Supplementary Fig. 4 Characterization of the *Hippocampus erectus* genome.

a, Hi-C contact map of the 22 assembled chromosomes: the heat map of the assembled genome shows more frequent interactions with all loci within the same ‘mega domains’. b, Statistics of chromosome lengths after Hi-C scaffolding. c, Circos plot of the multidimensional topography of the *H. erectus* genome, including (i) GC content, (ii) Repeat element density, (iii) Gene density, (iv) SNP density, and (v) Positive selection result of spine trait, in which the genes with lower *p* value are plotted closer to the circle center. Source data are provided as a Source Data file.


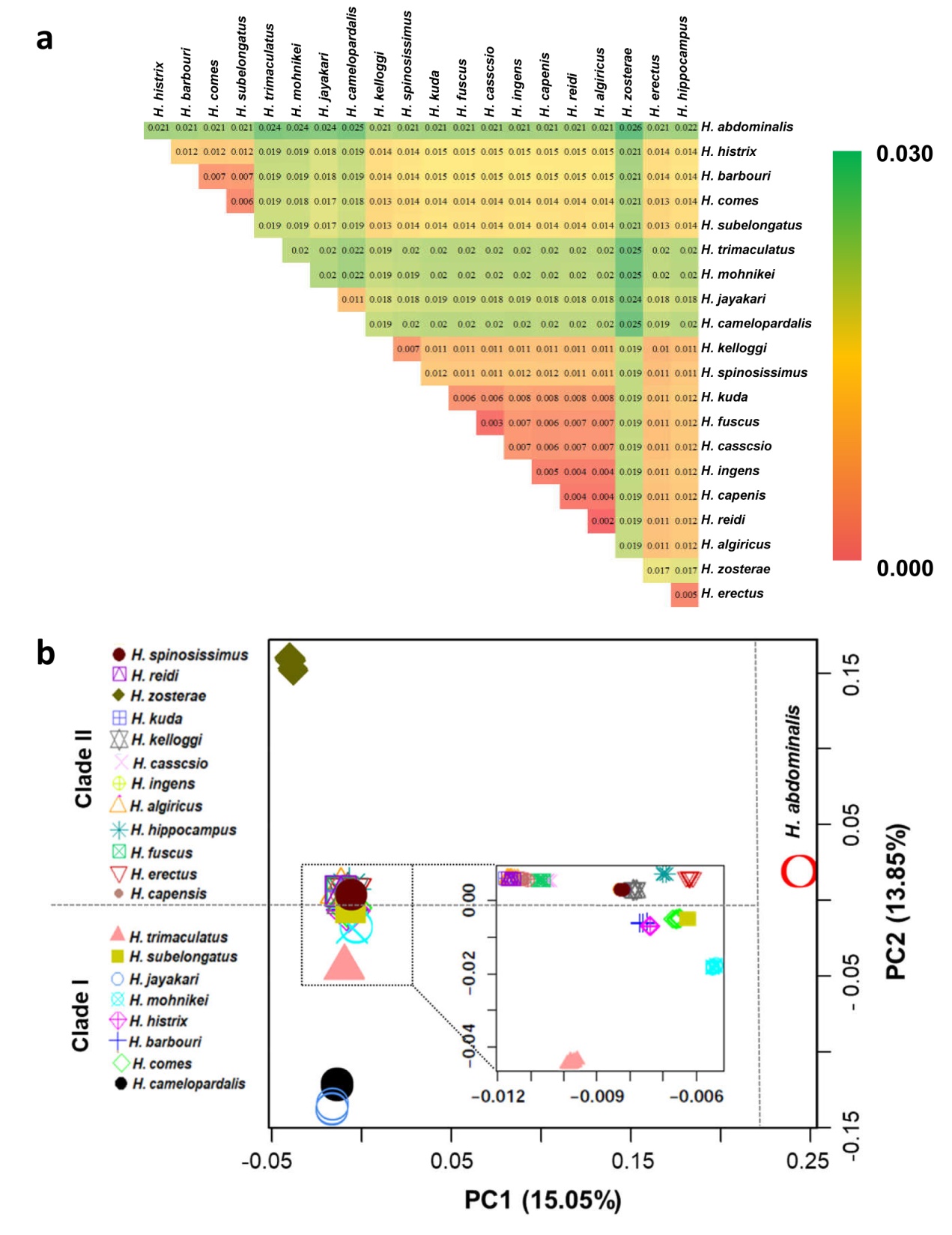


Supplementary Fig. 5 Genetic divergence and structure of *Hippocampus*. a, The genomic divergence between each pair of the 21 sampled seahorse species. Divergence between seahorse species ranged from 0.2% (*H. algiricus* and *H. reidi*) to 2.6% (*H. zosterae* and *H. abdominalis*). In addition, six species including *H. abdominalis*, *H. zosterae*, *H. jayakari,* *H. camelopardalis*, *H. trimaculatus*, and *H. mohnikei* were genetically distinct from the other species. b, Principal component analysis (PCA) using all the SNPs of the 358 specimens. The vertical grey dashed line shows the separation of *H. abdominalis* with the other seahorse species by PC1 (15.5%), while the horizontal grey dashed line indicates the division of Clade I and Clade II by PC2 (13.85%). Source data are provided as a Source Data file.


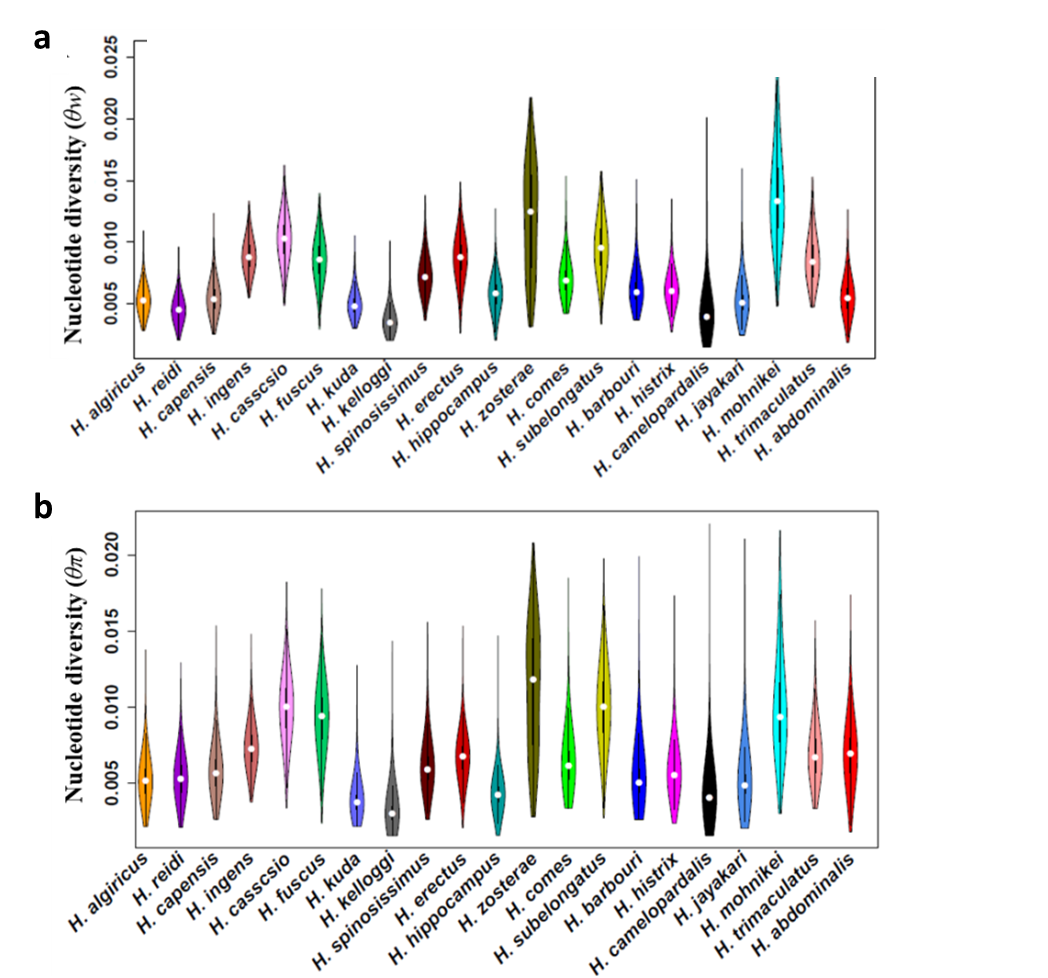


Supplementary Fig. 6 Violin plots depicting the nucleotide diversity. a, Watterson (*θw*) estimators of nucleotide diversity. b, Pairwise (*θπ*) estimators of nucleotide diversity. White dot, median; bar limits, upper and lower quartiles; whiskers, 1.5 × interquartile range. Sample size of each species is summarized in Supplementary Table 6. Sliding-window method was employed and source data are available at Figshare (Dataset 2).


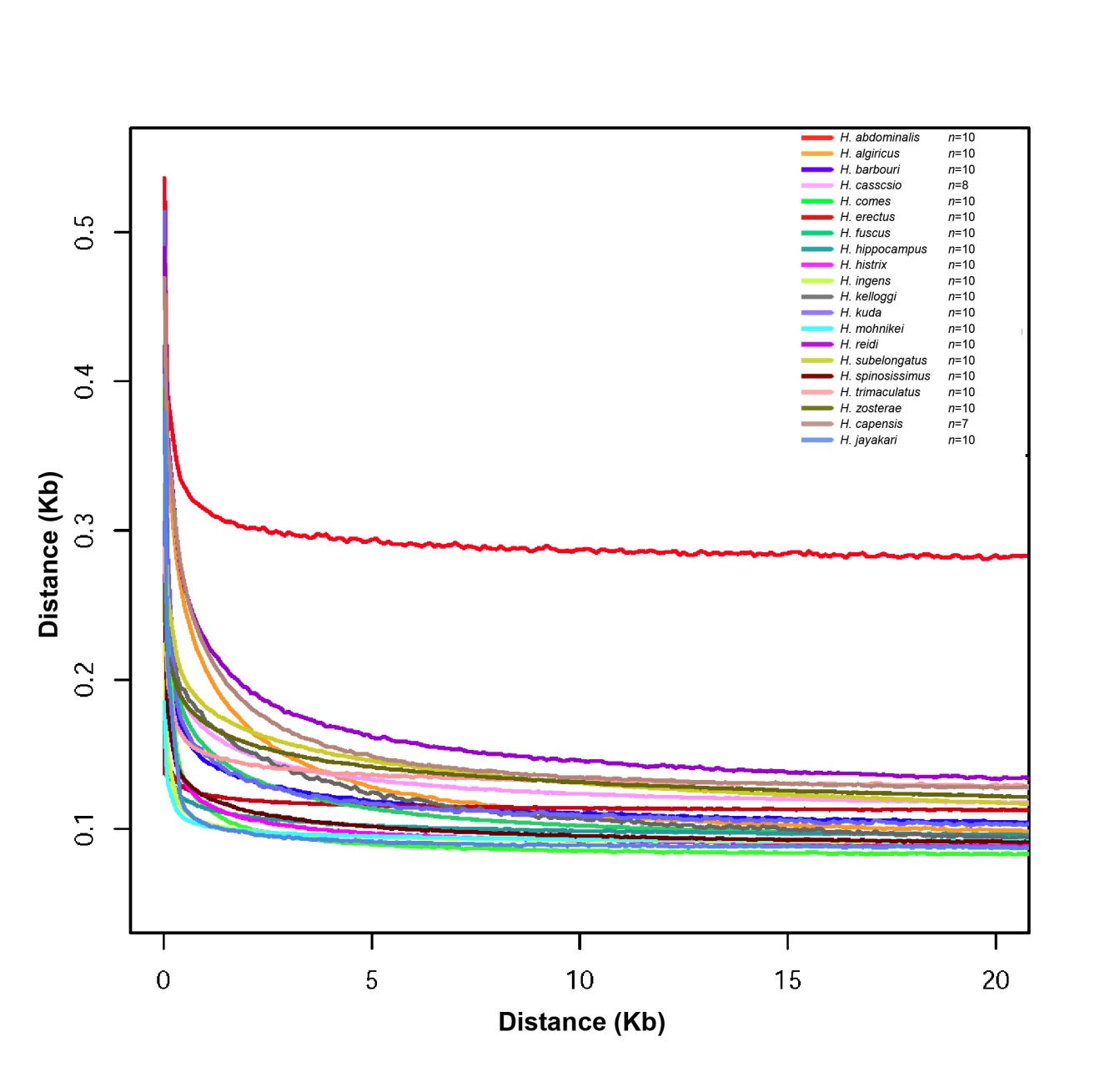


Supplementary Fig. 7 Linkage disequilibrium decay analysis. Bracketed digits in the legend indicate randomly selected specimens used for each of the species. Source data are provided as a Source Data file.


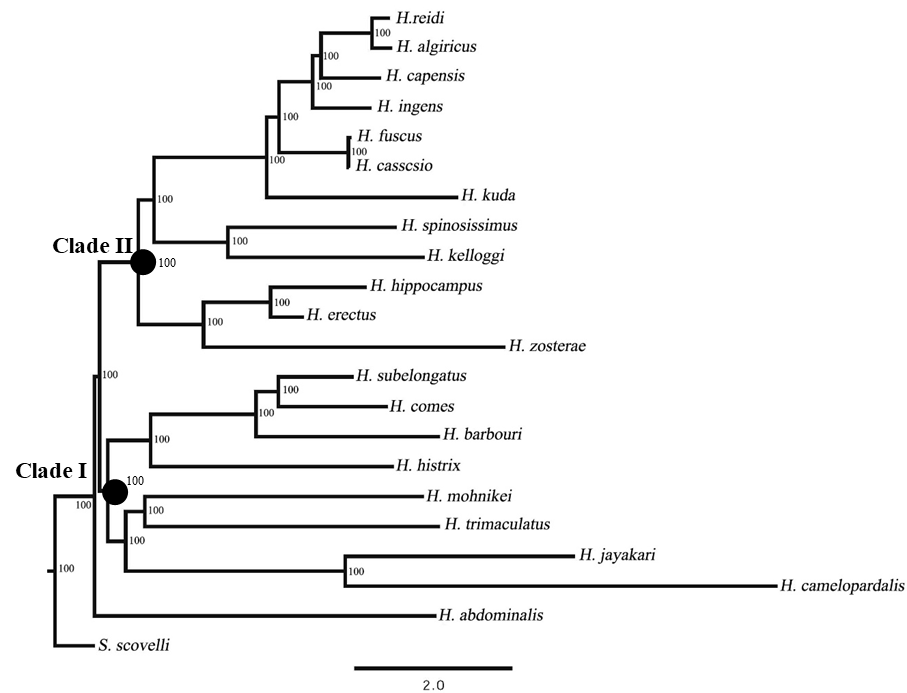


Supplementary Fig. 8 Coalescent-based phylogenetic tree of the genus *Hippocampus.* The species tree was inferred using ASTRAL with *Syngnathus scovelli* as the outgroup. Black circles indicate two distinct major clades in addition to the lineage of *H. abdominalis*. A total of 2,000 independent genes and 103 specimens (1-5 samples for each species) were used. Gene trees were generated using RAxML (v8) using the rapid bootstrap analysis and searched for the best-scoring maximum likelihood tree (option a) under a GTR+G substitution model. The number at each node indicates the bootstrap percentage after 100 replications. Source data are available at Figshare (Datasets 4-5).


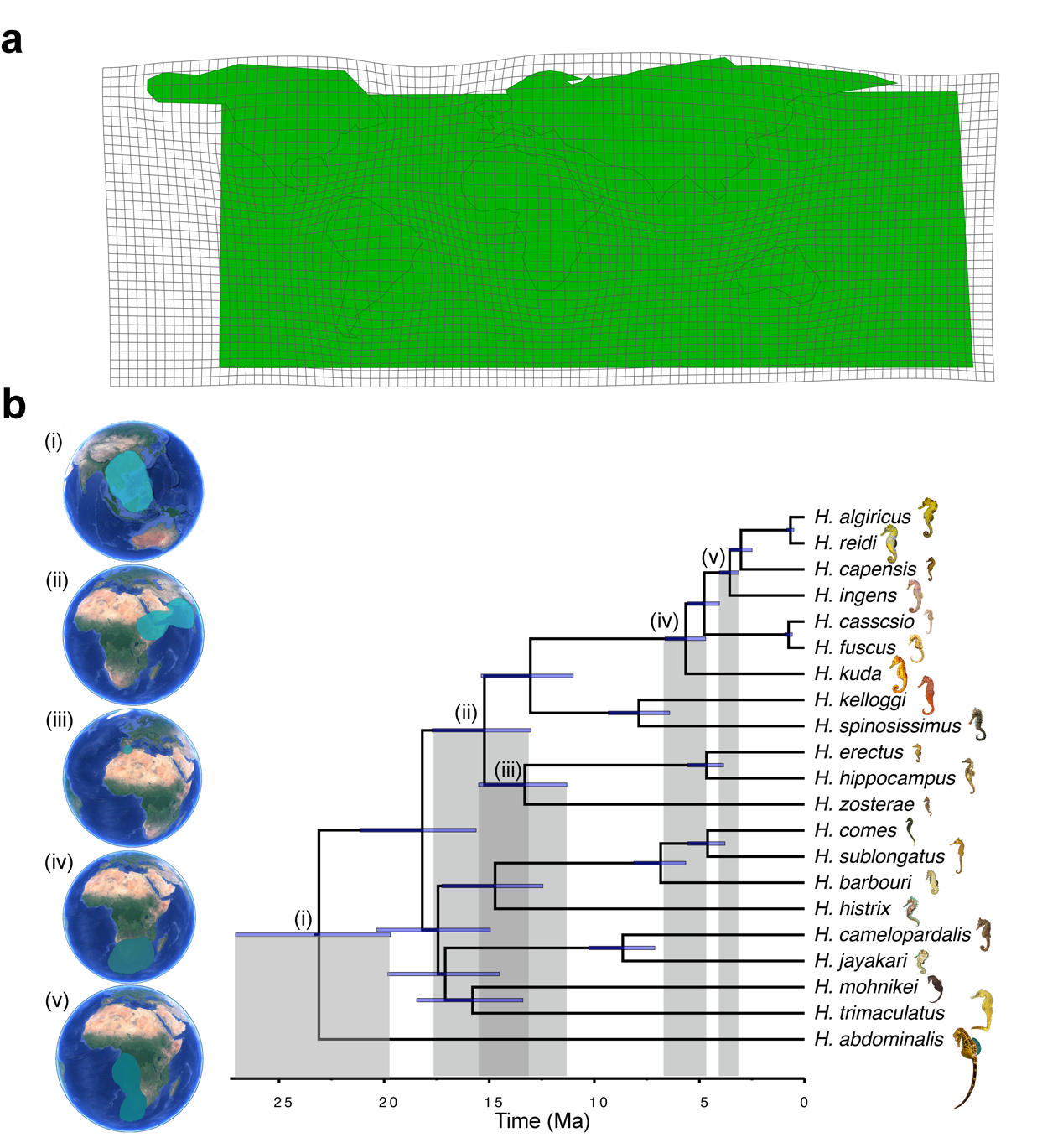


Supplementary Fig. 9 Diversification analysis in space and time. a, Deformation grid used for heterogeneous landscape modeling, using the parameters: deformation = 20, value = 2. b, The right panel shows the phylogenetic tree. The left panel depicts the maps showing the geographic location of BEAST ancestral reconstructions, under a heterogeneous model (deformation = 20, value = 2). The labels (i) – (v) are in correspondence to the geographic location of ancestral reconstructed on the species tree (nodes). The map (i) corresponds to the reconstructed origin of the ancestor for all *Hippocampus* species included here (root). This result indicates that the seahorse colonization occurred twice through the Atlantic, (ii) to (iii) correspond to expansion through the opening of the Tethys seaway during the Middle Miocene, and (iv) to (v) correspond to second invasion to the Atlantic. Source data are available at Figshare (Datasets 4-6). Maps modified from Google Earth v7.1. Seahorses illustrations by Geng Qin.


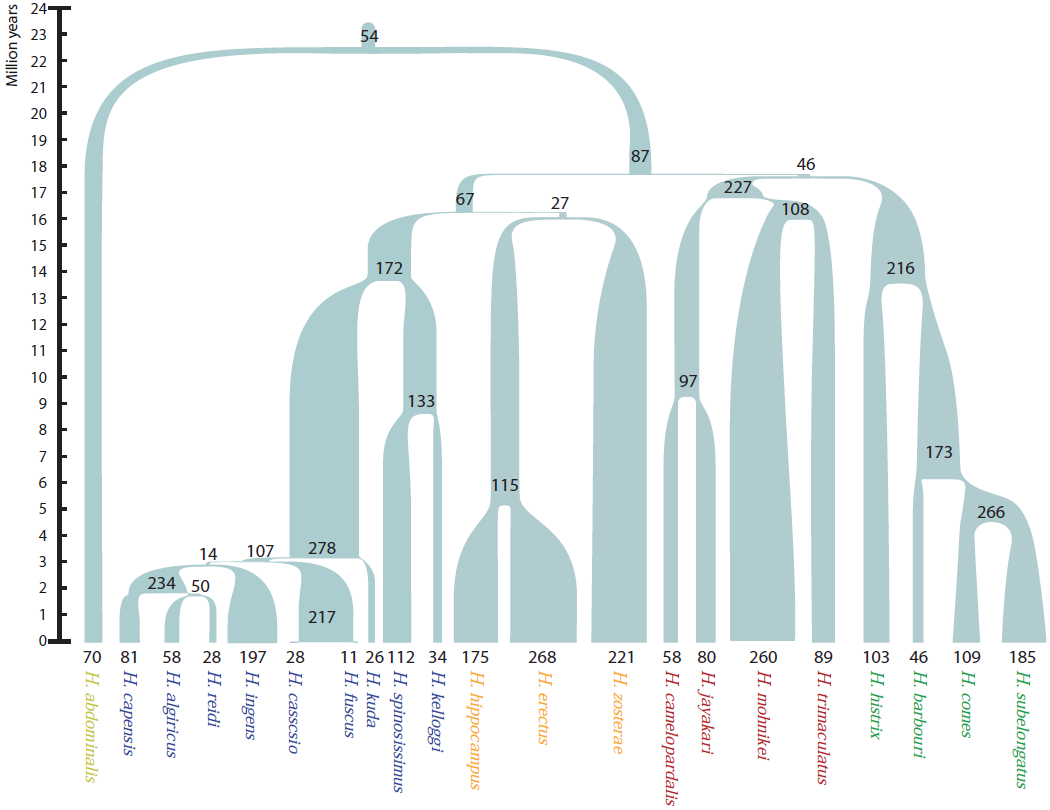


Supplementary Fig. 10 Inference of divergence time and ancestral population size by G-PhoCS. Digits on the tree indicate ancestral population size, while the y-axis indicates the divergence time.


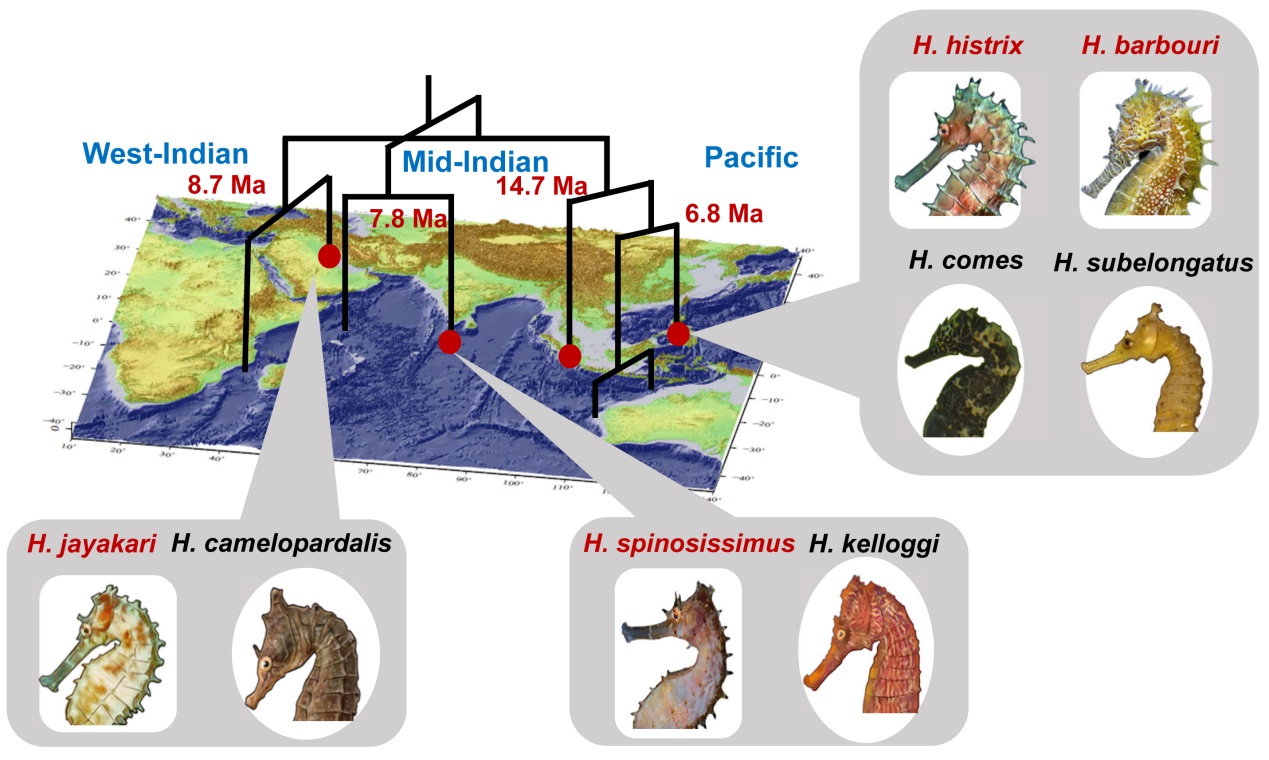


Supplementary Fig. 11 Convergent evolution of the spine trait across the Indian and Pacific radiations. The simplified phylogenetic tree includes all four spiny seahorse species as well as their sister lineages. Spiny seahorses are marked with red colors. Digits labeled on the node of branches indicate divergence time between each spiny and non-spiny lineage. Maps from Wessel et al. (2019) under GNU GPL license^41^. Seahorses illustrations by Geng Qin.


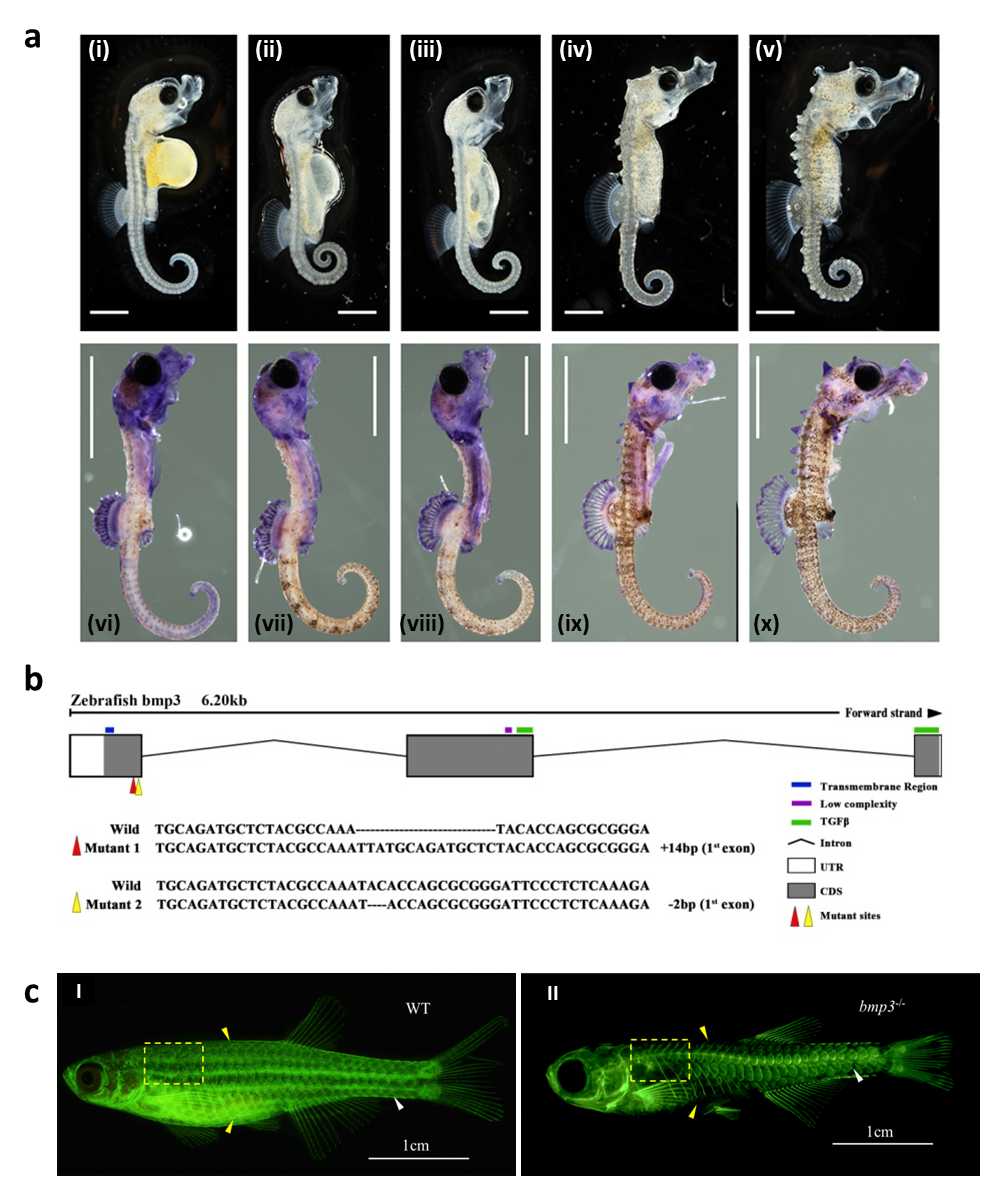


Supplementary Fig. 12 Function of the *bmp3* gene. a, *In situ* hybridization for *bmp3* in *Hippocampus erectus* throughout developmental stages in which bony spines emerge (~ 5 to 1 day prior to birth). i-v: light microscopy pictures. vi-x: embryos after*in situ* hybridization using a *bmp3* probe. At least three replicates of relevant embryoic developmental stages of the seahorse were used. Purple stain mostly reflects *bmp3* gene expression, however, stain in the buccal cavity and gut area is likely false signal from endogenous alkaline phosphatase activity. Scale bars are 1 mm. *In situ* photos of seahorses by Ralf F. Schneider. b, Sequence characteristics of *bmp3* in wild and mutant zebrafish. c, A series of significant scale defects were found in homozygous *bmp3*^-/-^ zebrafish, such as decrements in scale numbers, rearrangements, and irregular shapes. The F2 *bmp3*^+14^ mutant fishes gave 4/29 fish with scale defects, whereas 3/31 had scale defects for F2 *bmp3*^-2^ mutant fish. Gene knockout photos of seahorses by Shiming Wan. Source Data are provided as a Source Data file.

Supplementary Tables

Supplementary Table 1 Genome size estimation of the *H. erectus* based on the *K*-mer spectrum.

| *K-mer* Size | Genome Size (bp) | Heterozygosity (%) | Repeat (%) |
| --- | --- | --- | --- |
| 19 | 414566654 | 0.56 | 18.63 |
| 21 | 411527266 | 0.61 | 15.79 |
| 23 | 412081781 | 0.58 | 14.75 |
| 25 | 412774005 | 0.57 | 13.76 |
| 27 | 409744137 | 0.59 | 13.03 |
| 29 | 410299618 | 0.56 | 12.53 |

Supplementary Table 2 Summary of the PacBio genome assembly of *H. erectus*.

| Genome assembly parameter | Details |
| --- | --- |
| Number of contigs | 187 |
| Contigs total length (bp) | 420,662,328 |
| Contig N50 (bp) | 15,499,254 |
| Contig N90 (bp) | 3,013,571 |
| Contig max (bp) | 24,351,324 |
| GC content (%) | 43.66 |

Supplementary Table 3 Summary of predicted gene models in the *H. erectus* genome.

| Method | Software | Species | Gene number |
| --- | --- | --- | --- |
| *Ab initio* | Genscan | - | 23,546 |
|  | Augustus | - | 25,374 |
|  | GlimmerHMM | - | 73,265 |
|  | GeneID | - | 39,.39 |
|  | SNAP | - | 35,256 |
| Homology-based | GeMoMa | *Danio rerio* | 18,840 |
|  |  | *Hippocampus comes* | 19,860 |
|  |  | *Oryzias latipes* | 19,186 |
|  |  | *Xiphophorus maculatus* | 18,649 |
| RNAseq | PASA | *-* | 158,577 |
|  | TransDecoder | *-* | - |
|  | GeneMarkS-T | - | - |
| Integration | EVM | - | 20,137 |

Supplementary Table 4 The number of genes annotated by different databases.

| Annotation database | Number of genes | Percentage (%) |
| --- | --- | --- |
| GO | 7,530 | 37.39 |
| KEGG | 12,200 | 60.58 |
| KOG | 14,418 | 71.60 |
| TrEMBL | 18,208 | 90.42 |
| NR | 18,241 | 90.58 |
| All | 19,335 | 96.02 |

Supplementary Table 5 Summary of mapping statistics towards different reference genomes.

| Species | Mapping rate  (%) | | Average depth (X) | | Genome coverage (%) | |
| --- | --- | --- | --- | --- | --- | --- |
|  | *H. erectus* | *H. comes* | *H. erectus* | *H. comes* | *H. erectus* | *H. comes* |
| *H. abdominalis* | 91.28 | 85.39 | 17.17 | 16.11 | 86.04 | 82.01 |
| *H. algiricus* | 92.53 | 86.20 | 14.35 | 13.54 | 92.87 | 87.61 |
| *H. barbouri* | 86.71 | 95.72 | 12.52 | 12.13 | 90.64 | 93.52 |
| *H. casscsio* | 94.68 | 89.22 | 12.78 | 11.28 | 93.31 | 87.73 |
| *H. comes* | 71.92 | 93.87 | 10.03 | 9.97 | 90.23 | 94.83 |
| *H. erectus* | 96.73 | 88.87 | 15.01 | 13.16 | 99.06 | 85.51 |
| *H. fuscus* | 95.29 | 91.29 | 16.80 | 15.37 | 93.53 | 88.64 |
| *H. hippocampus* | 94.58 | 84.96 | 14.39 | 12.38 | 97.79 | 84.29 |
| *H. histrix* | 93.29 | 92.74 | 13.60 | 12.61 | 91.14 | 91.17 |
| *H. ingens* | 93.04 | 87.30 | 13.94 | 12.04 | 92.83 | 87.41 |
| *H. kelloggi* | 92.94 | 90.16 | 12.70 | 11.42 | 93.64 | 89.11 |
| *H. kuda* | 90.59 | 85.17 | 14.32 | 12.63 | 92.73 | 87.40 |
| *H. mohnikei* | 85.25 | 82.16 | 14.28 | 12.89 | 86.40 | 82.58 |
| *H. reidi* | 92.81 | 87.30 | 14.56 | 13.08 | 92.89 | 87.63 |
| *H. subelongatus* | 87.42 | 97.00 | 14.64 | 14.17 | 91.92 | 95.16 |
| *H. spinosissimus* | 94.76 | 90.96 | 13.63 | 12.19 | 93.71 | 89.20 |
| *H. trimaculatus* | 93.05 | 87.69 | 16.04 | 14.31 | 86.55 | 83.47 |
| *H. zosterae* | 90.14 | 80.22 | 16.84 | 14.13 | 90.43 | 79.15 |
| Average | 90.94 | 88.68 | 14.31 | 12.97 | 91.98 | 87.58 |

Supplementary Table 6 Summary of seahorse re-sequencing data.

| Species | Number | Clean data (bp) | Clean reads | Mapped reads | Mapping rate | Depth (X) | 1X Coverage | 4X Coverage |
| --- | --- | --- | --- | --- | --- | --- | --- | --- |
| *H. abdominalis* | 16 | 8,590,802,794 | 57,272,019 | 52,278,505 | 91.28% | 17.17 | 86.04% | 81.81% |
| *H. algiricus* | 18 | 7,141,870,133 | 47,612,468 | 44,056,085 | 92.53% | 14.35 | 92.87% | 88.75% |
| *H. barbouri* | 20 | 6,964,138,080 | 46,427,587 | 40,262,209 | 86.71% | 12.52 | 90.64% | 83.96% |
| *H. casscsio* | 8 | 6,248,824,650 | 41,658,831 | 39,464,550 | 94.68% | 12.78 | 93.31% | 87.31% |
| *H. camelopardalis* | 2 | 5,462,617,725 | 36,417,452 | 29,205,731 | 80.48% | 9.39 | 86.80% | 76.61% |
| *H. comes* | 19 | 6,737,105,463 | 44,914,036 | 32,380,259 | 71.92% | 10.03 | 90.23% | 76.71% |
| *H. capensis* | 7 | 6,556,851,536 | 43,712,344 | 38,649,001 | 88.35% | 12.81 | 92.82% | 88.61% |
| *H. erectus* | 21 | 6,984,926,414 | 46,566,176 | 45,077,490 | 96.73% | 15.01 | 99.06% | 97.67% |
| *H. fuscus* | 19 | 8,046,183,782 | 53,641,225 | 51,078,664 | 95.29% | 16.80 | 93.53% | 89.52% |
| *H. hippocampus* | 16 | 6,759,862,725 | 45,065,752 | 42,598,229 | 94.58% | 14.39 | 97.79% | 94.97% |
| *H. histrix* | 22 | 6,989,881,316 | 46,599,209 | 43,374,686 | 93.29% | 13.60 | 91.14% | 85.56% |
| *H. ingens* | 20 | 6,869,668,230 | 45,797,788 | 42,624,704 | 93.04% | 13.94 | 92.83% | 88.13% |
| *H. jayakari* | 20 | 6,499,906,688 | 43,332,711 | 39,675,872 | 91.56% | 13.29 | 87.87% | 83.11% |
| *H. kelloggi* | 20 | 6,317,769,098 | 42,118,461 | 39,176,438 | 92.94% | 12.70 | 93.64% | 88.04% |
| *H. kuda* | 18 | 7,294,594,042 | 48,630,627 | 43,977,830 | 90.59% | 14.32 | 92.73% | 88.17% |
| *H. mohnikei* | 19 | 7,840,299,868 | 52,268,666 | 44,528,851 | 85.25% | 14.28 | 86.40% | 78.95% |
| *H. reidi* | 19 | 7,148,416,555 | 47,656,110 | 44,126,794 | 92.81% | 14.56 | 92.89% | 89.12% |
| *H. subelongatus* | 13 | 7,983,445,835 | 53,222,972 | 46,503,423 | 87.42% | 14.64 | 91.92% | 87.22% |
| *H. spinosissimus* | 20 | 6,682,367,325 | 44,549,116 | 42,192,349 | 94.76% | 13.63 | 93.71% | 88.04% |
| *H. trimaculatus* | 20 | 7,582,358,265 | 50,549,055 | 47,045,025 | 93.05% | 16.04 | 86.55% | 81.66% |
| *H. zosterae* | 21 | 8,243,407,307 | 54,956,049 | 49,485,458 | 90.14% | 16.84 | 90.43% | 87.15% |
| Total/Mean | 358 | 7,092,633,230 | 47,284,222 | 42,750,579 | 90.35% | 13.96 | 91.58% | 86.24% |

Supplementary Table 7 Summary of variant statistics for each of the 21 species compared with *H. erectus* genome.

| Species | Number | Total | Intergenic | Upstream/ Downstream | Intronic | Splicing | UTR3 | UTR5 | UTR5/  UTR3 | Exonic |
| --- | --- | --- | --- | --- | --- | --- | --- | --- | --- | --- |
|  |  |  |  |  |  |  |  |  |  |  |
| *H. abdominalis* | 16 | 3,387,029 | 1,132,590 | 200,408 | 1,469,688 | 481 | 133,910 | 38,835 | 136 | 410,981 |
| *H. algiricus* | 18 | 2,486,541 | 856,513 | 146,787 | 1,076,925 | 464 | 92,444 | 27,289 | 83 | 286,036 |
| *H. barbouri* | 20 | 2,964,563 | 1,012,090 | 172,203 | 1,277,496 | 515 | 112,926 | 32,936 | 110 | 356,287 |
| *H. casscsio* | 8 | 2,995,764 | 1,028,632 | 177,341 | 1,315,933 | 534 | 109,191 | 31,613 | 100 | 332,420 |
| *H. camelopardalis* | 2 | 3,123,978 | 1,050,925 | 180,966 | 1,356,410 | 511 | 124,087 | 36,802 | 128 | 374,149 |
| *H. comes* | 19 | 2,898,209 | 986,073 | 171,923 | 1,250,248 | 555 | 111,054 | 32,739 | 114 | 345,503 |
| *H. capensis* | 7 | 2,438,483 | 837,953 | 144,461 | 1,064,766 | 388 | 90,810 | 25,910 | 82 | 274,113 |
| *H. erectus* | 21 | 3,889,601 | 1,516,107 | 223,790 | 1,697,212 | 601 | 122,911 | 31,607 | 128 | 297,245 |
| *H. fuscus* | 19 | 3,465,954 | 1,194,517 | 205,967 | 1,521,839 | 590 | 126,733 | 36,842 | 121 | 379,345 |
| *H. hippocampus* | 16 | 2,683,085 | 996,745 | 160,665 | 1,167,380 | 500 | 93,642 | 26,049 | 91 | 238,013 |
| *H. histrix* | 22 | 3,260,516 | 1,128,001 | 194,590 | 1,417,005 | 586 | 127,464 | 37,003 | 138 | 355,729 |
| *H. ingens* | 20 | 3,870,374 | 1,338,085 | 227,150 | 1,679,823 | 680 | 146,397 | 41,809 | 137 | 436,293 |
| *H. jayakari* | 20 | 3,621,728 | 1,237,945 | 212,625 | 1,561,615 | 735 | 144,271 | 43,023 | 166 | 421,348 |
| *H. kelloggi* | 20 | 2,044,762 | 709,222 | 121,511 | 885,541 | 391 | 75,058 | 21,937 | 72 | 231,030 |
| *H. kuda* | 18 | 2,231,499 | 770,448 | 132,343 | 964,417 | 396 | 83,612 | 24,382 | 79 | 255,822 |
| *H. mohnikei* | 19 | 4,983,571 | 1,685,986 | 287,952 | 2,142,459 | 850 | 199,644 | 58,138 | 180 | 608,362 |
| *H. reidi* | 19 | 2,297,340 | 787,559 | 136,279 | 1,003,745 | 359 | 85,628 | 24,710 | 70 | 258,990 |
| *H. subelongatus* | 13 | 3,398,108 | 1,144,880 | 202,746 | 1,500,578 | 470 | 126,637 | 36,373 | 143 | 386,281 |
| *H. spinosissimus* | 20 | 3,350,854 | 1,167,806 | 198,722 | 1,464,166 | 613 | 123,521 | 35,124 | 109 | 360,793 |
| *H. trimaculatus* | 20 | 3,657,718 | 1,217,460 | 217,057 | 1,569,517 | 760 | 148,108 | 45,600 | 167 | 459,049 |
| *H. zosterae* | 21 | 5,452,148 | 1,837,291 | 335,300 | 2,413,846 | 1,077 | 217,266 | 61,468 | 237 | 585,663 |
| Total | 358 | 41,794,569 | 14,741,299 | 2,508,406 | 18,329,362 | 8,556 | 1,601,093 | 467,951 | 1,657 | 4,136,245 |

Intergenic: variant is in intergenic region

Upstream/Downstream: variant overlaps 1-kb region upstream or downstream of transcription start site

UTR5/UTR3: variant overlaps an UTR5/UTR3

Intronic: variant overlaps an intron

Splicing: variant is within 2-bp of a splicing junction

Exonic: variant overlaps a coding region

Supplementary Table 8 Summary of SNP statistics within each of the 21 seahorse species.

| Species | Number | Total | Intergenic | Upstream/ Downstream | Intronic | Splicing | UTR3 | UTR5 | UTR5; UTR3 | Exonic |
| --- | --- | --- | --- | --- | --- | --- | --- | --- | --- | --- |
|  |  |  |  |  |  |  |  |  |  |  |
| *H. abdominalis* | 16 | 948,040 | 324,475 | 59,403 | 432,639 | 176 | 133,910 | 38,835 | 136 | 88,435 |
| *H. algiricus* | 18 | 1,268,291 | 450,291 | 75,476 | 546,654 | 328 | 92,444 | 27,289 | 83 | 137,210 |
| *H. barbouri* | 20 | 1,486,820 | 521,929 | 87,676 | 639,307 | 350 | 112,926 | 32,936 | 110 | 167,898 |
| *H. casscsio* | 10 | 454,895 | 163,685 | 27,030 | 198,224 | 126 | 109,191 | 31,613 | 100 | 44,928 |
| *H. camelopardalis* | 2 | 1,231,040 | 434,780 | 73,235 | 540,740 | 240 | 124,087 | 36,802 | 128 | 126,185 |
| *H. comes* | 19 | 2,099,296 | 728,052 | 124,335 | 928,994 | 428 | 111,054 | 32,739 | 114 | 222,469 |
| *H. capensis* | 7 | 1,957,541 | 677,545 | 118,764 | 844,121 | 458 | 90,810 | 25,910 | 82 | 220,482 |
| *H. erectus* | 21 | 3,885,747 | 1,514,537 | 223,584 | 1,695,680 | 600 | 122,911 | 31,607 | 128 | 296,790 |
| *H. fuscus* | 17 | 2,576,542 | 898,499 | 154,011 | 1,141,918 | 480 | 126,733 | 36,842 | 121 | 263,375 |
| *H. hippocampus* | 16 | 2,310,696 | 859,252 | 138,563 | 1,006,137 | 457 | 93,642 | 26,049 | 91 | 202,964 |
| *H. histrix* | 22 | 1,672,684 | 604,798 | 102,797 | 726,787 | 402 | 127,464 | 37,003 | 138 | 155,619 |
| *H. ingens* | 20 | 2,767,588 | 967,577 | 163,069 | 1,203,225 | 562 | 146,397 | 41,809 | 137 | 301,205 |
| *H. jayakari* | 20 | 1,671,721 | 598,382 | 98,555 | 725,350 | 435 | 144,271 | 43,023 | 166 | 167,269 |
| *H. kelloggi* | 20 | 749,192 | 274,680 | 45,898 | 319,361 | 248 | 75,058 | 21,937 | 72 | 76,220 |
| *H. kuda* | 18 | 812,948 | 291,344 | 48,614 | 343,041 | 219 | 83,612 | 24,382 | 79 | 93,035 |
| *H. mohnikei* | 19 | 3,626,099 | 1,254,702 | 210,675 | 1,577,995 | 682 | 199,644 | 58,138 | 180 | 399,990 |
| *H. reidi* | 19 | 1,220,145 | 433,294 | 72,770 | 537,080 | 226 | 85,628 | 24,710 | 70 | 121,056 |
| *H. subelongatus* | 13 | 2,177,843 | 780,353 | 130,672 | 953,906 | 483 | 126,637 | 36,373 | 143 | 212,389 |
| *H. spinosissimus* | 20 | 2,319,942 | 795,055 | 140,322 | 1,041,149 | 349 | 123,521 | 35,124 | 109 | 235,635 |
| *H. trimaculatus* | 20 | 1,618,966 | 547,935 | 98,657 | 689,659 | 461 | 148,108 | 45,600 | 167 | 196,555 |
| *H. zosterae* | 21 | 3,914,728 | 1,293,689 | 248,347 | 1,756,159 | 867 | 217,266 | 61,468 | 237 | 412,592 |
| All | 358 | 31,462,813 | 11,178,032 | 1,889,941 | 13,760,174 | 7,161 | 1,601,093 | 467,951 | 1,657 | 3,105,329 |

Detailed descriptions for the annotation of the variants are shown in Supplementary Table 7.

Supplementary Table 9 Details of the parameter values used to model the calibration through hyperpriors on node ages in the BEAST analysis of diversification in space and time.

| Node | Distribution | Mean | SD | Offset | Median | 95% HPD |
| --- | --- | --- | --- | --- | --- | --- |
| *Hippocampus* root | Lognormal | 9 | 0.6 | 11.6 | 20.5 | 14.4-31.8 |
| *H. sarmaticus* fossil (sister to *H. trimaculatus*) | Lognormal | 11.8 | 0.2 | 0 | 11.6 | 8.32-16.1 |
| Closure of West Atlantic – East Pacific (split between *H. reidi* and *H. ingens*) | Lognormal | 1.2 | 0.4 | 2.8 | 3.61 | 3.07-4.64 |

Supplementary Table 10 Inference of gene flow by G-PhoCS.

| Migration bands | Total migration rates (*m*) | | | Probability of gene flow (*p*) | | |
| --- | --- | --- | --- | --- | --- | --- |
|  | Posterior mean | 95% Bayesian credible intervals | | Posterior mean | 95% Bayesian credible intervals | |
|  |  | Lower | Upper |  | Lower | Upper |
| Hag -> Hrd | 0.196299485 | 0.143939172 | 0.249898341 | 0.178233909 | 0.13405958 | 0.221120041 |
| Hrd -> Hag | 0.052629619 | 0.023994315 | 0.079960939 | 0.051268661 | 0.02370874 | 0.076847595 |
| Hcm -> Hsl | 0.015261824 | 0.011447067 | 0.018920953 | 0.015145953 | 0.011381799 | 0.018743075 |
| Hag -> Hcp | 0.007346289 | 0.004168275 | 0.010578734 | 0.007319371 | 0.0041596 | 0.010522976 |
| Hig -> Hcp | 0.006888639 | 0.004699862 | 0.009254287 | 0.006864967 | 0.004688835 | 0.009211598 |
| Hcp -> Hrd | 0.006511778 | 0.001879971 | 0.011240501 | 0.006490623 | 0.001878205 | 0.011177562 |
| Hcm -> Hbb | 0.006494862 | 0.003704366 | 0.009525422 | 0.006473816 | 0.003697514 | 0.009480199 |
| Hhc -> Het | 0.003499631 | 0.000148435 | 0.005764995 | 0.003493515 | 0.000148424 | 0.005748409 |
| Hig -> Hrd | 0.0034827 | 0.000575301 | 0.006311777 | 0.003476643 | 0.000575136 | 0.0062919 |
| Hag -> Hig | 0.00227131 | 0.001276748 | 0.003311016 | 0.002268733 | 0.001275934 | 0.00330554 |
| Het -> Hag | 0.002120481 | 0.000901629 | 0.003343067 | 0.002118235 | 0.000901223 | 0.003337485 |
| Hbb -> Hsl | 0.00179019 | 0.000545829 | 0.003150343 | 0.001788588 | 0.00054568 | 0.003145386 |
| Hcp -> Hfc | 0.001478112 | 0.000300557 | 0.003316637 | 0.001477021 | 0.000300511 | 0.003311143 |
| Hcp -> Hcc | 0.001342088 | 0.000224469 | 0.003240196 | 0.001341188 | 0.000224443 | 0.003234952 |
| Hsl -> Hbb | 0.00132424 | 2.42986E-05 | 0.002952178 | 0.001323364 | 2.42983E-05 | 0.002947824 |
| Hcc -> Hcp | 0.001252542 | 0.00021889 | 0.002778713 | 0.001251758 | 0.000218866 | 0.002774856 |

Symbol -> showed the direction of gene flow. Abbreviations stand for: *H. algiricus* (Hag), *H. reidi* (Hrd) , *H. comes* (Hcm) , *H. subelongatus* (Hsl), *H. capensis* (Hcp) , *H. ingens* (Hig), *H. barbouri* (Hbb) , *H. hippocampus* (Hhc), *H. erectus* (Het), *H. fuscus* (Hfc), *H. casscsio* (Hcc).

Supplementary Table 11 Primers used for *in situ* hybridization and gene knockout of bmp3 knockout.

| Primer Name | Sequence | Description |
| --- | --- | --- |
| bottom strand Ultramer | GATCCGCACCGACTCGGTGCCACTTTTTCAAGTTGATAACGGACTAGCCTTATTTTAACTTGCTATTTCTAGCTCTAAAAC | CRISPR-Cas9,  generic DNA oligo |
| dre-bmp3-gRNA1 oligo | AATTAATACGACTCACTATAggatgcggttatctgtgctgGTTTTAGAGCTAGAAATAGC | CRISPR-Cas9, target-specific DNA oligo |
| dre-bmp3-gRNA2 oligo | AATTAATACGACTCACTATAggaatcccgcgctggtgtattGTTTTAGAGCTAGAAATAGC |  |
| bmp3_F | GAGTAGCCTACACCAAAGTGAC | CRISPR-Cas9, genotyping primers |
| bmp3_R | AGTTGAAGCGCAAAACGAAC |  |
| bmp3_insitu_F | TCCCCGATTGCTCCCGTCGT | *In situ* hybridization primers |
| bmp3_insitu_R | ATTCCGCTCACGTTGCCCCG |  |

Supplementary References

1 Parra, G., Bradnam, K. & Korf, I. CEGMA: a pipeline to accurately annotate core genes in eukaryotic genomes. *Bioinformatics* 23, 1061-1067, doi:10.1093/bioinformatics/btm071 (2007).

2 Simão, F. A., Waterhouse, R. M., Ioannidis, P., Kriventseva, E. V. & Zdobnov, E. M. BUSCO: assessing genome assembly and annotation completeness with single-copy orthologs. *Bioinformatics* 31, 3210-3212, doi:10.1093/bioinformatics/btv351 (2017).

3 Li, R. *et al.* The sequence and *de novo* assembly of the giant panda genome. *Nature* 463, 311, doi:10.1038/nature08696 (2009).

4 Xu, Z. & Wang, H. LTR_FINDER: an efficient tool for the prediction of full-length LTR retrotransposons. *Nucleic Acids Res.* 35, W265-W268, doi:10.1093/nar/gkm286 (2007).

5 Edgar, R. C. & Myers, E. W. PILER: identification and classification of genomic repeats. *Bioinformatics* 21, i152-158, doi:10.1093/bioinformatics/bti1003 (2005).

6 Price, A. L., Jones, N. C. & Pevzner, P. A. *De novo* identification of repeat families in large genomes. *Bioinformatics*, i351-358, doi:10.1093/bioinformatics/bti1018 (2005).

7 Wicker, T. *et al.* A unified classification system for eukaryotic transposable elements. *Nat. Rev. Genet.* 10, 276, doi:10.1038/nrg2165 (2007).

8 Jurka, J. *et al.* Repbase Update, a database of eukaryotic repetitive elements. *Cytogenet. Genome Res.* 110, 462-467, doi:10.1159/000084979 (2005).

9 Chen, N. Using RepeatMasker to identify repetitive elements in genomic sequences. *Curr. Protoc. Bioinformatics*, Chapter 4, Unit 4.10, doi:10.1002/0471250953.bi0410s05 (2004).

10 Gao, B. *et al.* The contribution of transposable elements to size variations between four teleost genomes. *Mobile DNA-UK* 7, 4, doi:10.1186/s13100-016-0059-7 (2016).

11 Stanke, M. & Waack, S. Gene prediction with a hidden Markov model and a new intron submodel. *Bioinformatics* 19, 215--225, doi:10.1093/bioinformatics/btg1080 (2003).

12 Majoros, W. H., Pertea, M. & Salzberg, S. L. TigrScan and GlimmerHMM: two open source ab initio eukaryotic gene-finders. *Bioinformatics* 20, 2878-2879, doi:10.1093/bioinformatics/bth315 (2004).

13 Korf, I. Gene finding in novel genomes. *BMC Bioinformatics* 5, 59, doi:10.1186/1471-2105-5-59 (2004).

14 Keilwagen, J. *et al.* Using intron position conservation for homology-based gene prediction. *Nucleic Acids Res.* 44, e89-e89, doi:10.1093/nar/gkw092 (2016).

15 Lin, Q. *et al.* Draft genome of the lined seahorse, *Hippocampus erectus*. *Gigascience* 6, 1-6, doi:10.1093/gigascience/gix030 (2017).

16 Tang, S., Lomsadze, A. & Borodovsky, M. Identification of protein coding regions in RNA transcripts. *Nucleic Acids Res.* 43, e78, doi:10.1093/nar/gkv227 (2015).

17 Mount, S. M., Hamilton, J. P., Haas, B. J., Campbell, M. A. & Robin, B. C. Comprehensive analysis of alternative splicing in rice and comparative analyses with Arabidopsis. *BMC Genomics* 7, 327, doi:10.1186/1471-2164-7-327 (2006).

18 Haas, B. J. *et al.* Automated eukaryotic gene structure annotation using EVidenceModeler and the Program to Assemble Spliced Alignments. *Genome Biol.* 9, R7, doi:10.1186/gb-2008-9-1-r7 (2008).

19 Boeckmann, B. *et al.* The Swiss-Prot knowledgebase and its supplement TREMBL in 2003. *Nucleic Acids Res.* 31, 365-370, doi:10.1093/nar/gkg095 (2003).

20 Marchlerbauer, A. *et al.* CDD: a Conserved Domain Database for the functional annotation of proteins. *Nucleic Acids Res.* 39, 225-229, doi:10.1093/nar/gkq1189 (2011).

21 Conesa, A., Terol, J. & Robles, M. Blast2GO: a universal tool for annotation, visualization and analysis in functional genomics research. *Bioinformatics* 21, 3674-3676, doi:10.1093/bioinformatics/bti610 (2005).

22 Tatusov, R. L. *et al.* The COG database: new developments in phylogenetic classification of proteins from complete genomes. *Nucleic Acids Res.* 29, 22-28, doi:10.1093/nar/29.1.22 (2001).

23 Kanehisa, M. & Goto, S. KEGG: Kyoto Encyclopedia of Genes and Genomes. *Nucleic Acids Res.* 27, 29-34, doi:10.1093/nar/28.1.27 (2000).

24 Vitturi, R., Carbone, P., Catalano, E. & Macaluso, M. Chromosome Polymorphism in *Gobius paganellus*, Linneo 1758 (Pisces, Gobiidae). *Biol. Bull.-US* 167, 658-668, doi:10.2307/1541417 (1984).

25 Howell, W. M. & Black, D. A. Controlled silver-staining of nucleolus organizer regions with a protective colloidal developer: a 1-step method. *Experientia* 36, 1014-1015, doi:10.1007/BF01953855 (1980).

26 Servant, N. *et al.* HiC-Pro: an optimized and flexible pipeline for Hi-C data processing. *Genome Biol.* 16, 259, doi:10.1186/s13059-015-0831-x (2015).

27 Korneliussen, T. S., Albrechtsen, A. & Nielsen, R. ANGSD: analysis of next generation sequencing data. *BMC Bioinformatics* 15, 356, doi:10.1186/s12859-014-0356-4 (2014).

28 Barrett, J. C., Fry, B., Maller, J. & Daly, M. J. Haploview: analysis and visualization of LD and haplotype maps. *Bioinformatics* 21, 263-265, doi:10.1093/bioinformatics/bth457 (2005).

29 Casey, S. P., Hall, H. J., Stanley, H. F. & Vincent, A. C. The origin and evolution of seahorses (genus *Hippocampus*): a phylogenetic study using the cytochrome b gene of mitochondrial DNA. *Mol. Phylogenet. Evol.* 30, 261-272 (2004).

30 Teske, P. R., Cherry, M. I. & Matthee, C. A. The evolutionary history of seahorses (Syngnathidae: *Hippocampus*): molecular data suggest a West Pacific origin and two invasions of the Atlantic Ocean. *Mol. Phylogenet. Evol.* 30, 273-286 (2004).

31 Hall, R. The palaeogeography of Sundaland and Wallacea since the Late Jurassic. *J. Limnol.* 72, 1-17, doi:10.4081/jlimnol.2013.s2.e1 (2013).

32 Srinivasan, M. S. & Sinha, D. K. Early Pliocene closing of the Indonesian Seaway: evidence from north-east Indian Ocean and Tropical Pacific deep sea cores. *J. Asian Earth Sci.* 16, 29-44, doi:10.1016/S0743-9547(97)00041-X (1998).

33 Butzin, M., Lohmann, G. & Bickert, T. Miocene ocean circulation inferred from marine carbon cycle modeling combined with benthic isotope records. *Paleoceanography* 26, PA1203, doi:10.1029/2009pa001901 (2011).

34 von der Heydt, A. & Dijkstra, H. A. Effect of ocean gateways on the global ocean circulation in the late Oligocene and early Miocene. *Paleoceanography* 21, PA1011, doi:10.1029/2005pa001149 (2006).

35 Adams, C. G., Gentry, A. W. & Whybrow, P. J. Dating the terminal Tethyan event. *Utrecht Micropaleontological Bulletins* 30, 273-298 (1983).

36 Zalohar, J., Hitij, T. & Kriznar, M. Two new species of seahorses (Syngnathidae, *Hippocampus*) from the Middle Miocene (Sarmatian) Coprolitic Horizon in Tunjice Hills, Slovenia: The oldest fossil record of seahorses. *Ann. Paleontol.* 95, 71-96, doi:10.1016/j.annpal.2009.03.002 (2009).

37 Boehm, J. T. *et al.* Marine dispersal and barriers drive Atlantic seahorse diversification. *J. Biogeogr.* 40, 1839-1849, doi:10.1111/jbi.12127 (2013).

38 Lunt, D., Valdes, P., Haywood, A. & Rutt, I. Closure of the Panama Seaway during the Pliocene: implications for climate and Northern Hemisphere glaciation. *Clim. Dynam.* 30, 1-18, doi:10.1007/s00382-007-0265-6 (2008).

39 Longo, S. J., Faircloth, B. C., Meyer, A., Westneat, M. W. & Wainwright, P. C. Phylogenomic analysis of a rapid radiation of misfit fishes (Syngnathiformes) using ultraconserved elements. *Mol. Phylogenet. Evol.* 113, 33-48, doi:10.1016/j.ympev.2017.05.002 (2017).

40 Kaneps, A. G. Gulf-Stream - Velocity Fluctuations during the Late Cenozoic. *Science* 204, 297-301, doi:10.1126/science.204.4390.297 (1979).

41 Wessel, P., Smith, W. H. F., Scharroo, R., Luis, J. & Wobbe, F. Generic Mapping Tools: improved version released. *EOS Trans. AGU*, **94**, 409–410, doi:10.1002/2013EO450001 (2013).
