# Supplementary material for: Genome sequences reveal global dispersal routes and suggest convergent genetic adaptations in seahorse evolution
Source: Nat Commun. 2021 Feb 17;12:1094. doi: 10.1038/s41467-021-21379-x (PMC7889852; doi:10.1038/s41467-021-21379-x)
Supplement: Supplementary file 3 — Reporting Summary [file 41467_2021_21379_MOESM3_ESM.pdf]

Corresponding author(s): Qiang Lin, Xuemei Lu, Axel Meyer, Byrappa Venkatesh

Last updated by author(s): Jan 19, 2021

Reporting Summary

Nature Research wishes to improve the reproducibility of the work that we publish. This form provides structure for consistency and transparency in reporting. For further information on Nature Research policies, see our Editorial Policies and the Editorial Policy Checklist.

Statistics

For all statistical analyses, confirm that the following items are present in the figure legend, table legend, main text, or Methods section.

n/a	Confirmed	
☐	☒	The exact sample size (*n*) for each experimental group/condition, given as a discrete number and unit of measurement
☐	☒	A statement on whether measurements were taken from distinct samples or whether the same sample was measured repeatedly
☐	☒	The statistical test(s) used AND whether they are one- or two-sided<br>*Only common tests should be described solely by name; describe more complex techniques in the Methods section.*
☒	☐	A description of all covariates tested
☐	☒	A description of any assumptions or corrections, such as tests of normality and adjustment for multiple comparisons
☐	☒	A full description of the statistical parameters including central tendency (e.g. means) or other basic estimates (e.g. regression coefficient) AND variation (e.g. standard deviation) or associated estimates of uncertainty (e.g. confidence intervals)
☐	☒	For null hypothesis testing, the test statistic (e.g. *F*, *t*, *r*) with confidence intervals, effect sizes, degrees of freedom and *P* value noted<br>*Give P values as exact values whenever suitable.*
☐	☒	For Bayesian analysis, information on the choice of priors and Markov chain Monte Carlo settings
☐	☒	For hierarchical and complex designs, identification of the appropriate level for tests and full reporting of outcomes
☒	☐	Estimates of effect sizes (e.g. Cohen's *d*, Pearson's *r*), indicating how they were calculated

Our web collection on statistics for biologists contains articles on many of the points above.

Software and code

Policy information about availability of computer code

Data collection	No software was used to collect the data.
Data analysis	Clustal Omega (v1.2.4), RAxML (v8), wLogDate python program, BAMM (v2.5), BAMMtools, WTDBG (https://github.com/ruanjue/wtdbg), ICE software, Juicer, Juicebox, BWA-MEM (v0.7.17), SAMtools (v1.6), FreeBayes (v9.9.2), ANNOVAR, PLINK (v1.9), MEGA7, EIGENSOFT (v6.1.4), ANGSD (v 0.924), prank (v.140603), ASTRAL-III (v5.6.1), RAxML (v8) , BEAST (v2.4), Tracer (v1.7.1), SPREAD (v1.0.6), G-PhoCS, PSMC, PAML, and R packages (vioplot, circlize) were employed to analysis the data. Other scripts are available at Figshare (https://figshare.com/s/bf4662c1b013687eab3e).

For manuscripts utilizing custom algorithms or software that are central to the research but not yet described in published literature, software must be made available to editors and reviewers. We strongly encourage code deposition in a community repository (e.g. GitHub). See the Nature Research guidelines for submitting code & software for further information.

Data

Policy information about availability of data

All manuscripts must include a data availability statement. This statement should provide the following information, where applicable:

- Accession codes, unique identifiers, or web links for publicly available datasets
- A list of figures that have associated raw data
- A description of any restrictions on data availability

All sequencing data generated in this project are available at NCBI under BioProjects PRJNA613175 (PacBio), PRJNA613176 (Hi-C) and PRJNA612146 (Re-sequencing). In addition, processed datasets including custom codes (Datasets 1-9) are available at figshare (https://figshare.com/s/bf4662c1b013687eab3e). Source data are provided with this paper as a source data file.

Field-specific reporting

Please select the one below that is the best fit for your research. If you are not sure, read the appropriate sections before making your selection.

- [x] Life sciences
- [ ] Behavioural & social sciences
- [ ] Ecological, evolutionary & environmental sciences

For a reference copy of the document with all sections, see nature.com/documents/nr-reporting-summary-flat.pdf

Life sciences study design

All studies must disclose on these points even when the disclosure is negative.

Sample size	358 samples corresponding to 21 seahorse species were chosen for whole-genome resequencing. These 358 specimens are widely distributed in shallow water of tropical and temperate ocean and represent the major lineages of the genus Hippocampus. These specimens are sufficient to study the genetic diversity, evolutionary history and independent evolution of bony spines. The sample size is the largest used to date.
Data exclusions	1. For genome and resequencing data, low-quality reads and adapter sequences were removed from the raw data. See more details in the Methods section and Supplementary information in the paper.<br>2. Non-neutral loci were excluded from the demographic analysis using G-PhoCS software according to the software instructions. These non-neutral loci include cluster SNVs, simple repeats, transposable elements, sequences with the depth more than twice of the mean depth of each species, CpGs, exons of protein-coding genes and non-coding RNAs.<br>3. H. camelopardalis with relatively low sequencing depth was excluded from positive selection analysis of the spiny trait using PAML.
Replication	For in-situ hybridization experiments, at least three replicates of relevant embryoic developmental stages of the seahorse were used. In the F2 mutant bmp3 fish, we observed a series of scale defects, such as decrements in scale numbers, rearrangements, and irregular shapes. The F2 bmp3 (+14) mutant fishes gave 4/29 fish with scale defects, whereas 3/31 had scale defects for F2 bmp3(-2) mutant fish.
Randomization	Seahorse samples were randomly collected.
Blinding	We did not have blinding design since this study did not include any clinical trials.

Reporting for specific materials, systems and methods

We require information from authors about some types of materials, experimental systems and methods used in many studies. Here, indicate whether each material, system or method listed is relevant to your study. If you are not sure if a list item applies to your research, read the appropriate section before selecting a response.

Materials & experimental systems

n/a	Involved in the study	
x		Antibodies
x		Eukaryotic cell lines
x		Palaeontology and archaeology
	x	Animals and other organisms
x		Human research participants
x		Clinical data
x		Dual use research of concern

Methods

n/a	Involved in the study	
x		ChIP-seq
x		Flow cytometry
x		MRI-based neuroimaging

Animals and other organisms

Policy information about studies involving animals; ARRIVE guidelines recommended for reporting animal research

Laboratory animals	Fathers of Hippocampus erectus embryos (undetermined sex) used for in situ hybridization were obtain from private breeders from the Netherlands and were kept and bred at the Helmholtz Centre for Ocean Research Kiel, Germany.<br>The transgenic zebrafish (Danio rerio) parents labeled with green fluorescent protein for osteoblast-specific transcription factor (Osterix GFP) were used in this experiment. These parents were cultured at 26-28 °C under a controlled light cycle (14 h light, 10 h dark) to induce spawning.
Wild animals	This study did not involve wild live animals.
Field-collected samples	There are no field-collected samples used in this study.
Ethics oversight	Sampling of H. erectus for in situ hybridization was conducted in lines with local ethics regulations for Schleswig-Holstein, Germany.<br>Knockout experiment of Danio rerio was performed in accordance with approved Institutional Animal Care and Use Committee protocols of the scientific ethic committee of the Huazhong Agricultural University (HZAUFI-2018-018).

Note that full information on the approval of the study protocol must also be provided in the manuscript.
