# Supplementary material for: Genome sequences reveal global dispersal routes and suggest convergent genetic adaptations in seahorse evolution
Source: Nat Commun. 2021 Feb 17;12:1094. doi: 10.1038/s41467-021-21379-x (PMC7889852; doi:10.1038/s41467-021-21379-x)
Supplement: Supplementary file 4 — Descriptions of Additional Supplementary Files [file 41467_2021_21379_MOESM4_ESM.pdf]

Description of Additional Supplementary Files

Supplementary Data 1

Description: Transposable element content in the *Hippocampus erectus* genome.

Supplementary Data 2

Description: Summary of seahorse accessions.

Supplementary Data 3

Description: The 37 genes potentially under positive selection with accelerated nonsynonymous/synonymous rate ratio (dN/dS) on the branches of spiny seahorse lineages compared to non-spiny lineages using CODEML program in PAML.

Supplementary Data 4

Description: McDonald and Kreitman test (MKT) for three pairwise spiny and non-spiny sister seahorse species.

Supplementary Data 5

Description: Sampling probability of genus within Syngnathidae.
