# Supplementary material for: Genome sequences reveal global dispersal routes and suggest convergent genetic adaptations in seahorse evolution
Source: Nat Commun. 2021 Feb 17;12:1094. doi: 10.1038/s41467-021-21379-x (PMC7889852; doi:10.1038/s41467-021-21379-x)
Supplement: Supplementary file 2 — Peer Review File [file 41467_2021_21379_MOESM2_ESM.pdf]

Reviewer #1 (Remarks to the Author):

Comments to the authors

This paper by Li and colleagues has much to offer. The authors report the de novo sequencing of a chromosome-level genome for Hippocampus erectus, in addition to resequencing of 358 individuals of 21 seahorse species. The manuscript shows inferred colonization routes of seahorses based on a calibrated phylogeny and indicates candidate SNPs for the convergent evolution of spines. The gene knockouts are outside of my expertise but I find the results very interesting. This powerful dataset is used to address interesting questions about the evolutionary history of the group in a well written and engaging manuscript.

I have questions regarding the calibration points of the phylogeny and the estimation of branch lengths that could possibly impact some of the results. The association of the development of spines with reefs needs more elaboration. Further, the diversification analysis (Extended Fig. 1) has some issues. These are detailed below and minor comments are listed below.

Fossil and biogeographic calibrations

The calibration of the phylogeny is a central result of the manuscript. The justifications of the placement of the fossils and the settings for the calibration densities therefore need to be explicit. Given the geologically relatively recent intervals that are being inferred here, even 1-2 million years of discrepancy may alter the inferred biogeographic history.

It is unclear where the cited value of 13 Ma for the root calibration and calibration 2 derives from. Zalohar et al. 2009 Annales de Paléontologie, which is cited in the manuscript as the source, gives the minimum bound for the Sarmatian as 11.6 Ma (Figure 2 and text in Zalohar). I believe this 11.6 Ma is the appropriate value to use as a minimum bound (offset of the lognormal distribution), accompanied by mean and standard deviations to allow the lognormal calibration density to extend further backwards.

Both the root calibration and calibration 3 do not include the actual calibration age in the 95% HPD interval. That is a considerable constraint for these nodes to be older than the fossil/biogeographic event. In my opinion, a better approach would be to establish an offset (i.e. the exact age of the fossil or biogeographic calibration), and then apply mean and standard deviation to the lognormal distribution to include justified ages in the past. At the moment, the values for offset, mean and standard deviations (as given in Suppl Result 13) are not justified.

The 95% HPD interval of calibration 2 (9.52-18.4 Ma) predates the minimum bound by the fossil by 2 million years (H. sarmaticus, 11.6 Ma). If I understand correctly, this calibration density was chosen because the fossil is assumed to lie along the branch of H. trimaculatus rather than the split of H. trimaculatus and its extant sister group. This may be ok but how were the values for mean and SD derived? What is the evidence to not allow the calibration density to extend to even younger ages? These choices impact the inferred ages.

This may be personal preference but given that a focus of the manuscript is the biogeographic inference, the inclusion of a biogeographic calibration constraint seems counterintuitive. Would it not be more interesting to infer the age of the trans-isthmian sister pair without an imposed constraint and see how it relates to the closure of the Panama Isthmus?

Branch lengths and calibration settings

Branch lengths: The phylogenetic analyses are appropriate given the size of this dataset. It is however not clear what branch lengths were used for biogeographic and PAML analyses. For the biogeographic analysis, the manuscript states "The topology and branch lengths of the species tree were used to reconstruct the geographic diversification" (L.472). Which branch lengths does this refer to? ASTRAL gives branch lengths in coalescent units; while it is conventional to use branch lengths representing substitution rates. The section on ASTRAL mentions under which model gene trees were inferred, but not if branch lengths were inferred on the ASTRAL species tree topology with some other method. If branch lengths in coalescent units were used, these analyses need to be redone.

Site model: It is unclear under which model substitution rates were inferred during StarBEAST analysis and I could not access the dryad data. Details on the substitution model(s), how they were obtained (e.g. ModelFinder, ModelTest-NG) and partitions (if applicable) need to be in the manuscript.

Clock model: Information on the clock model during StarBEAST analysis is missing. The choice of a strict or relaxed clock (or multiple clocks) will impact the outcome of the calibration analysis. (I note that a lognormal clock is mentioned for the biogeographic analysis.)

Diversification analysis

Extended Data Figure 1 on diversification rates is surely interesting but I have concerns about the analysis and the dataset. The manuscript used the FishTree dataset by Rabosky et al., which compiled available sequence data for fish, dated the resulting phylogeny and inferred diversification rates. It is an amazing resource but I am not convinced of the utility of the dataset at this relatively narrow scope of Syngnathidae and find it somewhat disappointing to not see more data for Syngnathidae included (see point 2-4).

1) The use of a global sampling fraction on this dataset seems inappropriate. Seahorses certainly enjoy more research efforts than other Syngnathidae. Therefore, the FishTreeOfLife well sampled for Hippocampus but not for other groups. Using a global sampling fraction of 0.3 may reflect the syngnathid-wide sampling fraction but it is considerable underestimation for seahorses (31 species are represented in the FishTreeofLife phylogeny, Lourie et al. Zootaxa recognize 41 species, hence 0.76 of seahorse species are represented in the FishTreeofLife!). This can be adjusted with BAMM's option to include sampling proportions for individual clades that should be more appropriate with this uneven sampling.
2) There are errors in the FishTreeofLife within Syngnathidae (as downloaded from https://fishtreeoflife.org/taxonomy/order/Syngnathiformes/). The following factors need consideration. E.g. Nerophis lumbriciformes and Cosmocampus elucens are within Syngnathus, one of the clades that is inferred to have high diversification rates. These could be misidentifications of Syngnathus spp but it begs the question if the analysis would return the same result if this was performed on a cleaned tree.
3) Syngnathus includes S. exilis and S. leptorhynchus, which were synonymized with S. californiensis because there is no genetic differentiation (Garcia et al. J Fish Biol). Removing these two extremely short branches could reduce the inferred heightened diversification rate in Syngnathus. There may also be differences in the seahorse clade compared to the phylogeny in this study.
4) To make a statement that seahorses have the greatest diversification rates within their family, one would wish for greater representation than currently presented. This would be possible by including the dataset of Hamilton et al. Mol Phylog Evol, which would allow to incorporate more diversity of syngnathids in addition to nuclear data.

Spines and association with reefs

Again, it seems intuitive that spines are defensive but it is not obvious why having spines would be more relevant on coral reefs rather than seagrass or kelp. Seahorses are cryptic on reefs and seagrass and kelp. Maybe the main text could briefly elaborate on this argument or tone down on this "key

adaptation to reef life".

On that note, Reference 15 (a handbook of seahorse identification) seems to be misplaced as a citation to support that spines "presence or absence of bony spines, which are an adaption against predators in coral reef communities15" (L.84) and "which are an effective defense against predators in coral reef communities where they live15" (L.174).

Specific comments

L.81: Reference 15 is handbook of species identification. There should be better references for male pregnancy, e.g. recently https://doi.org/10.1073/pnas.1916251117

L.115: Refers to Extended Data Fig. 5 which is the ASTRAL tree and not the tree based on SNPs as stated in the text.

L.434: How were the 2000 CDS selected from the 5400 CDS?

L. 436: Please give statistics (can be in supplementary material) on average length, and information content, missing data of alignments that were used for gene trees in ASTRAL.

L.436: The ASTRAL analysis included multiple individuals per species but Extended Data Fig. 5 only shows one terminal. Given that the figure shows terminal branch lengths, I believe that the multiind version of ASTRAL was used (Rabiee et al. Mol Phylogenet Evol) but this should be mentioned specifically.

L.445: It is not clear how many individuals were included in the starBEAST analysis. I assume these were assigned to their respective species in starBEAST?

L.445: Please give statistics (can be in supplementary material) on average length, and information content, missing data of alignments that were used for gene trees in ASTRAL and (further down) for starBEAST.

L.451, 454: Remove "the" before Hippocampus

L.456: Third calibration: "we set the divergence between H. reidi and H. ingens to a minimum of 2.8 Ma" (L. 457) is misleading because the 95% HPD interval does not include 2.8 Ma (3.07-4.64 Ma), which effectively excludes the minimum age.

L.534: Sentence seems incomplete.

L.536: Unclear how to build gene trees from the SNP dataset. Shouldn't that be gene trees for the CDS regions (including SNPs and invariant sites)?

L.791: The use of "basal" in phylogenetics is discouraged.
https://onlinelibrary.wiley.com/doi/full/10.1111/j.0307-6970.2004.00262.x

Fig 3d: This tree would be more informative if rooted.

Extended Fig. 8: Why does the cyan shade showing the last glacial period now include the Last Glacial Maximum with the sea level low stand? I believe the Last Glacial Period was from 115,000-11,700 years (including the Last Glacial maximum). The highlighted cyan period is just a small portion.

Reviewer #2 (Remarks to the Author):

The manuscript presents a new high-quality de novo genome assembly for Hippocampus erectus and, additionally, resequenced genomes for 358 specimens (21 spp) of the genus Hippocampus. This genus includes more than 46 species of seahorses that are distributed worldwide, hence the study sampled almost 50% of the diversity. The new data are used to infer a phylogeny for the 21 species, and a calibrated phylogeny is used to infer biogeographic scenarios to explain the current distribution. Additional analyses are presented in the supplementary information section to estimate genetic diversity metrics (among and within species), and to estimate dN/dS rates along the phylogeny and identify genes under selection. In situ hybridization experiments with embryos are used to show expression of the bone morphogenetic protein gene (bmp3) in H. erectus, presumably to gain insight into the development of convergent ‘spiny seahorse’ phenotypes.

The new data are compelling and the analyses are technically robust, supporting most of the conclusions presented in the paper. The problem that I see with this manuscript is the presentation, and that there is almost no acknowledgement of previous work published by others on the same study system. In fact, most of the conclusions of this study are not novel but confirm and minimally improve on previous results. The writing is underwhelming and propped by flashy terms and misuse of terminology. Some of the methods are not properly explained and the results are not compared with previous knowledge. The only reference to previous work (“Previous studies addressing the global colonization and diversification history of seahorses were limited in terms of genetic data and sampled species” in Lines 86-87) fails to explain what the limitations were and how the present study is an improvement. Much work has gone into producing this massive study and many analyses and results are buried in supplementary layers with poor exposure or adding any significant insights.

If the main focus of the study is phylogeny and biogeography of seahorses, the results presented provide somewhat increased resolution compared to previous studies due to the massive amount of new genetic data analyzed. Previous studies from the early 2000s based on many taxa but few molecular markers or a recent study based on fewer taxa but hundreds of UCE loci have produced congruent results and suggested similar biogeographic scenarios. The new data could have been leveraged, instead, to compare the efficiency of different molecular markers and phylogenetic methods, to draw interest from a broader readership of systematic biologists. The convergent evolution of spiny phenotypes has been supported in these previous studies, therefore this is not a new result. The insitu hybridization study falls short of documenting the role of any gene or developmental pathway implicated in convergent evolution beyond what was already known in general for bone morphogenesis.

I provide below several line-specific comments and a few observations on the methods.

Line 61: I object the use of “driver” in this context. Vicariance due to geological events may have influenced processes or patterns of diversification by spatially separating formerly continuous species ranges. They are not “drivers” in the sense of having a predetermined outcome towards which these systems may evolve (a “destination”). Please avoid using this trendy term without the proper context. Maybe just use “influence,” a more neutral term.

Line 63: “Colonization and adaption to new environments…” Do you mean “adaptation”?

Line 64: “..are major challenges during speciation and thus *it* might be accompanied by rapid phenotypic evolution.” Replace “it” by “they” since you are referring to two major challenges (colonization and adaptation).

Line 65: "However, many dispersing marine fish lineages did not evolve rapid ecomorphological divergence in the past, even after experiencing extreme extrinsic pressures."
I do not understand the meaning of this sentence, how does it connects to the previous sentence? Nor do I see a connection with the conclusions drawn by Bellwood et al's review on the evolution of fishes and coral reefs cited (Ref 9). What are "extrinsic pressures"? The previous sentence refers to speciation, and this one to dispersal. Is "phenotypic evolution" in the previous sentence equal to "ecomorphological divergence" in this one? Bellwood's conclusion was that the rapid increase in biodiversity among fishes in coral reefs in the last 5 MY was not matched by changes in ecosystem function. Please clarify the intent of this sentence.

Line 69: Developmental mechanisms, not "development" mechanisms.

Line 77: Please clarify, "diversification rates" refers to increase in number of species/lineages (speciation minus extinction) or to morphological diversification?

Lines 88-89: "… we studied different mechanisms that have facilitated the rapid global diversification of seahorses. Specifically, we inferred their demographic history and clarified the role of the Tethys Seaway during their diversification." How is "demographic history" a mechanism? Is the Thethys Seaway a "mechanism"? My understanding of this study is that the authors inferred a time-calibrated phylogeny (based on genome resequencing) for 21 species (out of ~46 in the genus, or almost 50%) and somehow mapped or interpreted this result against geological information to develop a plausible biogeographic scenario. This seems like a more honest description of the goals of the study.

Is the presence or absence of bony spines really one of the "most variable traits within the genus"? Figure 1 shows only 4 species that have spines, are there more than 4 spiny seahorse species in the genus? How variable is this character?

Line 115: "A species tree based on the genome-wide SNPs…" If this is a reference to the tree in Figure 2a, then the statement is inaccurate. According to the methods, this tree was inferred based on 2000 gene trees inferred with RAxML and used as input for ASTRAL-III, and then calibrated with BEAST2. Please clarify and make figure legend more explicit as well.

Please use 'sister-group relationship' statements to describe the tree. H. abdominalis does not branch "first" since it is the sister group to the rest of the species; thus, by definition both branch off at the same time. The rest of the species are divided into two clades. Please don't use "recover" to state the resolution obtained by your inference (avoid bad jargon).

Line 134: following north-eastward or north-westward currents towards the Thetis Sea?

Lines 167-168: "Interestingly, species with similar phenotypes were inferred as polyphyletic in the species tree…" There are two problems with this statement because (i) it implies that this is a new discovery when in fact previous phylogenetic analyses already have shown that spiny seahorses are not closely related (e.g. Teske et al Ref 17), and (ii) the qualifier "polyphyletic" cannot be applied to "phenotypes" but rather to a group (of taxa). The same terminological problem is found in lines 176-177 ("showed the four spiny seahorses as polyphyletic").

Line 173: what is a "a positive selection analysis"? This statement needs to be expanded significantly to explain what kind of analysis was performed.

Why is it necessary to argue that 37 gene trees (based on amino acid sequences of genes under selection) did not resolve the spiny seahorses in a monophyletic group? Reference to the species tree (Fig. 2a) would be enough to make this point, or is anything added by these gene tree analyses?

Lines 183-185: "..whole-mount in situ hybridizations demonstrate specific bmp3 expression domains

in seahorse spines during their early development (Fig. 3d).” In the context of this paragraph, this sentence seems to imply that spiny seahorses get their spines due to the early expression (among other genes) of bmp3. But Figure 3d shows in situ hybridization in H. erectus, which is not a ‘spiny seahorse’… and the blue stains (bmp3 expression) seem widespread in fins, abdominal area, and head of the whole mount shown in the figure. Therefore, the evidence provided by this experiment seems misrepresented (overinterpreted?) in the text, given that bone morphogenesis is occurring throughout the developing embryo. Therefore, it seems that this is not a novel insight into spine formation in seahorses.

Both insights provided by this study (biogeographic history and phenotypic convergence) are not new, since they have been reported elsewhere before. The last paragraph (lines 191-195) implies otherwise and does not acknowledge previous work by others.

Lines 428-435: the methods described here for the compilation of gene alignments is obscure. Phylogenetic analysis started by identifying a set of 5475 “single-copy orthologs” using a previously described method (published by some of the same authors, Ref. 11). Then, pairwise alignments were done for two species H. erectus and the outgroup S. scovelli, and “CDS sequences for 2000 orthologs with the highest Blast score were then extracted for each specimen based on the SNP dataset.” This last step is unclear and the validation of orthology completely missing. How were the alignments for each gene compiled? Processed data could not be retrieved from Dryad (doi:10.5061/dryad.547d7wm5b), as indicated in the manuscript (Line 629). Given the increasing standardization of molecular markers used for large scale phylogenetic studies using both UCE loci and exons, it would be reasonable to sample those loci for phylogenetic inference instead of or in addition to the 2000 loci defined by this similarity search.

Line 791: “…basal lineage” Lineages can never be basal. H. abdominalis is the sister-group to the rest of the taxa, avoid misuse of “basal.”

Reviewer #3 (Remarks to the Author):

The manuscript reports the high coverage sequencing and high continuity de novo assembly of the Hippocampus erectus sea horse genome, together with resequencing data from 21 other species sampled around the world. This immense dataset is analysed to estimate a scenario explaining the evolution, divergence and geographical dispersion of the many species found today. A specific section deals with the evolution of bony spines in 4 species. Because the trait seems to have appeared independently, the author perform an analysis of positive selection and eventually focus on one gene, bmp3, suggesting that it is one of the genetic determinants of this morphological adaptation.
The manuscript is relatively short, well written and clear. I am not a specialist in demographic histories and species diversification, but the genomic part represents a superb set of data and has been well exploited to generate completely new information of substantial interest to the community and beyond, as far as I can see. On the other hand I have a serious issue with the section on positive selection, which represents about 1/3 of the manuscript. Maybe some of these can be explained by the author’s wish to write a very compact text but I could not find satisfying answers in the extended and supplementary data. Importantly, the manuscript describes several datasets as being available through the Dryad repository under the accession doi:10.5061/dryad.547d7wm5b (line 629). However there must be a problem because this doi identifier is unknown to both the Dryad repository and the doi.org database. The datasets must available to the reviewers, especially regarding the issue of data outputs from the PAML and MKT tests for positive selection (see below).

Positive selection of bony spines:

- Have the p-values for the PAML analysis been adjusted for multiple testing? This is not indicated in the methods. The corresponding methods and supplementary data sections say that the PAML test for positive selection was performed “for each gene”, therefore presumably for about 20.000 genes, in addition to tests on several branches in each gene tree. So p-values should be adjusted for these multiple tests. Given that the lowest p-values provided in Supp Table 15 is 2.10-5, this adjustment would convert all p-values to non-significant levels, and obliterate the section on positive selection. If p-values have indeed been adjusted in table S15, I would like to see the original bmp3 PAML output files, with the uncorrected p-values in the author’s response to reviews.
- I do not understand the data in Supp Table S15. According to the main text, these are the results of some of the many tests of positive selection, but which? Careful reading of the methods shows that these 37 candidate genes are the output of the PAML analysis (line 175). Please indicate this in the header of the table and please provide the branch/species for which the p-values are indicated.

- In figure 3a (left) please indicate in the caption what gene(s) where used to derive the dN/dS values and the MKT test results. It might be assumed that we are looking at the bmp3 gene family but this is not indicated. Please give the units for the 0.002 scale bar.
- Please explain the dN/dS value of 602.702 for the H. histrix branch. Is this extraordinary value well supported by the underlying data?
- Figure 3b shows a broad distribution of dN/dS values for the bmp3 gene in 4 spiny species. I understand that each species has one bmp3 gene, so there are 4 data points. Then, where does the broad and smooth distribution come from?
- Line 181, “…show that bmp3 evolved under natural selection” is ambiguous. It could be argued that all genes evolved and continue evolving under natural selection, which is an epistemological framework rather than a mode of molecular evolution. Maybe “… evolved under positive selection” would be more appropriate, especially with a rough time window, like “recently” or “in the last xx My” and with an indication of which lineages.

Minor points:

Figure 1a.
- For clarity, I would suggest reversing the legend of the gradient of nucleotide diversity, with high values at the top and low values at the bottom.
- The precise location of the different sampling areas are designated by a “pin” symbol that have different colours, but the meaning of the colours is not indicated. After a while we understand that it is related to the subtrees in Figure 1b.
Figure 3a.
- For the bmp3 gene comparison the caption of the figure says that “… polymorphic and fixed substitutions in spiny seahorses are indicated with red and green circles, respectively.” But conserved sites are actually in light green and polymorphisms/substitutions are in red and dark green (which almost looks like blue). I would strongly suggest avoiding the two shades of green.

Response to Reviewer Comments

Reviewer #1

This paper by Li and colleagues has much to offer. The authors report the de novo sequencing of a chromosome-level genome for Hippocampus erectus, in addition to resequencing of 358 individuals of 21 seahorse species. The manuscript shows inferred colonization routes of seahorses based on a calibrated phylogeny and indicates candidate SNPs for the convergent evolution of spines. The gene knockouts are outside of my expertise but I find the results very interesting. This powerful dataset is used to address interesting questions about the evolutionary history of the group in a well written and engaging manuscript.

I have questions regarding the calibration points of the phylogeny and the estimation of branch lengths that could possibly impact some of the results. The association of the development of spines with reefs needs more elaboration. Further, the diversification analysis (Extended Fig. 1) has some issues. These are detailed below and minor comments are listed below.

Response: We appreciate this reviewer comments. We have re-analyzed the data following all her/his suggestions. We agree that the association of spines and coral reefs is not very well supported and removed this from the current version.

Fossil and biogeographic calibrations

The calibration of the phylogeny is a central result of the manuscript. The justifications of the placement of the fossils and the settings for the calibration densities therefore need to be explicit. Given the geologically relatively recent intervals that are being inferred here, even 1-2 million years of discrepancy may alter the inferred biogeographic history.

It is unclear where the cited value of 13 Ma for the root calibration and calibration 2 derives from. Zalohar et al. 2009 Annales de Paléontologie, which is cited in the manuscript as the source, gives the minimum bound for the Sarmatian as 11.6 Ma (Figure 2 and text in Zalohar). I believe this 11.6 Ma is the appropriate value to use as a minimum bound (offset of the lognormal distribution), accompanied by mean and standard deviations to allow the lognormal calibration density to extend further backwards.

Response: Originally, we used the beginning of Samartian age as calibration point (13 Ma) instead of its end (11.6 Ma) as suggested by this reviewer. We understand this reviewer’s comment and in this new version we have estimated the divergence times using the value 11.6 Ma as offset in the lognormal distribution. The new results show slightly younger origins for nodes

(a few thousand years); however, they are very similar to our original results. Thus, the results remain largely unchanged in the current version.
We modified Figure 2a (main ms), Extended Data Figure 6, Supplementary Table S13.

Reviewer 1: Both the root calibration and calibration 3 do not include the actual calibration age in the 95% HPD interval. That is a considerable constraint for these nodes to be older than the fossil/biogeographic event. In my opinion, a better approach would be to establish an offset (i.e. the exact age of the fossil or biogeographic calibration), and then apply mean and standard deviation to the lognormal distribution to include justified ages in the past. At the moment, the values for offset, mean and standard deviations (as given in Suppl Result 13) are not justified.

Response: We understand this reviewer's concern. In this new version we have included further explanations about the priors selection in Materials and Methods (Lines 467-488), to make our decisions clear. On the one hand, prior 1 (*Hippocampus* origin) is expected to have happened before *H. samarticus* (>11.6 Ma) (Zalohar et al., 2009, *Annales de Paleontologie*), since this fossil has been recognized as a sister species of the derived *H. trimaculatus*. An earlier origin is also consistent with previous estimations (Teske et al., 2007, *BMC Evol. Biol.* and 2009 *Biol. Letters*; Rabosky et al., 2018, *Nature*). In addition, please note that even when using a different prior than the original version (offset = 11.6 Ma instead of 13 Ma), starBEAST still converges to similar times as the most likely estimates, which is expected if priors do not influence the posterior significantly. Finally, we would like to mention that J. Zalohar approved our calibrations involving *H. samarticus* (prior 1 and 2) by email.

Similar logic applies to prior 3 (divergence between *H. ingens* and *H. reidi*). This prior assumes that the split of *H. ingens* had to have happened when the Panama Isthmus closure was completed as the latest (minimum bound). However, it is likely that gene flow was gradually reduced deeper in time. O'Dea et al. (2016, *Sci. Adv.*) suggest that connection between Atlantic-Pacific Oceans allowing gene flow likely existed until 3.2 Ma. Here, we relaxed this prior to reach 3.07-4.64 Ma within the 95% HDP. Wide priors allow to model uncertainty, making our analysis more conservative. However, note that values outside of the 95% HPD are possible and only constrained below the offset value.

Reviewer 1: The 95% HPD interval of calibration 2 (9.52-18.4 Ma) predates the minimum bound by the fossil by 2 million years (H. sarmaticus, 11.6 Ma). If I understand correctly, this calibration density was chosen because the fossil is assumed to lie along the branch of H. trimaculatus rather than the split of H. trimaculatus and its extant sister group. This may be ok but how were the values for mean and SD derived? What is the evidence to not allow the calibration density to extend to even younger ages? These choices impact the inferred ages.

Response: This is correct. In a conservative methodology, we allow the *H. samarticus* fossil to lie along the branch of *H. trimaculatus*. This prior allows us to cover with higher probability the complete Middle Miocene upper bound (16 Ma) and also allows a more recent split of *H. trimaculatus* to the Late Miocene (~9 Ma). However, note that younger ages are still allowed in this prior (offset = 0). We added this information to Materials and Methods (Lines 474-478).

Reviewer 1: This may be personal preference but given that a focus of the manuscript is the biogeographic inference, the inclusion of a biogeographic calibration constraint seems counterintuitive. Would it not be more interesting to infer the age of the trans-isthmian sister pair without an imposed constraint and see how it relates to the closure of the Panama Isthmus?

Response: Thank you for this suggestion. This is a tradeoff between number of informative calibrations (which are useful data) and questions to test. Note that we are only constraining the minimum bound to the final closure of the Panama Isthmus (an obvious gene flow barrier). Deeper splits are allowed in our prior. In other words, we are assuming that the split has necessarily happened at least 2.8 Ma (vicariance), but we are still testing how long before it happened. Given all that, we decided to keep this calibration. This same calibration was repeatedly used before to study seahorses (e.g. Teske et al., 2007, *BMC Evol. Biol.* and 2009, *Biol. Letters*), as well as many other marine taxa (reviewed by O'Dea et al., 2016, *Sci. Adv.*).

Reviewer 1: Branch lengths and calibration settings
Branch lengths: The phylogenetic analyses are appropriate given the size of this dataset. It is however not clear what branch lengths were used for biogeographic and PAML analyses. For the biogeographic analysis, the manuscript states "The topology and branch lengths of the species tree were used to reconstruct the geographic diversification" (L.472). Which branch lengths does this refer to? ASTRAL gives branch lengths in coalescent units; while it is conventional to use branch lengths representing substitution rates. The section on ASTRAL mentions under which model gene trees were inferred, but not if branch lengths were inferred on the ASTRAL species tree topology with some other method. If branch lengths in coalescent units were used, these analyses need to be redone.

Response: This was clarified in the Materials and Methods section (Lines 498 and 557). The topology and branch lengths (divergence times) of the species tree were used for biogeographic analyses while the species tree topology without branch length was used for PAML analyses. Note that PAML can take branch length, but they are optional. If given, it will only use them as starting values for maximum likelihood iterations (see PAML documentation, pp 15 "Tree file format and representations of tree topology").

Reviewer 1: Site model: It is unclear under which model substitution rates were inferred during

StarBEAST analysis and I could not access the dryad data. Details on the substitution model(s), how they were obtained (e.g. ModelFinder, ModelTest-NG) and partitions (if applicable) need to be in the manuscript.
Clock model: Information on the clock model during StarBEAST analysis is missing. The choice of a strict or relaxed clock (or multiple clocks) will impact the outcome of the calibration analysis. (I note that a lognormal clock is mentioned for the biogeographic analysis.)

Response: We used simplest models for substitution (JC69) and molecular clocks (strict clock). However, substitution models and molecular clocks were set as unlinked among loci. This information was now added to Material and Methods (Lines 489-490). We have tried more complex models at the beginning of our analyses, however, reaching convergence was not possible in a realistic time frame (we discarded analyses after 2 months). Note also that our analysis for divergence time estimation took ~4 weeks on the University of Konstanz computer cluster (8 threads).

We are very sorry to have forgotten to give the temporary link of our dataset submitted to Dryad (https://datadryad.org/stash/share/h-IowEOERZjjckkHB9TyJ0X3_1wKTZnbuILDWe4qLEk). in the previous submission it and the DOI (doi:10.5061/dryad.547d7wm5b) will be available once our manuscript is accepted.

Reviewer 1: Diversification analysis
Extended Data Figure 1 on diversification rates is surely interesting but I have concerns about the analysis and the dataset. The manuscript used the FishTree dataset by Rabosky et al., which compiled available sequence data for fish, dated the resulting phylogeny and inferred diversification rates. It is an amazing resource but I am not convinced of the utility of the dataset at this relatively narrow scope of Syngnathidae and find it somewhat disappointing to not see more data for Syngnathidae included (see point 2-4).

1) The use of a global sampling fraction on this dataset seems inappropriate. Seahorses certainly enjoy more research efforts than other Syngnathidae. Therefore, the FishTreeOfLife well sampled for Hippocampus but not for other groups. Using a global sampling fraction of 0.3 may reflect the syngnathid-wide sampling fraction but it is considerable underestimation for seahorses (31 species are represented in the FishTreeofLife phylogeny, Lourie et al. Zootaxa recognize 41 species, hence 0.76 of seahorse species are represented in the FishTreeofLife!). This can be adjusted with BAMM's option to include sampling proportions for individual clades that should be more appropriate with this uneven sampling.

Response: Thank you for the suggestion. We accounted for the revised manuscript for non-random incomplete taxon sampling in BAMM, using the sample probabilities per genus (Supplementary Result 18).

Reviewer 1: 2) There are errors in the FishTreeofLife within Syngnathidae (as downloaded from https://fishtreeoflife.org/taxonomy/order/Syngnathiformes/). The following factors need consideration. E.g. Nerophis lumbriciformes and Cosmocampus elucens are within Syngnathus, one of the clades that is inferred to have high diversification rates. These could be misidentifications of Syngnathus spp but it begs the question if the analysis would return the same result if this was performed on a cleaned tree.

Response: Accepted. We have removed species that may be misidentifications of the genus *Syngnathus* from the tree as suggested.

Reviewer 1: 3) Syngnathus includes S. exilis and S. leptorhynchus, which were synonymized with S. californiensis because there is no genetic differentiation (Garcia et al. J Fish Biol). Removing these two extremely short branches could reduce the inferred heightened diversification rate in Syngnathus. There may also be differences in the seahorse clade compared to the phylogeny in this study.

Response: Done. We excluded *S. exilis* and *S. leptorhynchus* species.

Reviewer 1: 4) To make a statement that seahorses have the greatest diversification rates within their family, one would wish for greater representation than currently presented. This would be possible by including the dataset of Hamilton et al. Mol Phylog Evol, which would allow to incorporate more diversity of syngnathids in addition to nuclear data.

Response: Accepted. We have downloaded the above mentioned DNA sequences (Hamilton et al., 2016, *Mol. Phylog. Evol*) and combined them with the original sequences by Rabosky et al. (2018) (19 loci in total, datasets available on Dryad: Dataset 1). The new tree includes a total of 138 species of the family Syngnathidae and one outgroup. All details were incorporated into the Materials and Methods section “Diversification rate estimation in the Syngnathidae family” (Lines 348-361). Similar acceleration was also found in *Hippocampus* and we’ve substituted Extended Fig. 1 with a new one.

Reviewer 1:Spines and association with reefs
Again, it seems intuitive that spines are defensive but it is not obvious why having spines would be more relevant on coral reefs rather than seagrass or kelp. Seahorses are cryptic on reefs and seagrass and kelp. Maybe the main text could briefly elaborate on this argument or tone down on

this “key adaptation to reef life”. On that note, Reference 15 (a handbook of seahorse identification) seems to be misplaced as a citation to support that spines “presence or absence of bony spines, which are an adaption against predators in coral reef communities15” (L.84) and “which are an effective defense against predators in coral reef communities where they live15” (L.174).

Response: We agree and have generally removed the association of spines and coral reefs from the manuscript and focused on the adaptation against predators of spine traits (e.g. Lines 60 and 90).

Reviewer 1: Specific comments
L.81: Reference 15 is handbook of species identification. There should be better references for male pregnancy, e.g. recently https://doi.org/10.1073/pnas.1916251117

Response: Accepted. We agree and have replaced the reference (Line 88).

Reviewer 1: L.115: Refers to Extended Data Fig. 5 which is the ASTRAL tree and not the tree based on SNPs as stated in the text.

Response: Corrected (Line 138).

Reviewer 1: L.434: How were the 2000 CDS selected from the 5400 CDS?

Response: We randomly selected 2,000 CDS/loci. This is explained in the Materials and Methods, and we now clarified this in the main text (Line 449).

Reviewer 1: L. 436: Please give statistics (can be in supplementary material) on average length, and information content, missing data of alignments that were used for gene trees in ASTRAL.

Response: Done. We added this information to Materials and Methods (Lines 454-456). Selected loci have an average length of 1,548 (± 1,325), average segregating sites of 18% (± 4%) and average missing data 1%.

Reviewer 1: L.436: The ASTRAL analysis included multiple individuals per species but Extended Data Fig. 5 only shows one terminal. Given that the figure shows terminal branch lengths, I believe that the multiind version of ASTRAL was used (Rabiee et al. Mol Phylogenet Evol) but this should be mentioned specifically.

Response: This is correct. We have added Rabiee et al. 2019 reference to Materials and Methods (Line 452).

Reviewer 1: L.445: It is not clear how many individuals were included in the starBEAST analysis. I assume these were assigned to their respective species in starBEAST?

Response: We have randomly sampled 100 CDS/loci from the same 2,000 matrices used in the ASTRAL analysis, thus we include one to five individuals per species (103 individuals in total). We now included this information in Materials and Methods (Lines 461-465).

Reviewer 1: L.445: Please give statistics (can be in supplementary material) on average length, and information content, missing data of alignments that were used for gene trees in ASTRAL and (further down) for starBEAST.

Response: Done. We randomly selected 2,000 loci from 5,475 orthologs to conduct ASTRAL analysis. These 2,000 loci have an average length of 1,548bp (± 1,325bp), average segregating sites of 18% (± 4%) and average missing data 1%. We then randomly selected 100 loci from these 2,000 loci for starBEAST analysis and these 100 loci have an average length of 1579bp (± 1060), average segregating sites of 18% (± 4%) and 1% missing data in average. We included this information in Materials and Methods (Lines 454-456 and 464-465).

Reviewer 1: L.451, 454: Remove "the" before Hippocampus

Response: Done (Lines 468 and 471).

Reviewer 1: L.456: Third calibration: "we set the divergence between H. reidi and H. ingens to a minimum of 2.8 Ma" (L. 457) is misleading because the 95% HPD interval does not include 2.8 Ma (3.07-4.64 Ma), which effectively excludes the minimum age.

Response: The offset 2.8 Ma represents the minimum possible age for vicariance. Below this value, probability becomes zero. However, smaller values than 3.07 Ma are allowed with low probability. As mentioned above, O'Dea et al. (2016) propose that gene flow likely started to decrease 3.2 Ma, according to many other marine taxa. We have included this information to the Materials and Methods (Lines 478-488).

Reviewer 1: L.534: Sentence seems incomplete.

Response: We have corrected it (Lines 549-550).

Reviewer 1: L.536: Unclear how to build gene trees from the SNP dataset. Shouldn't that be gene trees for the CDS regions (including SNPs and invariant sites)?

Response: This was a mistake in the text and we have corrected it. The reviewer is correct that CDS sequences of all genes were constructed using both SNPs and invariant sites (Lines 553-554).

Reviewer 1: L.791: The use of "basal" in phylogenetics is discouraged. https://onlinelibrary.wiley.com/doi/full/10.1111/j.0307-6970.2004.00262.x

Response: We have removed this term (Lines 941-942).

Reviewer 1: Fig 3d: This tree would be more informative if rooted.

Response: This analysis was performed only with the ingroup taxa, thus we unfortunately do not have an outgroup for rooting this gene tree.

Reviewer 1: Extended Fig. 8: Why does the cyan shade showing the last glacial period now include the Last Glacial Maximum with the sea level low stand? I believe the Last Glacial Period was from 115,000-11,700 years (including the Last Glacial maximum). The highlighted cyan period is just a small portion.

Response: Thank you for spotting this error in our caption. The cyan shade should indicate the last glacial maximum, which we corrected now. We also shifted the last global glacial maximum shade slightly to correspond to Clark et al. 2009 (26500 years before present to 19000 years before present) (Fig. 3b,c of the present version).

Reviewer #2:

The manuscript presents a new high-quality de novo genome assembly for Hippocampus erectus and, additionally, resequenced genomes for 358 specimens (21 spp) of the genus Hippocampus. This genus includes more than 46 species of seahorses that are distributed worldwide, hence the study sampled almost 50% of the diversity. The new data are used to infer a phylogeny for the 21 species, and a calibrated phylogeny is used to infer biogeographic scenarios to explain the current distribution. Additional analyses are presented in the supplementary information section to estimate genetic diversity metrics (among and within species), and to estimate dN/dS rates along the phylogeny and identify genes under selection. In situ hybridization experiments with embryos are used to show expression of the bone morphogenetic protein gene (bmp3) in H. erectus, presumably to gain insight into the development of convergent 'spiny seahorse' phenotypes.

The new data are compelling and the analyses are technically robust, supporting most of the conclusions presented in the paper. The problem that I see with this manuscript is the presentation, and that there is almost no acknowledgement of previous work published by others on the same study system. In fact, most of the conclusions of this study are not novel but confirm and minimally improve on previous results. The writing is underwhelming and propped by flashy terms and misuse of terminology. Some of the methods are not properly explained and the results are not compared with previous knowledge. The only reference to previous work ("Previous studies addressing the global colonization and diversification history of seahorses were limited in terms of genetic data and sampled species" in Lines 86-87) fails to explain what the limitations were and how the present study is an improvement. Much work has gone into producing this massive study and many analyses and results are buried in supplementary layers with poor exposure or adding any significant insights.

Response: We can see that this reviewer has the impression that our previous manuscript did not acknowledge previous work on this subject sufficiently. We now discuss previous work more extensively and, acknowledge previous findings more prominently, moved some additional findings from the supplements to the main text and highlight the specific novelties of our findings more clearly. However, despite the very important work of previous studies, unfortunately, we cannot acknowledge all previous studies on this topic comprehensively but have to limit ourselves to only refer to those appearing to us most central in this research, as we already exceed the number of references allowed by the journal.

Reviewer 2: If the main focus of the study is phylogeny and biogeography of seahorses, the results presented provide somewhat increased resolution compared to previous studies due to the massive amount of new genetic data analyzed. Previous studies from the early 2000s based on many taxa but few molecular markers or a recent study based on fewer taxa but hundreds of

UCE loci have produced congruent results and suggested similar biogeographic scenarios. The new data could have been leveraged, instead, to compare the efficiency of different molecular markers and phylogenetic methods, to draw interest from a broader readership of systematic biologists. The convergent evolution of spiny phenotypes has been supported in these previous studies, therefore this is not a new result. The insitu hybridization study falls short of documenting the role of any gene or developmental pathway implicated in convergent evolution beyond what was already known in general for bone morphogenesis.

Response: We apologize for not having made more clear what differentiates our study from prior knowledge. We now more clearly mention previous research and highlight how/why/where our study adds new insights specifically. As the reviewer points out, the diversification history of seahorses was studied before, however, here we are including whole genomes for several individuals for many new species, which makes our study the most robust analysis to approach the evolutionary history of the genus to date. Specifically, we highlight the following novel findings in our study:

- First *de novo* chromosome-level genome assembly of *Hippocampus erectus*.
- 358 re-sequenced genomes of 21 *Hippocampus* species.
- First time-calibrated coalescent-based species tree of the genus *Hippocampus*.
- First explicit diversification model applied to study *Hippocampus* diversification in space and time. Note that previous inferences of spatial diversification of seahorse were based on qualitative interpretations of phylogenetic trees.
- Convergent evolution of spines, due to independent molecular substitutions in the *bmp3* gene.
- The new version of this manuscript also includes the most comprehensive phylogenetic tree for the Syngnathidae family (138 species, 19 loci) to date and shows that *Hippocampus* evolves under the highest diversification rates in the family.

Reviewer 2: I provide below several line-specific comments and a few observations on the methods.

Line 61: I object the use of “driver” in this context. Vicariance due to geological events may have influenced processes or patterns of diversification by spatially separating formerly continuous species ranges. They are not “drivers” in the sense of having a predetermined outcome towards which these systems may evolve (a “destination”). Please avoid using this trendy term without the proper context. Maybe just use “influence,” a more neutral term.

Response: We agree and rephrased this sentence. We use “impact… patterns of marine diversification” (Line 68).

Reviewer 2: Line 63: “Colonization and adaption to new environments…” Do you mean “adaptation”?

Response: Thank you for spotting this typo.

Reviewer 2: Line 64: “..are major challenges during speciation and thus *it* might be accompanied by rapid phenotypic evolution.” Replace “it” by “they” since you are referring to two major challenges (colonization and adaptation).

Response: Done. Thank you for spotting this grammatical error.

Reviewer 2: Line 65: “However, many dispersing marine fish lineages did not evolve rapid ecomorphological divergence in the past, even after experiencing extreme extrinsic pressures.” I do not understand the meaning of this sentence, how does it connects to the previous sentence? Nor do I see a connection with the conclusions drawn by Bellwood et al’s review on the evolution of fishes and coral reefs cited (Ref 9). What are “extrinsic pressures”? The previous sentence refers to speciation, and this one to dispersal. Is “phenotypic evolution” in the previous sentence equal to “ecomorphological divergence” in this one? Bellwood’s conclusion was that the rapid increase in biodiversity among fishes in coral reefs in the last 5 MY was not matched by changes in ecosystem function. Please clarify the intent of this sentence.

Response: We re-wrote this paragraph for clarity and also replaced the reference (Lines 65-76). Thank you for spotting this mistake.

Reviewer 2: Line 69: Developmental mechanisms, not “development” mechanisms.

Response: Corrected (Line 75).

Reviewer 2: Line 77: Please clarify, “diversification rates” refers to increase in number of species/lineages (speciation minus extinction) or to morphological diversification?

Response: Diversification rates here refers to the difference between speciation and extinction rates. We’ve added this in Materials and Methods (Line 355).

Reviewer 2: Lines 88-89: “… we studied different mechanisms that have facilitated the rapid global diversification of seahorses. Specifically, we inferred their demographic history and clarified the role of the Tethys Seaway during their diversification.” How is “demographic history” a mechanism? Is the Thethys Seaway a “mechanism”? My understanding of this study is that the

authors inferred a time-calibrated phylogeny (based on genome resequencing) for 21 species (out of ~46 in the genus, or almost 50%) and somehow mapped or interpreted this result against geological information to develop a plausible biogeographic scenario. This seems like a more honest description of the goals of the study.

Response: We re-wrote this section following this reviewer recommendation (Lines 92-105).

Reviewer 2: Is the presence or absence of bony spines really one of the “most variable traits within the genus”? Figure 1 shows only 4 species that have spines, are there more than 4 spiny seahorse species in the genus? How variable is this character?

Response: The term “variable” was not ideal here. What we intended to refer to is the fact that many traits are quite variable among species, but also within species (e.g. body color, dermal appendages, etc.). Spines, in contrast, seem to be either present or absent in all adults of a given species. In the manuscript, we avoid this unclarity by using the word “eye-catching” instead of “variable” now (Line 104). Except the four species above, another two species (*H. jugumus* and *H. satomiae*) also have remarkable body spines, but these could unfortunately not be included in this study.

Reviewer 2: Line 115: “A species tree based on the genome-wide SNPs…” If this is a reference to the tree in Figure 2a, then the statement is inaccurate. According to the methods, this tree was inferred based on 2000 gene trees inferred with RAxML and used as input for ASTRAL-III, and then calibrated with BEAST2. Please clarify and make figure legend more explicit as well.

Response: Yes, the species tree was inferred based on 2,000 gene trees. We have corrected this (Line 138).

Reviewer 2: Please use ‘sister-group relationship’ statements to describe the tree. H. abdominalis does not branch “first” since it is the sister group to the rest of the species; thus, by definition both branch off at the same time. The rest of the species are divided into two clades. Please don’t use “recover” to state the resolution obtained by your inference (avoid bad jargon).

Response: We revised this in the manuscript now (Lines 138-140).

Reviewer 2: Line 134: following north-eastward or north-westward currents towards the Thetis Sea?

Response: Sorry for this writing error. We assumed its distribution being along the west-African coast and then an ocean north-westward ocean current presumably present ~15 Ma flowing

along this shoreline and into the Tethys would have facilitated their dispersal. In order to simplify the description, we used “north-westward” in the revised manuscript (Line 151).

Reviewer 2: Lines 167-168: “Interestingly, species with similar phenotypes were inferred as polyphyletic in the species tree…” There are two problems with this statement because (i) it implies that this is a new discovery when in fact previous phylogenetic analyses already have shown that spiny seahorses are not closely related (e.g. Teske et al Ref 17), and (ii) the qualifier “polyphyletic” cannot be applied to “phenotypes” but rather to a group (of taxa). The same terminological problem is found in lines 176-177 (“showed the four spiny seahorses as polyphyletic”).

Response:
(i) We generally agree that that we were not the first to find that spiny seahorses are not all closely related, regret to not have pointed this before and specifically mention this now with corresponding references (Line 251).
(ii) We now avoid the term polyphyletic in favor of “not closely related” when referring to the spiny phenotype (Line 265).

Reviewer 2: Line 173: what is a “a positive selection analysis”? This statement needs to be expanded significantly to explain what kind of analysis was performed.

Response: We should have been more specific before. Our positive selection analysis investigated the nonsynonymous/synonymous SNP rate ratio (d*N*/d*S*) on the branches of spiny vs. non-spiny seahorse lineages and identified genes in which significantly increased dN/dS ratios linked to spiny lineages were determined. It was conducted using the codeml program in PAML. We have expanded the statement of “a positive selection analysis” to specify what kind of analysis was performed (Lines 258-262).

Reviewer 2: Why is it necessary to argue that 37 gene trees (based on amino acid sequences of genes under selection) did not resolve the spiny seahorses in a monophyletic group? Reference to the species tree (Fig. 2a) would be enough to make this point, or is anything added by these gene tree analyses?

Response: We are sorry that this was not made clear in the main text. The species tree illustrates that spiny seahorses do not form monophyletic clade, but spines evolved repeatedly and convergently. We used the phylogenetic trees of the 37 genes showing signals of positive selection (see comment above on this topic) to illustrate that in no single gene tree the spiny seahorses are grouped as being monophyletic. This argues that all 37 genes have likely evolved

independently (and not in parallel) in response to natural selection to produce a spine phenotype. This information cannot be obtained by exclusively analysing the species tree.

Reviewer 2: Lines 183-185: "..whole-mount in situ hybridizations demonstrate specific bmp3 expression domains in seahorse spines during their early development (Fig. 3d)." In the context of this paragraph, this sentence seems to imply that spiny seahorses get their spines due to the early expression (among other genes) of bmp3. But Figure 3d shows in situ hybridization in H. erectus, which is not a 'spiny seahorse'… and the blue stains (bmp3 expression) seem widespread in fins, abdominal area, and head of the whole mount shown in the figure. Therefore, the evidence provided by this experiment seems misrepresented (overinterpreted?) in the text, given that bone morphogenesis is occurring throughout the developing embryo. Therefore, it seems that this is not a novel insight into spine formation in seahorses.

Response: We agree that our phrasing was unclear and we might have overstated the link of *bmp3* and the spine phenotype. What we meant to state (and now do more clearly) is that the spiny seahorses' derived *bmp3* may alter the generic spine development. To our knowledge all syngnathids (as observed in representatives from the genera *Nerophis*, *Doryrhamphus*, *Syngnathus* & *Hippocampus*) develop spine-like protrusions in early development but in most species these are lost soon after birth, potentially due to regulatory effects of *bmp3*, which is an osteoblast differentiation regulator in mammals. In spiny seahorses the derived form of this gene may have altered downstream regulatory interactions with other genes, which may contribute to spine growth for a longer time into development. We rewrote this section (Lines 233-284).

Reviewer 2: Both insights provided by this study (biogeographic history and phenotypic convergence) are not new, since they have been reported elsewhere before. The last paragraph (lines 191-195) implies otherwise and does not acknowledge previous work by others.

Response: While we agree that parts of our findings have been reported before (which we mention now more clearly), we disagree with the statement that our study would not provide new insights in the biogeographic history and phenotypic convergence of seahorses. As listed above, there are several novel insights in our study (aside from providing massive amounts of new genomic resources to the community). Firstly, concerning the biogeographic history of seahorses, many aspects of seahorses' biogeography remained uncertain to date, as different studies using different genetic markers and species found conflicting results. Our study uses the by far most comprehensive data set of the genus *Hippocampus* to produce the most reliable and one of the most comprehensive phylogenies available to date. No other study has yet interpreted the biogeography and diversification of seahorses on a global scale by highlighting comprehensively how tectonically induced vicariances and also associated changes in oceanic currents likely affected the seahorse clades' distribution patterns. We agree that many colonization routes of

individual clades that we identified have been suggested before at some point (and we acknowledge this now more in the main text), however, many of these remain controversially debated. Our study provides a much needed, well supported analysis, in which we confirm some previously proposed theories, while rejecting others, and proposing new ones. This is in our opinion a new and valuable insight.

Concerning the convergent evolution of spines, we generally agree that that we were not the first to find that spiny seahorses are not all closely related and specifically mention this now with corresponding references. However, more recent phylogenies did not address this issue. For example, Hamilton et al. 2016 did not have a single "spiny" species among sampled species and Longo et al. 2017 only had two "spiny" species (*H. barbouri* & *H. histrix*), which actually were arranged as sister clades due to a low number of sampled species. Hence, we feel that it is worth drawing attention to this convergence, also as our phylogeny is very well supported at all branches, which was not the case for some phylogenies produced in the earlier days of seahorse phylogenetics. Thus, while it is not new that prominent spines evolved convergently, our data is new in that the resolution and reliability of our phylogeny, due to the massive amount of data obtained from recent sequencing technologies, is by far exceeding that of previously published work. Finally, the role of *bmp3* gene in the spine formation (also other genes listed in the supplementary material) is a novel finding from our study.

Reviewer 2: Lines 428-435: the methods described here for the compilation of gene alignments is obscure. Phylogenetic analysis started by identifying a set of 5475 "single-copy orthologs" using a previously described method (published by some of the same authors, Ref. 11). Then, pairwise alignments were done for two species H. erectus and the outgroup S. scovelli, and "CDS sequences for 2000 orthologs with the highest Blast score were then extracted for each specimen based on the SNP dataset." This last step is unclear and the validation of orthology completely missing. How were the alignments for each gene compiled? Processed data could not be retrieved from Dryad (doi:10.5061/dryad.547d7wm5b), as indicated in the manuscript (Line 629). Given the increasing standardization of molecular markers used for large scale phylogenetic studies using both UCE loci and exons, it would be reasonable to sample those loci for phylogenetic inference instead of or in addition to the 2000 loci defined by this similarity search.

Response: We apologize for forgetting to provide the temporary link of our dataset submitted to Dryad (https://datadryad.org/stash/share/h-IowEOERZjjckkHB9TyJ0X3_1wKTZnbuILDWe4qLEk) and the DOI (doi:10.5061/dryad.547d7wm5b) will be available once our manuscript has been accepted.

Species trees are usually constructed using conserved sequences among species including orthologous genes, UCE loci and exons. As we had novel whole-genome sequences of 358

seahorses, we made use of this unprecedented dataset to construct the species tree by using the most conserved genes within Syngnathidae. 5,475 single-copy orthologous genes of three published genomes of this family (*S. scovelli*, *H. erectus* and *H. comes*) were identified using Treefam. We then randomly selected 2000 out of 5,475 genes to construct the species tree. Taken the genome of *H. erectus* as the reference, 2,000 CDS sequences of all re-sequenced seahorses were constructed using both SNP and invariant sites and sequence of *S. scovelli* was constructed based on pair-wise alignment between *S. scovelli* and *H. erectus*. Details for these 2,000 genes are listed above and also shown in Dryad (Dataset 3).

Line 791: "…basal lineage" Lineages can never be basal. H. abdominalis is the sister-group to the rest of the taxa, avoid misuse of "basal."

Response: We corrected this throughout the manuscript (e.g. Lines 941-942).

Reviewer #3

The manuscript reports the high coverage sequencing and high continuity de novo assembly of the Hippocampus erectus seahorse genome, together with resequencing data from 21 other species sampled around the world. This immense dataset is analysed to estimate a scenario explaining the evolution, divergence and geographical dispersion of the many species found today. A specific section deals with the evolution of bony spines in 4 species. Because the trait seems to have appeared independently, the author perform an analysis of positive selection and eventually focus on one gene, bmp3, suggesting that it is one of the genetic determinants of this morphological adaptation.

The manuscript is relatively short, well written and clear. I am not a specialist in demographic histories and species diversification, but the genomic part represents a superb set of data and has been well exploited to generate completely new information of substantial interest to the community and beyond, as far as I can see. On the other hand I have a serious issue with the section on positive selection, which represents about 1/3 of the manuscript. Maybe some of these can be explained by the author's wish to write a very compact text but I could not find satisfying answers in the extended and supplementary data. Importantly, the manuscript describes several datasets as being available through the Dryad repository under the accession doi:10.5061/dryad.547d7wm5b (line 629). However there must be a problem because this doi identifier is unknown to both the Dryad repository and the doi.org database. The datasets must available to the reviewers, especially regarding the issue of data outputs from the PAML and MKT tests for positive selection (see below).

Positive selection of bony spines:

- Have the p-values for the PAML analysis been adjusted for multiple testing? This is not indicated in the methods. The corresponding methods and supplementary data sections say that the PAML test for positive selection was performed "for each gene", therefore presumably for about 20.000 genes, in addition to tests on several branches in each gene tree. So p-values should be adjusted for these multiple tests. Given that the lowest p-values provided in Supp Table 15 is 2.10-5, this adjustment would convert all p-values to non-significant levels, and obliterate the section on positive selection. If p-values have indeed been adjusted in table S15, I would like to see the original bmp3 PAML output files, with the uncorrected p-values in the author's response to reviews.

Response: The assembled genome contains 20,137 protein-coding genes, and the adjusted *p* values after adopting Bonferroni correction would be insignificant for all the genes. However, no gene having significantly adjusted *p* values after applying stringent Bonferroni correction does not necessarily indicate that none of the genes are significantly associated with bony spines. Bonferroni correction is mainly used for constraint of Type I error, i.e. chance of false positives, in

contrast, it may lead to a very high rate of Type II error, i.e. chance of false negatives and thereby omit important genes that could be under potentially positive selection with accelerated dN/dS on the branches of spiny seahorse lineages compared to non-spiny lineages if only pursuing extremely low false positive. Balancing between controls of false positives and false negatives is predominantly determined according to specific purpose of the study. In our research, we try to exploit a set of genes potentially under positive selection with accelerated d*N*/d*S* on the branches of spiny seahorse lineages. Since none of the genes reach significant level for likelihood ratio test after Bonferroni correction, we adopted a relatively strict threshold of 0.001 for the original *p* values, and initially obtained 37 putative genes under positive selection with significant signals of accelerated d*N*/d*S* (Supplementary Result 16). To further constrain the false positives of the 37 putative genes, we subsequently performed canonical and generalized McDonald and Kreitman tests (MKT) between three pairwise sister species with divergent spiny and non-spiny features, which take advantage of the population sequence data (Supplementary Result 17). Afterwards, we also implemented the 'Free-ratio model' to estimate the variable d*N*/d*S* ratio on each phylogenetic branch based on the aligned codon sequences for each gene through the maximum likelihood method using CODEML in PAML (Dryad: Dataset 8). By integrating the results from the 3 above mentioned analyses, we found that there are 3 genes, namely *bmp3, tlk1,* and *map2k1*, showing both significance in PAML and MKT, and consistently presenting accelerated d*N*/d*S* from 'Free-ratio model' on the branches of spiny seahorse lineages in comparison with those of non-spiny lineages, especially with those of sister non-spiny lineages. Additionally, two of the genes (*bmp3* and *map2k1*) are all reasonably associated with bone development according to the functional annotation. Thereby, *bmp3* gene was further considered as a confident candidate for further experimental confirmation. We have added corresponding information in the revised manuscript (Lines 589-594).

Reviewer 3: - I do not understand the data in Supp Table S15. According to the main text, these are the results of some of the many tests of positive selection, but which? Careful reading of the methods shows that these 37 candidate genes are the output of the PAML analysis (Line 175). Please indicate this in the header of the table and please provide the branch/species for which the p-values are indicated.

Response: We are sorry for this being unclear in the earlier version of the manuscript. Present Supplementary Result 16 listed the 37 genes potentially under positive selection with accelerated nonsynonymous/synonymous rate ratio (d*N*/d*S*) on the branches of spiny seahorse lineages compared to non-spiny lineages using CODEML program in PAML. We performed both "One-ratio" and "Two-ratio" codon substitution models, where "One-ratio" model assumes the same d*N*/d*S* across all the branches in the phylogenic tree of seahorses, as the 'null hypothesis', and "Two-ratio" model presumes diverged d*N*/d*S* for the branches of spiny and non-spiny lineages, as 'alternative hypothesis'. Likelihood ratio tests were conducted to compare the above-mentioned

models by calculating the corresponding likelihoods, χ2 critical values, and *p* values for each gene. To make the table more easily understandable, we now added this necessary information including the specified header in the revised Supplementary Result 16.

Reviewer 3: - In figure 3a (left) please indicate in the caption what gene(s) where used to derive the dN/dS values and the MKT test results. It might be assumed that we are looking at the bmp3 gene family but this is not indicated. Please give the units for the 0.002 scale bar.

Response: Done. We added the *bmp3* gene in the caption of figure 3a. The scale bar of branch lengths are expressed in nucleotide substitutions of the species tree and it has been incorporated to the legend.

Reviewer 3: - Please explain the dN/dS value of 602.702 for the H. histrix branch. Is this extraordinary value well supported by the underlying data?

Response: We estimated the d*N*/d*S* ratio for each branch (including the d*N*/d*S* value of 602.702 for the *H. histrix* branch) in the phylogenetic tree by performing free-ratios model that assumes an independent d*N*/d*S* ratio for each branch using CODEML in PAML, which adopts a maximum likelihood method to estimate the d*N*/d*S* values based on the codon sequences of BMP3 in 20 species. We checked the underlying data for the calculation and found that the extraordinary value is due to the extremely low estimation of d*S* as 0.000003. Thank you for pointing this out.

Reviewer 3:- Figure 3b shows a broad distribution of dN/dS values for the bmp3 gene in 4 spiny species. I understand that each species has one bmp3 gene, so there are 4 data points. Then, where does the broad and smooth distribution come from?

Response: Yes, the broad distribution of d*N*/d*S* values for the *bmp3* gene showed in Figure 3b is derived from 4 d*N*/d*S* values from 4 spiny species. Since the fitted distribution from 4 values may be inaccurate, we changed the fitted distribution to histogram for the 4 data points.

Reviewer 3: - Line 181, "…show that bmp3 evolved under natural selection" is ambiguous. It could be argued that all genes evolved and continue evolving under natural selection, which is an epistemological framework rather than a mode of molecular evolution. Maybe "… evolved under positive selection" would be more appropriate, especially with a rough time window, like "recently" or "in the last xx My" and with an indication of which lineages.

Response: We agree that this was ambiguous. We changed this to "positive selection", which is what we meant to state (Line 262). We also added the time window when spines emerge in different lineages in the manuscript (Lines 254-256).

Reviewer 3: Minor points:

Figure 1a.
- For clarity, I would suggest reversing the legend of the gradient of nucleotide diversity, with high values at the top and low values at the bottom.

Response: Thanks, we have changed it.

Reviewer 3: - The precise locations of the different sampling areas are designated by a “pin” symbol that have different colours, but the meaning of the colours is not indicated. After a while we understand that it is related to the subtrees in Figure 1b.

Response: We have added the description in the figure caption (Line 302).

Reviewer 3: Figure 3a.
- For the bmp3 gene comparison the caption of the figure says that “… polymorphic and fixed substitutions in spiny seahorses are indicated with red and green circles, respectively.” But conserved sites are actually in light green and polymorphisms/substitutions are in red and dark green (which almost looks like blue). I would strongly suggest avoiding the two shades of green.

Response: Corrected.

Reviewer #1 (Remarks to the Author):

The authors have revised the manuscript with great care in order to respond to the different reviewer's comments. I am content and thankful for the careful responses to my questions.

My remaining suggestion concerns the discussion of Fig. 3b, which is too superficial for the complex patterns shown with PSMC. The hypothesized interactions between historical sea level and the effective population size are interesting. The plots in Fig. 3b show that Ne trajectories are complicated with many different patterns and that there is no uniform response to changes in sea levels. I understand that space is too limited to deep-dive into possible explanations for the different patterns but the conclusion of "a direct or indirect effect of seawater levels on Ne" (L.219) is too unspecific. The current description picks some species' Ne trajectories that arguably coincided with sea level highs or lows (even though the peaks are off by tens of thousands of years/generations), while other species' Ne trajectories that did not respond in an obvious way are not mentioned. Overall, a more balanced discussion would be helpful.

L. 220 and following claims that Ne maxima of H. hippocampus, H. casscsio, H. fuscus, H. subelongatus coincide with the last interglacial highstand. However, Fig 3b shows that the Ne maxima of these species postdate the yellow line. For example, the Ne for H. subelongatus reaches its maximum at ~75,000 years, which postdates the last interglacial peak (~115,000) by ~40,000 years, that is ~40,000 generations. From this pattern, the interpretation would be that population sizes kept increasing while sea level (and associated habitat) was decreasing again. Such a lag may be possible but should then be mentioned.

L. 226 and following states that the Ne maxima of H. ingens, H. spinosissimus, H. trimaculatus not coincide with the blue shaded area (which is supposed to highlight the LGM). However, their Ne maxima predate this period.
The distance between the Ne maximum and the LGM is likely even larger than currently visible on the figure because the LGM period appears to be misannotated. The LGM is by definition the period of the lowest sea level (caused by the maximum glacial extent). Therefore, the blue highlight should be further to the right where sea level was at -120 m. See also Figure 2 in Clark et al. 2009 Science (the source that the authors mention in the response letter) where the LGM overlapping with the lowest sea level stand.

Please also give the source of the sea level curve in Fig. 3c. It cannot be from Clark et al. 2009 because that dataset only extends back to 50,000 years while Fig. 3c goes back to 1 million years.

Minor points

L.121: How was monophyly of seahorses established? The referenced figure 1b nor Supplementary Results 8-11 do not include outgroups. The methods mention that the data for phylogenetic analysis were identified from 2 Hippocampus species and 1 Syngnathus species but with just one non-Hippocampus species, how can monophyly be established?

L.485: "to cover all possible older dates" is imprecise.

L.156: Typo in "spinnosissimus"

L.229: Capitalize "pacific"

Reviewer #2 (Remarks to the Author):

Thank you for revising the manuscript and addressing the concerns raised by my review. I feel that the revised version effectively addressed most issues raised. I did find a few typos and confusing language that could be improved in the final version.

Abstract has some typos: Line 48 "live" should be "life"; line 52: seahorses are expected to have diminished long range dispersal… not "at" long range dispersal…

Methods in lines 428-433 describe briefly how the NJ tree for 21 seahorse species was constructed. No outgroup taxa are mentioned. This is the NJ tree shown in figure 1b, based on SNPs for 358 seahorses. The main text refers to this figure in lines 121-123, stating: "our analysis identified all seahorse species as a monophyletic group in a neighbor-joining tree…"
What is the significance of mentioning that all seahorses are in a monophyletic group if this is the only possible result, given that no outgroup taxa were included in the analysis?

Line 192: "…ongoing gene flow from the East-African H. algiricus int the South American H . reidi… (Fig. 3a)." I believe that you really mean West-African H. algiricus or perhaps East-Atlantic H. algiricus.

Line 206: "These findings contrast more recent studies that suggested…" These seems to be a word missing here and "more" is not necessary. Perhaps "These findings contrast with recent studies…"

Reviewer #3 (Remarks to the Author):

I have read the author's response and I am generally happy with the changes, including for the main point regarding the tests for positive selection. In the first version of the manuscript it was not clear to me that the initial CODEML dN/dS test acted as a screening step to identify candidates that were then further tested using additional methods. The authors have a very low signal in the first screen (37 genes for 20 expected by chance) but I think that this is OK given that they focus on mainly 1 gene with strong support at the end. I would however refrain from saying (line 262) that the 37 genes "show significant signals of accelerated dN/dS in spiny seahorses ($p < 0.001$)" given that there is no adjustment for multiple testing. A Benjamini-Hochberg adjustment would cover this aspect. The authors could for example carry this out or just modify the text to remove the word "significant".

Response to the reviewers' comments

Reviewer #1 (Remarks to the Author):

The authors have revised the manuscript with great care in order to respond to the different reviewer's comments. I am content and thankful for the careful responses to my questions.

My remaining suggestion concerns the discussion of Fig. 3b, which is too superficial for the complex patterns shown with PSMC. The hypothesized interactions between historical sea level and the effective population size are interesting. The plots in Fig. 3b show that Ne trajectories are complicated with many different patterns and that there is no uniform response to changes in sea levels. I understand that space is too limited to deep-dive into possible explanations for the different patterns but the conclusion of "a direct or indirect effect of seawater levels on Ne" (L.219) is too unspecific. The current description picks some species' Ne trajectories that arguably coincided with sea level highs or lows (even though the peaks are off by tens of thousands of years/generations), while other species' Ne trajectories that did not respond in an obvious way are not mentioned. Overall, a more balanced discussion would be helpful.

Response: We agree that our discussion was quite biased towards the species whose *Nes* appeared to be linked to seawater levels. We now added to this by also mentioning other factors that could influence *Ne* dynamics, such as biome type or dispersal abilities (Lines 230-238).

L. 220 and following claims that Ne maxima of H. hippocampus, H. casscsio, H. fuscus, H. subelongatus coincide with the last interglacial highstand. However, Fig 3b shows that the Ne maxima of these species postdate the yellow line. For example, the Ne for H. subelongatus reaches its maximum at ~75,000 years, which postdates the last interglacial peak (~115,000) by ~40,000 years, that is ~40,000 generations. From this pattern, the interpretation would be that population sizes kept increasing while sea level (and associated habitat) was decreasing again. Such a lag may be possible but should then be mentioned.

Response: We agree that these local peaks in *Ne* are actually following the last interglacial peak and are more clear about this in the manuscript now (Line 222).

L. 226 and following states that the Ne maxima of H. ingens, H. spinosissimus, H. trimaculatus not coincide with the blue shaded area (which is supposed to highlight the LGM). However, their Ne maxima predate this period.
The distance between the Ne maximum and the LGM is likely even larger than currently visible on the figure because the LGM period appears to be misannotated. The LGM is by definition the period of the lowest sea level (caused by the maximum glacial extent). Therefore, the blue highlight should be further to the right where sea level was at -120 m. See also Figure 2 in Clark et al. 2009 Science (the source that the authors mention in the response letter) where the LGM overlapping with the lowest sea level stand.

Please also give the source of the sea level curve in Fig. 3c. It cannot be from Clark et al. 2009 because that dataset only extends back to 50,000 years while Fig. 3c goes back to 1 million years.

Response: The fact that the cyan shade (last LGM) and seawater levels do/did not align perfectly came about as two different papers were used to obtain seawater levels (Miller et al., 2005, as cited (ref. 33)) and the time of the last LGM (Clark et al., 2009). Unfortunately, the more recent paper did not include the long-term seawater level record we wanted to link *Ne* sizes to, as correctly suggested by the reviewer, which is why we ended up with this slight discrepancy. We also noted that discrepancies in the precise timing of the LGM are common among papers, probably as different datasets from different parts of the world were analyzed. To avoid this confusion (and because a discussion of the effect of seawater-level on seahorse *Nes* on a regional scale is beyond the focus of this study), we moved the cyan shading to reflect the time interval of the lowest seawater levels according to Miller et al.

Minor points

L.121: How was monophyly of seahorses established? The referenced figure 1b nor Supplementary Results 8-11 do not include outgroups. The methods mention that the data for phylogenetic analysis were identified from 2 Hippocampus species and 1 Syngnathus species but with just one non-Hippocampus species, how can monophyly be established?

Response: Here we referred to each species monophyly (not the genus), as recovered in the NJ tree. We have re-phased this sentence for clarification: “Our analysis identified **each** seahorse species as a monophyletic group in a neighbor-joining tree” (Line 121).

L.485: “to cover all possible older dates” is imprecise.

Response: Agreed. We re-phrased as follows: “(…)for this study we used a conservative prior by setting the minimal possible divergence time between these lineages to 2.8 Ma and also allowed the hyperprior to cover older dates, with 95% HPD: 3.07-4.64 Ma, given that O’Dea et al. suggested that a connection between the Atlantic and Pacific Oceans allowing gene flow likely existed until 3.2 Ma (gradually reduced in time).” (Lines 491-495)

L.156: Typo in “spinnosissimus”

Response: We have corrected this spelling error (Line 229).

L.229: Capitalize “pacific”

Response: Revised (Line 229).

Reviewer #2 (Remarks to the Author):

Thank you for revising the manuscript and addressing the concerns raised by my review. I feel that the revised version effectively addressed most issues raised. I did find a few typos and confusing language that could be improved in the final version.

Abstract has some typos: Line 48 "live" should be "life"; line 52: seahorses are expected to have diminished long range dispersal… not "at" long range dispersal…

Response: Revised (Lines 48 and 52).

Methods in lines 428-433 describe briefly how the NJ tree for 21 seahorse species was constructed. No outgroup taxa are mentioned. This is the NJ tree shown in figure 1b, based on SNPs for 358 seahorses. The main text refers to this figure in lines 121-123, stating: "our analysis identified all seahorse species as a monophyletic group in a neighbor-joining tree…"
What is the significance of mentioning that all seahorses are in a monophyletic group if this is the only possible result, given that no outgroup taxa were included in the analysis?

Response: We are very sorry for this inaccurate description. Here we referred to each species monophyly (not the genus), as recovered in the NJ tree. We have re-phased this sentence for clarification "Our analysis identified **each** seahorse species as a monophyletic group in a neighbor-joining tree" (Line 121).

Line 192: "…ongoing gene flow from the East-African H. algiricus int the South American H . reidi… (Fig. 3a)." I believe that you really mean West-African H. algiricus or perhaps East-Atlantic H. algiricus.

Response: Yes, we have changed it to "West-African *H. algiricus*" (Line 192).

Line 206: "These findings contrast more recent studies that suggested…" These seems to be a word missing here and "more" is not necessary. Perhaps "These findings contrast with recent studies…"

Response: We have corrected this grammar error (Line 206).

Reviewer #3 (Remarks to the Author):

I have read the author's response and I am generally happy with the changes, including for the main point regarding the tests for positive selection. In the first version of the manuscript it was not clear to me that the initial CODEML dN/dS test acted as a screening step to identify candidates that were then further tested using additional methods. The authors have a very low signal in the first screen (37 genes for 20 expected by chance) but I think that this is OK given that they focus on mainly 1 gene with strong support at the end. I would however refrain from saying (line 262) that the 37 genes "show significant signals of accelerated dN/dS in spiny seahorses ($p < 0.001$)" given that there is no adjustment for multiple testing. A Benjamini-Hochberg adjustment

would cover this aspect. The authors could for example carry this out or just modify the text to remove the word “significant”.

Response: As we didn’t show Benjamini-Hochberg adjustment results, we have removed “significant” from the sentence (Line 270).
